# Methodological quality of systematic reviews in dentistry including animal studies: a cross-sectional study

**DOI:** 10.1186/s13620-023-00261-w

**Published:** 2023-12-14

**Authors:** Max C. Menne, Naichuan Su, Clovis M. Faggion

**Affiliations:** 1 Department of Prosthodontics and Biomaterials, University Hospital Münster, Waldeyerstraße 30, Münster, 48149 Germany; 2 Department of Oral and Maxillofacial Surgery, Fachklinik Hornheide, Dorbaumstraße 300, Münster, 48157 Germany; 3 Department of Oral Public Health, Academic Centre for Dentistry Amsterdam (ACTA), University of Amsterdam and Vrije Universiteit Amsterdam, Amsterdam, 1081LA The Netherlands; 4 Department of Periodontology and Operative Dentistry, University Hospital Münster, Waldeyerstraße 30, Münster, 48149 Germany

**Keywords:** Systematic reviews, Methods, Methodological study, Animal study, Preclinical study, AMSTAR-2, Methodology

## Abstract

**Background:**

The overall confidence in the results of systematic reviews including animal models can be heterogeneous. We assessed the methodological quality of systematic reviews including animal models in dentistry as well as the overall confidence in the results of those systematic reviews.

**Material & methods:**

PubMed, Web of Science and Scopus were searched for systematic reviews including animal studies in dentistry published later than January 2010 until 18th of July 2022. Overall confidence in the results was assessed using a modified version of the A MeaSurement Tool to Assess systematic Reviews (AMSTAR-2) checklist. Checklist items were rated as *yes*, *partial yes*, *no* and *not applicable*. Linear regression analysis was used to investigate associations between systematic review characteristics and the overall adherence to the AMSTAR-2 checklist. The overall confidence in the results was calculated based on the number of critical and non-critical weaknesses presented in the AMSTAR-2 items and rated as high, moderate, low and critical low.

**Results:**

Of initially 951 retrieved systematic reviews, 190 were included in the study. The overall confidence in the results was low in 43 (22.6%) and critically low in 133 (70.0%) systematic reviews. While some AMSTAR-2 items were regularly reported (e.g. conflict of interest, selection in duplicate), others were not (e.g. funding: *n* = 1; 0.5%). Multivariable linear regression analysis showed that the adherence scores of AMSTAR-2 was significantly associated with publication year, journal impact factor (IF), topic, and the use of tools to assess risk of bias (RoB) of the systematic reviews.

**Conclusion:**

Although the methodological quality of dental systematic reviews of animal models improved over the years, it is still suboptimal. The overall confidence in the results was mostly low or critically low. Systematic reviews, which were published later, published in a journal with a higher IF, focused on non-surgery topics, and used at least one tool to assess RoB correlated with greater adherence to the AMSTAR-2 guidelines.

**Supplementary Information:**

The online version contains supplementary material available at 10.1186/s13620-023-00261-w.

## Background

Research on laboratory animals, although controversially discussed [1], is a strong pillar in preclinical research and helps to understand the mechanisms of diseases and identify the efficacy and potential harm of new treatments [2, 3]. Systematic reviews of such studies can summarize their findings and improve the process of translational research [4, 5]. Following, clinical trials and systematic reviews of those bring new treatments via clinical guidelines into clinical practice [6]. To not produce misleading results, systematic reviews should follow a sound methodology.

To critically appraise the methodology of systematic reviews of randomized controlled trials, the AMSTAR tool was published and validated in 2007 [7]. In 2017 the tool was updated and now includes the assessment of non-randomized trials [8]. Since, to our knowledge, there is no distinct tool to assess the methodology of systematic reviews of trials using animals as model [9], we used the A MeaSurement Tool to Assess systematic Reviews (AMSTAR-2) checklist and adapted it for the assessment of systematic reviews including animal models.

Multiple recent publications have addressed the topic of methodological quality of systematic reviews in dentistry [10] including the fields of neuromuscular dentistry [11], implant dentistry [12–14], periodontology [15, 16], orthodontics [17], endodontics [18] and oral and maxillofacial surgery [19]. In these studies, there was substantial lack of adherence to considered critical methodological quality domains [8]. In a recent study, Hammel et al. (2022) focused on the methodological quality of systematic reviews of in-vitro dental research by using an adapted version of AMSTAR-2 [20]. They found that in the majority of included systematic reviews (68%) the overall confidence in the results was “critically low”.

The evidence on the methodological quality of systematic reviews including animal models is scarce. Mignini and Khan (2006) showed methodological weaknesses of systematic reviews including animal models and addressed the need for rigour when reviewing research involving animal models [21]. The last methodological quality assessment of systematic reviews including animal models in dentistry included publications until January 2010 and used the first version of AMSTAR [22]. Back then, of 54 included systematic reviews, only one study was scored as high quality, 17 as medium quality and 35 as low quality.

The aims of our study were twofold: 1. To assess the methodological quality and overall confidence in the results of systematic reviews of research using laboratory animals as models published on dental topics since February 2010 and, 2. To investigate the association between certain systematic review characteristics and adherence to the AMSTAR-2 checklist.

## Methods

### Eligibility criteria

We included systematic reviews including animal models in all fields of dentistry. Therefore, the focus of our work was on medical research that uses animals as models, instead of veterinary research that uses animals as subjects. Systematic reviews including both animal and human studies were also included. Study designs other than systematic reviews and systematic reviews with non-pair-wise meta-analyses were excluded (for example, network meta-analysis). An article was considered a systematic review if it was titled as such, or if the authors´ aim was to perform a systematic review. Publications in other languages than English were excluded.

### Search strategy

On the 18th of July 2022, we searched the Pubmed, Scopus and Web of Science databases for systematic reviews published in the field of dentistry including animal models. We used a combination of key-words and Boolean operators and limited our search studies published after January 2010 to 18th of July 2022. This cut-off time point was chosen to provide an updated assessment of a previous study on the methodological quality of systematic reviews of dental animal studies [22].

We adapted the syntax of the search performed in PubMed (Table S1, supplementary file) for the Scopus and Web Of Science databases. The search was done in duplicate and independently by two authors to ensure reproducibility (MCM, CMF). If the searches produced the same findings the search was deemed reproducible. The search strategies are reported in Supplementary file 1.

### Selection process

We selected articles strictly based on the eligibility criteria, and articles not meeting these criteria were excluded with individual reasons recorded in each phase of the assessment. First, duplicates were removed assisted by the Zotero citation manager (Roy Rosenzweig Center for History and New Media, George Mason University). Following that, we checked the title and abstract of all findings. Lastly, we checked the full-text of the remaining studies. The last 2 processes were done in duplicate and independently by two reviewers (MCM, CMF) for 30 samples and discussed until good agreement on inclusion or exclusion (at least 80%) of articles was reached [8], then the remaining selection was done by one reviewer (MCM).

### Data collection process

To give an overview of the assessed systematic reviews and to find associations between methodological quality and study characteristics different objectifiable measures were defined and collected. Of each systematic review, we collected the following characteristics: h-index of first and last author (checked in Scopus on the 30th of April 2023), number of authors, continent of first author, country (region) of first author, year of publication, journal’s name, journal category in Journal Citation Reports, 2-year journal impact factor (2021 Journal Impact Factor, Journal Citation Reports (Clarivate, 2022), topic of study, presence of conflict of interest, type of funding/sponsorship, number of citations (checked in google scholar on the 30th of April 2023), and tools used for Risk of Bias (RoB) assessment.

Data collection was done with a previously created sheet in Microsoft Excel (Microsoft Corporation) in duplicate and independently by two reviewers (MCM, CMF) for 30 samples and discussed until good agreement about the extracted characteristics and methodological quality assessment in terms of AMSTAR-2 scores (at least 80%) was reached [8], then the remaining data collection was done by one reviewer (MCM).

### AMSTAR-2 items

For the assessement of the methodological quality of the included systematic reviews we used the AMSTAR-2 tool. The ckecklist includes 16 items which allow for a critical appraisal of systematic reviews of randomised and non-randomised studies of healthcare interventions. AMSTAR-2 is considered a valid and reliable appraisal tool [23]. We adapted AMSTAR-2 slightly to allow for the assessment of trials using animal models (details are reported in the Supplementary file 2). Our evaluation criteria for each item are based on the AMSTAR-2 guidance document (Supplementary file 1 of the AMSTAR-2 publication) [8]. Our adapted checklist did not require the registration of the systematic review protocols since existent registries were not available in the entire timeframe of our search. Also, to meet the requirements for item 3, both an explanation of the selection of the study design and the study population (i.e. animals) would be necessary. Since RoB assessment of studies using animals as models is difficult and SYRCLE’s tool has just been published in 2014, we accepted alternative approaches to assess RoB. Lastly, we added a secound rank to the AMSTAR-2 criteria in the assessment of heterogeneity allowing for a more differentiated view of this criterion. Checklist items were answered as yes (when all checklist criteria were met), partial yes (when some criteria were met), and no (when no criteria were met). If we were not able to rate a checklist item it was documented as “not applicable”. A detailed description of our evaluation is reported in Supplementary file 2.

For each included study, an adherence score was calculated as the number of items answered as “yes” and “partial yes” / number of total applicable items * 100. A higher adherence score indicates a better methodological quality. In addition, the overall confidence in the results of the reviews was categorised into high, moderate, low, and critically low, based on AMSTAR-2 [8]. If at most one non-critical items from AMSTAR-2 was answered with “no” but none of the critical items was answered with “no”, the overall confidence was considered high. If more than one non-critical item was answered with “no” but none of the critical items was answered with “no”, the overall confidence in the results was considered moderate. The overall confidence was considered low or critically low if one or more than one critical item was answered with “no”, respectively.

### Statistical analysis

To facilitate the statistical analysis, “country of first author” was dichotomized into “developing country” and “developed country” based on the World Economic Situation and Prospects 2023 of United Nations [24] and “Topic of study” was dichotomized into “non-surgical topic” and “surgical topic”. To assess the association between study characteristics (independent variables) and methodological quality of the included studies (i.e. adherence scores), linear regression analyses were used.

First, univariable linear regression analysis was performed to assess the association of each independent variable with adherence scores, separately. Second, multicollinearity of the independent variables which were significant in the univariable analyses (*P* < 0.05) was tested using the variance inflation factor (VIF) before they were included in the subsequent multivariable linear regression analysis. When a VIF value of a variable was higher than 5, collinearity was considered present and the variable was excluded from the following analysis [25]. Third, a multivariable linear regression analysis with backward selection was performed to further assess the association between independent variables and adherence scores. In the multivariable analysis, the variables with the highest *p* values were removed first from the model and the cut-off *p* value for removal was 0.05.

## Results

### Study selection

**Fig. 1:**
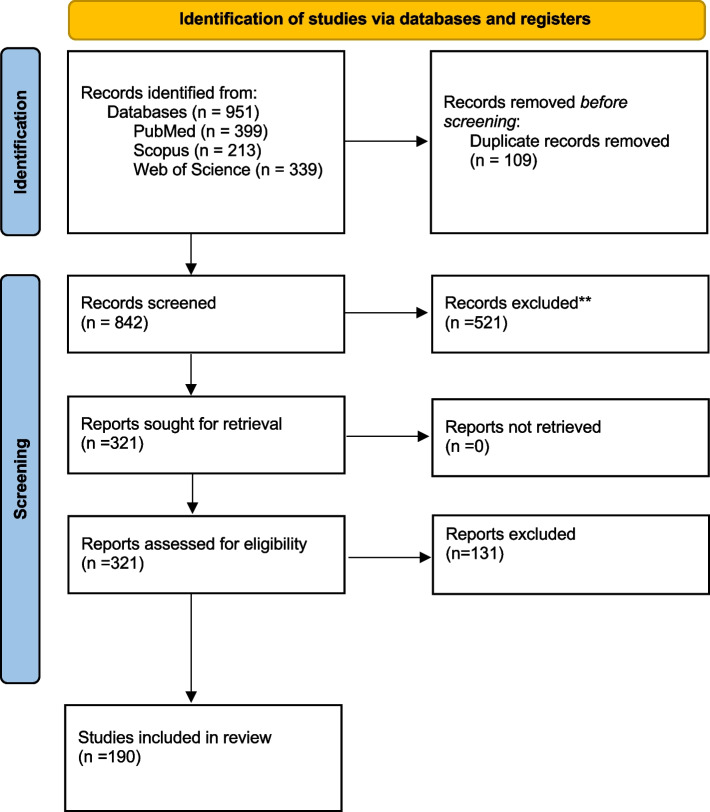
PRISMA Flow diagram

Our search identified 951 records overall. Before screening 109 duplicates were removed. After screening of titles and abstracts, 521 records were excluded. In the step of full-text screening, 131 additional records were excluded. Finally, 190 records were included in this study (Fig. 1). The excluded studies with reasons for exclusion and the included studies are reported in the supplementary files 3 and 4, respectively.

### Study characteristics

The mean and standard deviation (SD) of the h-index of the first author was 11.81 ± 12.81 (Range 0–69), the one of the last author 28.98 ± 18.12 (Range 1–117). The mean number of authors was 4.9 ± 1.7 (Range 1–9). Over one third of the first authors were located in Europe (*n* = 72; 37.9%), even though the most prevalent country of first authors was Brazil (*n* = 30; 15.8%). The major part of systematic reviews was conducted by multi-center cooperation (*n* = 159; 83.7%). The most systematic reviews were published in 2018 (*n* = 32; 16.9%). Systematic reviews were published most often in Archives of Oral Biology (*n* = 18; 9.47%). About two thirds of the systematic reviews were published in the journal category: DENTISTRY, ORAL SURGERY & MEDICINE – SCIE (*n* = 122; 64.2%). The mean Journal impact Factor was 3.747 ± 1.474 (Range 1.154–8.755). About a half of the systematic reviews´ topic was Oral Surgery and Implant Dentistry (*n* = 84; 44.2%). Most of the systematic reviews reported to have no conflict of interest (*n* = 151; 79.5%). Seventy systematic reviews were not sponsored (36.8%), closely followed by 67 systematic reviews that did not provide clear information on funding (35.3%). On average the included systematic reviews were cited 38.48 ± 61.01 times (Range 0–493). The most often used RoB assessment tool was SYRCLE (*n* = 71; 29.1%). The complete characteristics are reported in Supplementary file 5.

### Methodological quality - AMSTAR-2 items

Overall, two (1.1%) systematic reviews presented high, 12 (6.3%) medium, 43 (22.6%) low and 133 (70.0%) critically low confidence in the results.

The assessment revealed great heterogeneity in reporting of the different checklist items.

The checklist items 16 (“Did the review authors report any potential sources of conflict of interest, including any funding they received for conducting the review?”) and 5 (“Did the review authors perform study selection in duplicate?”) were the most often in full accordance to the checklist (80.5 and 78.4% respectively). The item 4 (Did the review authors use a comprehensive literature search strategy?) was the item with the greatest percentage (87.9%) of answers with “partial yes”. Only one systematic review considered the funding of primary studies and reported it (0.5%). Results of all assessed items are described in Table 1.

**Table 1:** Evaluation of the methodological quality of the included reviews based on AMSTAR-2

AMSTAR items	Yes	Partial Yes	No	Not applicable
Did the research questions and inclusion criteria for the review include the components of PICO?	128 (67.4%)	0 (0%)	62 (32.6%)	0 (0%)
Did the report of the review contain an explicit statement that the review methods were established prior to the conduct of the review and did the report justify any significant deviations from the protocol?	119 (62.6%)	0 (0%)	71 (37.4%)	0 (0%)
Did the review authors explain their selection of the study designs for inclusion in the review?	6 (3.2%)	48 (25.3%)	136 (71.6%)	0 (0%)
Did the review authors use a comprehensive literature search strategy?	2 (1.1%)	167 (87.9%)	21 (11.1%)	0 (0%)
Did the review authors perform study selection in duplicate?	149 (78.4%)	0 (0%)	41 (21.6%)	0 (0%)
Did the review authors perform data extraction in duplicate?	67 (35.3%)	0 (0%)	123 (64.7%)	0 (0%)
Did the review authors provide a list of excluded studies and justify the exclusions?	68 (35.8%)	0 (0%)	122 (64.2%)	0 (0%)
Did the review authors describe the included studies in adequate detail?	53 (27.9%)	111 (58.4%)	25 (13.2%)	1 (0.5%)
Did the review authors use a satisfactory technique for assessing the risk of bias (RoB) in individual studies that were included in the review?	83 (43.7%)	29 (15.3%)	78 (41.1%)	0 (0%)
Did the review authors report on the sources of funding for the studies included in the review?	1 (0.5%)	0 (0%)	188 (98.9%)	1 (0.5%)
If meta-analysis was performed did the review authors use appropriate methods for statistical combination of results?	43 (22.6%)	0 (0%)	1 (0.5%)	146 (76.8%)
If meta-analysis was performed, did the review authors assess the potential impact of RoB in individual studies on the results of the meta-analysis or other evidence synthesis?	5 (2.6%)	0 (0%)	39 (20.5%)	146 (76.8%)
Did the review authors account for RoB in individual studies when interpreting/ discussing the results of the review?	36 (18.9%)	0 (0%)	154 (81.1%)	0 (0%)
Did the review authors provide a satisfactory explanation for, and discussion of, any heterogeneity observed in the results of the review?	89 (46.8%)	30 (15.8%)	70 (36.8%)	1 (0.5%)
If they performed quantitative synthesis did the review authors carry out an adequate investigation of publication bias (small study bias) and discuss its likely impact on the results of the review?	19 (10.0%)	5 (2.6%)	20 (10.5%)	146 (76.8%)
Did the review authors report any potential sources of conflict of interest, including any funding they received for conducting the review?	153 (80.5%)		37 (19.5%)	0 (0%)

### Methodological quality and study characteristics

**Fig. 2:**
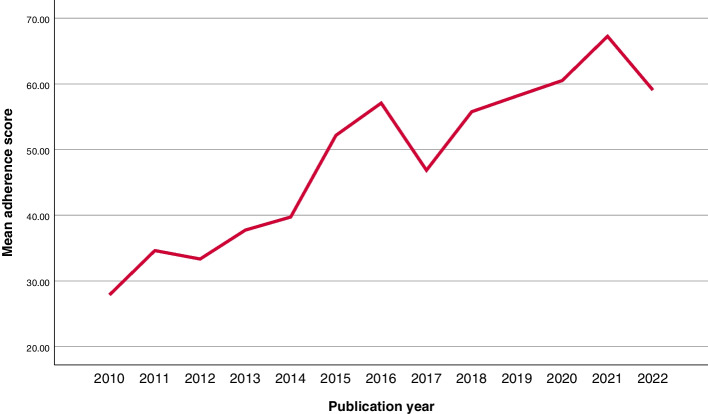
Adherence score development over time

Table 2 presents the general characteristics of the included reviews and the corresponding scores. The adherence scores of the included systematic reviews increased over time (Fig. 2). Based on the univariable linear regression analyses, publication year (*P* < 0.01), journal impact factor (*P* < 0.01), topic of study (*P* = 0.03), number of citations (*P* < 0.01), and number of tools used (*P* < 0.01) were significantly associated with the adherence scores of the reviews (Table 3). The VIF values of the significant variables in the univariable analyses were all < 4, indicating that the collinearity was absent. Therefore, those variables were included in the subsequent multivariable linear regression analysis. In the multivariable linear regression analysis with backward selection, adherence score was significantly associated with publication year (β: 1.59; 95%CI: 0.84–2.33; *P* < 0.01), journal impact factor (β: 2.96; 95%CI: 1.57–4.34; *P* < 0.01), topic of study (β: 4.75; 95%CI: 0.31–9.18; *P* = 0.04), and number of tools used (for 1 tool: β: 25.59; 95%CI: 20.31–30.88; *P* < 0.01, for > 1 tool: β: 26.65; 95%CI: 20.43–32.86; *P* < 0.01) (Table 3).

**Table 2:** General characteristics of the included reviews and the corresponding adherence scores

Variables	N (%)	Mean	SD	95%CI
H index of 1st author				
≤ 20	156 (83.0%)	55.09	20.49	51.85–58.33
20–40	20 (10.6%)	49.13	23.12	38.32–59.95
> 40	12 (6.4%)	49.80	19.71	37.27–62.32
H index of last author				
≤ 20	69 (37.1%)	55.72	20.30	50.85–60.60
20–40	75 (40.3%)	53.46	21.11	48.60–58.32
> 40	42 (22.6%)	53.78	20.50	47.39–60.17
Number of authors				
1–3	46 (24.2%)	51.77	23.59	44.77–58.78
4–5	74 (38.9%)	55.68	21.40	50.72–60.64
≥ 6	70 (36.8%)	53.24	18.23	48.89–57.59
Continent of primary authors				
North America	27 (14.2%)	53.85	18.45	46.55–61.15
South America	32 (16.8%)	55.43	20.77	47.95–62.92
Europe	72 (37.9%)	52.94	21.81	47.82–58.07
Asia	49 (25.8%)	53.87	20.80	47.90–59.85
Africa	1 (0.5%)	87.50	NA	NA
Australia	9 (4.7%)	51.28	21.76	34.56–68.01
Country (or region)				
Australia	9 (4.7%)	51.28	21.76	34.56–68.01
Belgium	6 (3.2%)	50.00	16.68	32.50–67.50
Brazil	30 (15.8%)	53.74	20.12	46.23–61.26
Canada	1 (0.5%)	84.62	NA	NA
Chile	1 (0.5%)	92.31	NA	NA
China	10 (5.3%)	50.65	20.64	35.88–65.41
Colombia	1 (0.5%)	69.23	NA	NA
Denmark	2 (1.1%)	62.02	0.68	55.91–68.12
Egypt	1 (0.5%)	87.50	NA	NA
Finland	1 (0.5%)	7.69	NA	NA
France	5 (2.6%)	44.42	27.47	10.31–78.53
Germany	7 (3.7%)	49.93	27.37	24.61–75.25
Greece	4 (2.1%)	50.00	28.44	4.75–95.25
India	7 (3.7%)	46.15	27.01	21.17–71.14
Iran	5 (2.6%)	33.94	11.64	19.49–48.39
Italy	10 (5.3%)	54.66	20.66	39.89–69.44
Japan	1 (0.5%)	50.00	NA	NA
Lithuania	1 (0.5%)	61.54	NA	NA
Netherlands	6 (3.2%)	65.06	21.52	42.48–87.65
Norway	3 (1.6%)	67.79	14.90	30.77–104.81
Pakistan	1 (0.5%)	38.46	NA	NA
Poland	1 (0.5%)	56.25	NA	NA
Portugal	1 (0.5%)	30.77	NA	NA
Romania	1 (0.5%)	69.23	NA	NA
Saudi-Arabia	13 (6.8%)	50.41	15.76	40.89–59.93
Singapore	1 (0.5%)	84.62	NA	NA
South Korea	1 (0.5%)	56.25	NA	NA
Spain	13 (6.8%)	46.23	19.17	34.64–57.81
Sweden	4 (2.1%)	53.61	22.13	18.40–88.82
Switzerland	6 (3.2%)	60.02	26.29	32.43–87.61
Taiwan	1 (0.5%)	62.50	NA	NA
UAE	9 (4.7%)	77.05	5.59	72.76–81.35
UK	1 (0.5%)	75.00	NA	NA
USA	26 (13.7%)	52.66	17.74	45.50–59.83
Country (or region)				
Developing	81 (42.6%)	54.95	20.99	50.31–59.59
Developed	109 (57.4%)	53.00	20.74	49.07–56.94
Center				
Single-center	31 (16.3%)	52.00	22.76	43.65–60.35
Multi-center	159 (83.7%)	54.19	20.47	50.99–57.40
Publication year				
2010–2015	37 (19.5%)	37.85	22.20	30.45–45.25
2016–2018	73 (38.4%)	53.28	18.47	48.97–57.59
2019–2022	80 (42.1%)	61.74	17.83	57.77–65.70
Journal category				
Dental journals (ESCI)	11 (5.8%)	42.13	26.37	24.41–59.85
Dental journals (SCIE)	122 (64.2%)	54.25	20.01	50.66–57.83
Other dental journals	8(4.2%)	41.75	27.23	18.98–64.51
Non-dental journals	49 (25.8%)	57.41	19.35	51.85–62.97
Journal category				
Dental journals	141 (74.2%)	52.59	21.22	49.06–56.13
Non-dental journals	49 (25.8%)	57.41	19.35	51.85–62.97
Journal impact factor				
≤ 3	69 (40.8%)	49.65	18.95	45.10–54.20
3–5	71 (42.0%)	58.60	18.81	54.15–63.05
> 5	29 (17.2%)	60.69	21.69	52.44–68.94
Topic				
Oral surgery + implant dentistry	84 (44.2%)	51.70	20.26	47.30–56.10
Periodontology + periodontal surgery	42 (22.1%)	51.26	21.87	44.44–58.07
Conservative dentistry + endodontics	24 (12.6%)	54.95	23.33	45.10–64.80
Prosthodontics	2 (1.1%)	69.23	0.00	69.23–69.23
Orthodontics	38 (20.0%)	59.89	18.83	53.70–66.08
Topic				
Surgery	126 (66.3%)	51.55	20.73	47.90–55.21
Non-surgery	64 (33.7%)	58.33	20.41	53.23–63.43
Funding				
Funded	53 (27.9%)	53.96	16.91	49.30–58.62
No funding	70 (36.8%)	60.43	19.50	55.78–65.08
No information	67 (35.3%)	46.85	22.86	41.27–52.43
Number of citations				
0–10	58 (30.5%)	59.91	19.51	54.79–65.04
11–50	92 (48.4%)	55.61	18.06	51.87–59.35
51–100	27 (14.2%)	42.02	20.63	33.86–50.18
> 100	13 (6.8%)	38.65	29.24	20.98–56.31
Number of tools used				
No tool	49 (25.8%)	29.78	15.05	25.45–34.10
1 tool	95 (50.0%)	62.02	16.39	58.68–65.36
> 1 tool	46 (24.2%)	62.56	12.94	58.72–66.40

*ESCI* Emerging Sources Citation Index, *SCIE* Science Citation Index Expanded, *NA* Not applicable, *SD* standard deviation

**Table 3:** Regression analysis for the association between study characteristics and the adherence scores

	Univariable			Multivariable		
	β	95%CI	P	β	95%CI	P
H index of 1st author	−0.22	−0.46-0.01	0.06			
H index of last author	−0.10	−0.27-0.06	0.22			
Number of authors	0.27	−1.44-1.98	0.76			
Country						
Developing	Ref.					
Developed	−1.95	−7.98-4.08	0.53			
Center						
Single	Ref.					
Multiple	2.19	−5.89-10.27	0.59			
Publication year	3.10	2.20–4.00	< 0.01*	1.59	0.84–2.33	< 0.01*
Journal category						
Dental journals	Ref.					
Non-dental journals	4.82	−1.97-11.61	0.16			
Impact factor	3.14	1.14–5.14	< 0.01*	2.96	1.57–4.34	< 0.01*
Topic						
Surgery	Ref.			Ref.		
Non-surgery	6.78	0.53–13.02	0.03*	4.75	0.31–9.18	0.04*
Funding						
Funded	Ref.					
No funding	6.47	−0.75–13.69	0.08			
No information	−7.11	−14.40-0.18	0.06			
Citations	−0.12	− 0.16--0.07	< 0.01*			
Number of tools used						
No tool	Ref.			Ref.		
1 tool	32.24	26.94–37.54	< 0.01*	25.59	20.31–30.88	< 0.01*
> 1 tool	32.78	26.60–38.97	< 0.01*	26.65	20.43–32.86	< 0.01*

*Statistically significant

## Discussion

### Main findings

This study is to our knowledge the first one to assess the methodological quality of the current dental literature on systematic reviews of dental experiments on laboratory animals. The last study to do so was published in 2012 and used the previous version of the AMSTAR [22]. The results of the present study suggest that the methodological quality of these systematic reviews improved over the years, however, there is still room for improvement. Most systematic reviews were rated with low to critically low confidence in the results. The systematic reviews that were published later, published in a journal with higher impact factor, focused on non-surgery topics and used at least one tool had significantly higher adherence scores.

### Interpretation of the results

We found that the included studies presented heterogeneous methodological quality. About two-thirds of the included studies used a PICO methodology. The PICO- or its adaptations is a well-established format that helps to orientate the research question and translate it into a reliable bibliographic search; therefore its use is recommended [26].

The second AMSTAR-2 item regarding the prior establishment of review methods was completely met in 61% of the systematic reviews. It is important to note, that it does not mean, that these methods were sound. We did not require the protocols to be published in online databases like PROSPERO since they were not available in the whole timeframe of the included articles. Also, they did not always allow for the registration of systematic reviews including both, animal and human trials [27].

Only six studies explicitly explained their selection of the included study design and population, 48 did either of both, and 136 did neither of both. Reporting this information is important to let the reader know in which research phase the treatment currently is and give information on possible limitations [28].

The literature strategy was only reported completely in two systematic reviews while most systematic reviews (*n* = 167; 88%) conducted a literature search that included the minimum requirements. Twenty-one systematic reviews scored a *no* in this item. Following the AMSTAR-2 guidance, the criteria for a *yes* are very strict. Authors need to search at least two databases, provide keywords and/ or search strategy, justify publication relevant publication restrictions (i.e. language or timeframe) (*partial yes*) and search reference lists of included studies, search trial registries, consult experts in the field, search for grey literature and conduct the search within 24 months of completion of the review. These strict criteria suggested by AMSTAR-2 might be challenging to fulfill. However, a sound search strategy builds the base of a solid systematic review and helps reduce bias; therefore as much of the above-mentioned criteria should be met by authors [29–33].

Data selection and extraction should be done independently and in duplicate for a representative amount of systematic reviews to reduce the risk of potential mistakes [34]. In the assessed data sample, selection and extraction were done in duplicate in 149 (78%) and 67 (35%) systematic reviews, respectively. This approach is in line with items 5 and 6 from the original AMSTAR-2 checklist.

To make a systematic review transparent and reproducible it is important to provide lists with excluded studies. AMSTAR-2 recommends reporting the full list of excluded studies after full-text assessment with the respective individual reasons for exclusion [8]. Some authors suggest an even stricter form of reporting to allow better reproducibility [35] by requiring the report of reasons for extraction since the title/abstract phase selection. In this project, we followed the AMSTAR-2 guidance and scored *yes* if the authors provided a list of excluded articles only after full-text assessment with reasons for exclusion. Only about a third *n* = 68; 36%) scored a *yes*, the rest did score a *no* (*n* = 122; 64%).

Little over three-quarters of the included systematic reviews provided either enough or detailed information about the included studies (yes: *n* = 53, 28%; partial yes: *n* = 111, 58%), while only 13% (*n* = 25) did not. The distinction between *yes* and *partial yes* was difficult for this item. For a *yes* the major part of the categories: population, intervention and comparator should be described in detail. However, particularly in research performed in laboratory animal models, the description of the included population is important since different animals might react differently to different therapies. This might also differ for age, weight, or sex [36–38]. Since treatment effects can also be dependent on follow-up and study setting this information should also be included in the systematic review.

RoB assessment is one of the central points of a sound systematic review [39]. The assessment of RoB of primary studies included in the systematic review involves the appraisal of potential limitations or problems in study domains that may influence or bias the estimates of this study. Studies having a high RoB may generate overestimated effect sizes [40, 41]. It is therefore important use the results of the RoB assessment to critically appraise the results of primary studies and put them into context of each other [42]. We adapted the assessment compared to the AMSTAR-2 criteria. For a *yes* authors would have needed to use an adequate RoB tool and report the results per primary study included (*n* = 83; 44%); if they reported only an overall score for all studies, they would be considered *partial yes* (*n* = 29; 15%). If authors used reporting guidelines for RoB assessment or did not perform it, they were rated as *no* (*n* = 78, 41%). Authors used 27 different tools to assess RoB with the most frequently one used being SYRCLE (*n* = 71; 29.1%). Even though almost 60% of the systematic reviews scored at least a *partial yes* in the RoB assessment, only 19% (*n* = 36) accounted for possibly detected bias when interpreting or discussing the results of the review.

Heterogeneity is the variability among studies included in the systematic review that may impact systematic review results. The literature describes three types of heterogeneity: clinical, when there is variability in the PICO format of primary studies [43, 44], statistical, when there is variability in the intervention effects [45], and methodological, when studies included have differences in terms of study design and RoB ratings. It is important to discuss heterogeneity to understand how clinical and methodological aspects of the primary studies relate to the systematic review results, mainly in case a meta-analysis is conducted. In our sample about half of the systematic reviews (*n* = 89; 47%) discussed found heterogeneity and considered its impact on the results. Thirty (16%) systematic reviews mentioned the existence of heterogeneity or mentioned that they did not perform meta-analysis due to high heterogeneity among studies, while 70 (37%) did not mention heterogeneity at all.

Some evidence suggests that financial and non-financial conflicts of interest can influence study results [46–48], and therefore clear reporting of this information is necessary. Of the included systematic reviews 84% (*n* = 159) provided clear information on conflicts of interest. Only one systematic review reported information on the funding of included primary studies.

Three of the AMSTAR-2 items were specifically designed for the assessment of meta-analyses (items 11,12 and 15). Of the included 190 systematic reviews only 45 performed meta-analyses. Of those, 43 (95%) described a sound methodology for the conduction of the meta-analysis, appropriate weighting techniques and investigated causes of heterogeneity. Like with the discussion of found RoB, authors also did not frequently assess the impact of heterogeneity of individual studies on the meta-analysis estimates (*n* = 6; 13%).

Publication bias describes the failure to publish the results of a study based on the direction or estimate of the study findings [49]. This can lead to an overestimation of subsequent meta-analysis effects [50]. Therefore, investigation of publication bias is important to understand how much a meta-analysis estimate deviates from its real value. In our sample of systematic reviews with meta-analysis, 19 (42%) performed investigations for publication bias and discussed them or planned the investigation but were not able to do it because of the limited number of included studies. Cochrane states that tests for funnel plot asymmetry need at least 10 studies to have enough statistical power [51]. Therefore, in some cases the implementation of such tests can be problematic.

Regression analysis demonstrated that more recent systematic reviews presented higher methodological quality than older ones. This might be explained by a greater continuous awareness of the medical/dental community regarding methodological aspects of research. Also, journals with higher IF published systematic reviews with higher methodological quality. This finding is in agreement with other studies published in different medical fields [52–54]. Systematic reviews using more than one tool to assess the RoB of primary studies included also presented higher quality scores. One hypothesis to explain this finding is the willingness of authors to provide a comprehensive view of the evidence through the application of different methodological tools that might imply a stronger methodological background of these authors.

### Comparison to the results of Faggion et al. 2012 [22]

Comparing the findings of the current study and the study from 2012, we can see improvements in nine of the ten comparable checklist items. The complete table is reported in Supplementary file 6. These improvements are supported by the regression analysis showing that the adherence to the AMSTAR-2 guidelines improved by year (β: 1.59; 95%CI: 0.84–2.33; *P* < 0.01) (see also Fig. 2).

### Comparison to systematic reviews not including animals

Several studies have also addressed the topic of methodological quality of systematic reviews of clinical studies in dentistry [10–20, 55–57]. Generally, these overviews also concluded that there is room for improving their methodologies. For example, many of the overviews of systematic reviews of clinical studies in dentistry reported over 50% of reviews with low to critically low confidence in the results [10–12, 16, 18, 20]. These results are in agreement with our study that rated more than 90% of the systematic reviews included with low to critically low confidence in the results. However, a direct comparison is challenging because the AMSTAR-2 items needed to be modified to adapt to a different scenario (animals vs. humans). However, many of the original items can be applied to any type of systematic review, for example, those items related to selection and data extraction.

### Relevance of the present findings and further recommendations

Studies using laboratory animals as research models can be ethically controversial [1]. Therefore, the primary studies themselves, but also following research such as systematic reviews have to be of the highest quality to justify this kind of research. The present study adds value to the scientific community by increasing awareness of researchers on the importance of methodological quality when they plan and conduct a systematic review of animal models. Some items can be improved by increasing awareness of reporting adherence (for example, item 16 on CoI). However, other items will need a more careful plan and therefore the help of colleagues specialized in specific areas such as librarians (for example, when conducting comprehensive searches), experienced statisticians (when deciding, planning and conducting meta-analyses), and methodologists (when planning more complex systematic reviews, for example systematic reviews of complex interventions [58]).

Improvements in the methodological quality of systematic reviews of pre-clinical studies will more accurately inform the benefits and harm of potential therapies as well as identify the need for further studies performed in animal models about some specific topic. This improvement in methodological quality will facilitate the translational process from preclinical to clinical research.

### Strengths and limitations

The present study has some limitations. We only included studies published in English, therefore some studies might have been excluded. We did not make this limitation in the search process, but in the selection and excluded one study. Apart from that, the greatest part of studies indexed in online databases is published in English [59] and research has shown that restricting research to English-language publications might only have little impact [60]. Additionally, the AMSTAR-2 checklist we used was not developed for the assessment of systematic reviews including animal studies. Some items had to be adapted, however, this process was transparently reported in the manuscript and the supplementary files.

Apart from those limitations, this study has definite strengths. This study is one of the few studies addressing the topic of methodological quality of systematic reviews including dental animal models. We also used robust methodological standards to develop this study and the sample of systematic reviews included appears to be representative of dental animal model studies.

## Conclusions

Although the methodological quality of systematic reviews of experiments on dental laboratory animal models improved over the years, there is still room for improvement in different systematic review domains. The methodological limitations in these domains were the explanation for the low and critically low overall confidence in the results for most of the systematic reviews in the present sample. Year of publication, journal impact factor, number of tools used and topic were significant predictors for adherence to the AMSTAR-2 items.

### Supplementary Information

**Additional file 1.** Literature Search Strategy.

**Additional file 2.** Adapted AMSTAR-2 Checklist.

**Additional file 3.** Excluded articles.

**Additional file 4.** Included articles.

**Additional file 5.** Systematic review characteristics.

**Additional file 6.** Comparison to Faggion et al. 2012.

## Data Availability

Data and materials are available upon request.

## Abbreviations

AMSTAR-2: A MeaSurement Tool to Assess systematic Reviews
RoB: Risk of bias
VIF: Variance inflation factor
SD: Standard deviation
