# Supplementary material for: Methodological quality of systematic reviews in dentistry including animal studies: a cross-sectional study
Source: Ir Vet J. 2023 Dec 14;76:33. doi: 10.1186/s13620-023-00261-w (PMC10720166; doi:10.1186/s13620-023-00261-w)
Supplement: Supplementary file 1 — Additional file 1. Literature Search Strategy. [file 13620_2023_261_MOESM1_ESM.docx]

Supplementary file 1 – Literature Search Strategy

PubMed

| **#** | **Query** | **Findings** |
| --- | --- | --- |
| 7 | #1 AND #2 AND #3 Filters: Meta-Analysis, Systematic Review, Other Animals | 399 |
| 6 | #1 AND #2 AND #3 Filters: Meta-Analysis, Systematic Review | 10,090 |
| 5 | #1 AND #2 AND #3 Filters: Meta-Analysis | 5,181 |
| 4 | : #1 AND #2 AND #3 | 250,258 |
| 3 | ("2010/02/01"[Date - Publication] : "3000"[Date - Publication]) | 14,289,995 |
| 2 | dentistry OR orthodontics OR endodontics OR prosthodontics OR "dental implants" OR peri-implant* OR "oral surgery" OR "pediatric dentistry" OR pedodontics OR "oral pathology" OR periodontics OR "oral medicine" | 758,619 |
| 1 | mouse OR mice OR rat OR rats OR pig OR pigs OR sheep OR swine OR swines OR monkey OR monkeys OR goat OR goats OR cat OR cats OR dog OR dogs OR rabbit OR rabbits | 24,332,576 |

Scopus

| **#** | **Query** | **Findings** |
| --- | --- | --- |
| 5 | ( TITLE-ABS-KEY ( mouse OR mice OR rat OR rats OR pig OR pigs OR sheep OR swine OR monkey OR monkeys OR goat OR goats OR cat OR cats OR dog OR dogs OR rabbit OR rabbits ) ) AND ( TITLE-ABS-KEY ( dentistry OR orthodontics OR endodontics OR prosthodontics OR "dental implants" OR peri-implant* OR "oral surgery" OR "pediatric dentistry" OR pedodontics OR "oral pathology" OR periodontics OR "oral medicine" ) ) AND ( PUBYEAR > 2009 AND NOT PUBDATETXT ( january 2010 ) ) AND ( LIMIT-TO ( DOCTYPE , "re" ) ) | 213 |
| 4 | ( TITLE-ABS-KEY ( mouse OR mice OR rat OR rats OR pig OR pigs OR sheep OR swine OR monkey OR monkeys OR goat OR goats OR cat OR cats OR dog OR dogs OR rabbit OR rabbits ) ) AND ( TITLE-ABS-KEY ( dentistry OR orthodontics OR endodontics OR prosthodontics OR "dental implants" OR peri-implant* OR "oral surgery" OR "pediatric dentistry" OR pedodontics OR "oral pathology" OR periodontics OR "oral medicine" ) ) AND ( PUBYEAR > 2009 AND NOT PUBDATETXT ( january 2010 ) ) | 6507 |
| 3 | PUBYEAR > 2009 AND NOT PUBDATETXT ( january 2010 ) | 38,944,654 |
| 2 | TITLE-ABS-KEY ( dentistry OR orthodontics OR endodontics OR prosthodontics OR "dental implants" OR peri-implant* OR "oral surgery" OR "pediatric dentistry" OR pedodontics OR "oral pathology" OR periodontics OR "oral medicine" ) | 280,302 |
| 1 | TITLE-ABS-KEY ( mouse OR mice OR rat OR rats OR pig OR pigs OR sheep OR swine OR monkey OR monkeys OR goat OR goats OR cat OR cats OR dog OR dogs OR rabbit OR rabbits ) | 5,770,683 |

Web of Science

| **#** | **Query** | **Findings** |
| --- | --- | --- |
| 5 | #1 AND #2 AND #3 and Review Articles (Document Types) and Dentistry Oral Surgery Medicine (Web of Science Categories) | 339 |
| 4 | #1 AND #2 AND #3 | 48,781 |
| 3 | DOP=(2010-02-01/2022-07-18) | 30,082,841 |
| 2 | ALL=(dentistry OR orthodontics OR endodontics OR prosthodontics OR "dental implants" OR peri-implant* OR "oral surgery" OR "pediatric dentistry" OR pedodontics OR "oral pathology" OR periodontics OR "oral medicine" ) | 763,243 |
| 1 | ALL=(mouse OR mice OR rat OR rats OR pig OR pigs OR sheep OR swine OR monkey OR monkeys OR goat OR goats OR cat OR cats OR dog OR dogs OR rabbit OR rabbits ) | 4,603,737 |
