# Supplementary material for: Methodological quality of systematic reviews in dentistry including animal studies: a cross-sectional study
Source: Ir Vet J. 2023 Dec 14;76:33. doi: 10.1186/s13620-023-00261-w (PMC10720166; doi:10.1186/s13620-023-00261-w)
Supplement: Supplementary file 2 — Additional file 2. Adapted AMSTAR-2 Checklist. [file 13620_2023_261_MOESM2_ESM.docx]

Supplementary file 2 – Adapted AMSTAR-2 Checklist

1. Did the research questions and inclusion criteria for the review include the components of PICO?

| Yes (All – or adequate to the research question) | No |
| --- | --- |
| **P**opulation | Not reported |
| **I**ntervention |  |
| **C**omparator group |  |
| **O**utcome |  |

2. Did the report of the review contain an explicit statement that the review methods were established prior to the conduct of the review and did the report justify any significant deviations from the protocol?

*Adaptation: Registration of the protocol is not required since online registries like CAMRADES or PROSPERO weren´t available in the whole timeframe. Also, some databases only allow for the registration of systematic reviews including either animals or humans as population.*

| Yes (Partial Yes plus) | No |
| --- | --- |
| a meta-analysis/synthesis plan, if appropriate* | Not reported |
| a plan for investigating causes of heterogeneity* |  |
| review question(s) (PICO) |  |
| a search strategy |  |
| inclusion/exclusion criteria |  |
| a risk of bias assessment |  |

*only for systematic reviews including meta-analysis

3. Did the review authors explain their selection of the study designs for inclusion in the review?

*Adaptation: Different animal models might influence the results of the study. Therefore, we requested an explanation of why the population was assessed.*

| Yes | Partial Yes | No |
| --- | --- | --- |
| Explanation for included design and study population | Explanation for either included design or study population | Not reported |

4. Did the review authors use a comprehensive literature search strategy?

| Yes (= Partial Yes plus) | Partial Yes (at least first 2) | No |
| --- | --- | --- |
| searched the reference lists / bibliographies of included studies | searched at least 2 databases (relevant to research question) | Not reported |
| searched trial/study registries | provided key word and/or search strategy |  |
| included/consulted content experts in the field | justified publication restrictions (e.g. language) |  |
| where relevant, searched for grey literature |  |  |
| conducted search within 24 months of completion of the review |  |  |

5. Did the review authors perform study selection in duplicate?

| Yes | No |
| --- | --- |
| at least two reviewers independently agreed on selection of eligible studies and achieved consensus on which studies to include | Not reported |
| two reviewers selected a sample of eligible studies and achieved good agreement (at least 80 percent), with the remainder selected by one reviewer |  |

6. Did the review authors perform data extraction in duplicate?

| Yes | No |
| --- | --- |
| at least two reviewers achieved consensus on which data to extract from included studies | Not reported |
| two reviewers extracted data from a sample of eligible studies and achieved good agreement (at least 80 percent), with the remainder extracted by one reviewer. |  |

7. Did the review authors provide a list of excluded studies and justify the exclusions?

| Yes (Partial Yes plus) | Partial Yes | No |
| --- | --- | --- |
| Justified the exclusion from the review of each potentially relevant study (at least after full-text assessment) | provided a list of all potentially relevant studies that were read in full-text but excluded from the review | Not reported |

8. Did the review authors describe the included studies in adequate detail?

| Yes (Partial Yes + at least 3) | Partial Yes (at least first 4) | No |
| --- | --- | --- |
| described population in detail | described populations | Not reported |
| described intervention in detail (including doses where relevant) | described interventions |  |
| described comparator in detail (including doses where relevant) | described comparators |  |
| described study’s setting | described outcomes |  |
| timeframe for follow-up | described research designs |  |

9. Did the review authors use a satisfactory technique for assessing the risk of bias (RoB) in individual studies that were included in the review?

Adaptation: Only in more recent years, a specific tool (SYRCLE´s) to assess RoB in animal experiments was developed. We applied a more flexible approach and we considered a yes when authors applied alternative approaches to assess RoB

For a long time, no adequate tools or consensus to assess the RoB in animal trials were available. Therefore, we rated the use of a tool developed to assess RoB as *yes.*

| Yes (Partial Yes plus) | Partial Yes | No |
| --- | --- | --- |
| *For RCTs (or adequate tool)* |  | Not reported |
| allocation sequence that was not truly random | Used an adequate tool but did not report the RoB for individual studies only overall |  |
| selection of the reported result from among multiple measurements or analyses of a specified outcome | unconcealed allocation |  |
|  | lack of blinding of patients and assessors when assessing outcomes |  |
| *For NRSIs (or adequate tool)* |  |  |
| methods used to ascertain exposures and outcomes | Used an adequate tool but did not report the RoB for individual studies only overall |  |
| selection of the reported result from among multiple measurements or analyses of a specified outcome | from confounding |  |
|  | from selection bias |  |

10. Did the review authors report on the sources of funding for the studies included in the review?

| Yes | No |
| --- | --- |
| Reported the source of funding of included primary studies | Not reported |
| Authors searched for information on funding but information was not provided in primary studies |  |

11. If meta-analysis was performed did the review authors use appropriate methods for statistical combination of results?

| Yes | No | No MA |
| --- | --- | --- |
| *For RCTs* (at least first 2) | Not reported | Not applicable |
| The authors justified combining the data in a meta-analysis |  |  |
| they used an appropriate weighted technique to combine study results and adjusted for heterogeneity if present. |  |  |
| investigated the causes of any heterogeneity |  |  |
| *For NRSIs* |  |  |
| The authors justified combining the data in a meta-analysis |  |  |
| they used an appropriate weighted technique to combine study results, adjusting for heterogeneity if present |  |  |
| they reported separate summary estimates for RCTs and NRSI separately when both were included in the review |  |  |

12. If meta-analysis was performed, did the review authors assess the potential impact of RoB in individual studies on the results of the meta-analysis or other evidence synthesis?

| Yes (1 of both) | No | No MA |
| --- | --- | --- |
| included only low risk of bias RCTs | Not reported | Not applicable |
| if the pooled estimate was based on RCTs and/or NRSI at variable RoB, the authors performed analyses to investigate possible impact of RoB on summary estimates of effect. |  |  |
| If no MA was conducted, they should comment on the possible impact |  |  |

13. Did the review authors account for RoB in individual studies when interpreting/ discussing the results of the review?

*Adaptation: If the technique of RoB assessment (9.) was inadequate the interpretation and discussion are limited by that. Therefore we rated “No” in those cases.*

| Yes (1 of both) | No |
| --- | --- |
| included only low risk of bias RCTs | Only mentioned |
| if RCTs with moderate or high RoB, or NRSI were included the review provided a discussion of the likely impact of RoB on the results | Did not use a satisfactory method to detect RoB |

14. Did the review authors provide a satisfactory explanation for, and discussion of, any heterogeneity observed in the results of the review?

*Adaptation: Not discussing heterogeneity in full might have different reasons, however mentioning the found heterogeneity is a first step and therefore we added the criteria partial yes. Mentioning this in the context of preclinical research is even more important to find possible differences between animal models.*

| Yes (1 of both) | Partial Yes | No |
| --- | --- | --- |
| There was no significant heterogeneity in the results | Mentioned differences between studies, but did not discuss the impact on the results | Not mentioned |
| if heterogeneity was present the authors performed an investigation (not necessarily statistical test) of sources of any heterogeneity in the results and discussed the impact of this on the results of the review | mentioned that they did not perform meta-analysis due to heterogeneity |  |

15. If they performed quantitative synthesis did the review authors carry out an adequate investigation of publication bias (small study bias) and discuss its likely impact on the results of the review?

| Yes | No | No MA |
| --- | --- | --- |
| performed graphical or statistical tests for publication bias and discussed the likelihood and magnitude of impact of publication bias | Only mentioned |  |
| Planned to perform an investigation on publication bias but were not able to perform due to limited number of studies included |  |  |

16. Did the review authors report any potential sources of conflict of interest, including any funding they received for conducting the review?

| Yes (1 of both) | No |
| --- | --- |
| The authors reported no competing interests | Not reported |
| The authors described their funding sources and how they managed potential conflicts of interest | Conflicting statements |
