# Supplementary material for: Methodological quality of systematic reviews in dentistry including animal studies: a cross-sectional study
Source: Ir Vet J. 2023 Dec 14;76:33. doi: 10.1186/s13620-023-00261-w (PMC10720166; doi:10.1186/s13620-023-00261-w)
Supplement: Supplementary file 3 — Additional file 3. Excluded articles. [file 13620_2023_261_MOESM3_ESM.docx]

Supplementary file 3 – Excluded articles

**Excluded after screening of title and abstract**

No systematic review

Abduo, J., & Judge, R. (2021). Implications of implant framework misfit: An animal study on an ovine model. *Journal of Oral Implantology*, *47*(3), 183–189. Scopus. https://doi.org/10.1563/aaid-joi-D-19-00366

Albrektsson, T., Canullo, L., Cochran, D., & De Bruyn, H. (2016). “Peri-Implantitis”: A Complication of a Foreign Body or a Man-Made “Disease”. Facts and Fiction. *CLINICAL IMPLANT DENTISTRY AND RELATED RESEARCH*, *18*(4), 840–849. https://doi.org/10.1111/cid.12427

Alimohammadi, M., Mirzaee-Rad, S., Feizi, F., Shirzad Juybari, H., Saeidi, A., & Gholinia, H. (2021). Comparison of Rat Connective Tissue Response to BioMTA, Angelus MTA, and Root MTA. *International Journal of Biomaterials*, *2021*. Scopus. https://doi.org/10.1155/2021/7415302

Aljabaa, A. (2020). Clear aligner therapy—Narrative review. *JOURNAL OF INTERNATIONAL ORAL HEALTH*, *12*(7), 1–4. https://doi.org/10.4103/jioh.jioh_180_19

Allen, M. (2011). The effects of bisphosphonates on jaw bone remodeling, tissue properties, and extraction healing. *ODONTOLOGY*, *99*(1), 8–17. https://doi.org/10.1007/s10266-010-0153-0

Al-Maawi, S., Orlowska, A., Sader, R., James Kirkpatrick, C., & Ghanaati, S. (2017). In vivo cellular reactions to different biomaterials—Physiological and pathological aspects and their consequences. *Seminars in Immunology*, *29*, 49–61. Scopus. https://doi.org/10.1016/j.smim.2017.06.001

Amar, S., & Leeman, S. (2013). Periodontal innate immune mechanisms relevant to obesity. *MOLECULAR ORAL MICROBIOLOGY*, *28*(5), 331–341. https://doi.org/10.1111/omi.12035

An, J. Y., Darveau, R., & Kaeberlein, M. (2018). Oral health in geroscience: Animal models and the aging oral cavity. *GeroScience*, *40*(1). Scopus. https://doi.org/10.1007/s11357-017-0004-9

Andreasen, J., & Andersson, L. (2011). Critical considerations when planning experimental in vivo studies in dental traumatology. *DENTAL TRAUMATOLOGY*, *27*(4), 275–280. https://doi.org/10.1111/j.1600-9657.2011.00983.x

Arandi, N., & Rabi, T. (2018). TheraCal LC: From Biochemical and Bioactive Properties to Clinical Applications. *INTERNATIONAL JOURNAL OF DENTISTRY*, *2018*. https://doi.org/10.1155/2018/3484653

Arany, P. (2016). Craniofacial Wound Healing with Photobiomodulation Therapy: New Insights and Current Challenges. *JOURNAL OF DENTAL RESEARCH*, *95*(9), 977–984. https://doi.org/10.1177/0022034516648939

Asa’ad, F., Rasperini, G., Pagni, G., Rios, H., & Gianni, A. (2016). Pre-augmentation soft tissue expansion: An overview. *CLINICAL ORAL IMPLANTS RESEARCH*, *27*(5), 505–522. https://doi.org/10.1111/clr.12617

Atsuta, I., Ayukawa, Y., Kondo, R., Oshiro, W., Matsuura, Y., Furuhashi, A., Tsukiyama, Y., & Koyano, K. (2016). Soft tissue sealing around dental implants based on histological interpretation. *JOURNAL OF PROSTHODONTIC RESEARCH*, *60*(1), 3–11. https://doi.org/10.1016/j.jpor.2015.07.001

Bae, H. W., Patel, V. V., Sardar, Z. M., Badura, J. M., Pradhan, B. B., Seim, H. B., Simon Turner, A., & Toth, J. M. (2016). Transient local bone remodeling effects of rhBMP-2 in an ovine interbody spine fusion model. *Journal of Bone and Joint Surgery - American Volume*, *98*(24), 2061–2070. Scopus. https://doi.org/10.2106/JBJS.16.00345

Bai, B., Zhang, E., Dong, H., & Liu, J. (2015). Biocompatibility of antibacterial Ti–Cu sintered alloy: In vivo bone response. *Journal of Materials Science: Materials in Medicine*, *26*(12). Scopus. https://doi.org/10.1007/s10856-015-5600-6

Bais, M. (2019). Impact of Epigenetic Regulation on Head and Neck Squamous Cell Carcinoma. *JOURNAL OF DENTAL RESEARCH*, *98*(3), 268–276. https://doi.org/10.1177/0022034518816947

Balanta-Melo, J., Toro-Ibacache, V., Kupczik, K., & Buvinic, S. (2019). Mandibular bone loss after masticatory muscles intervention with botulinum toxin: An approach from basic research to clinical findings. *Toxins*, *11*(2). Scopus. https://doi.org/10.3390/toxins11020084

Bannon, K. M. (2013). Clinical Canine Dental Radiography. *Veterinary Clinics of North America - Small Animal Practice*, *43*(3), 507–532. Scopus. https://doi.org/10.1016/j.cvsm.2013.02.011

Bartlett, J., & Smith, C. (2013). Modulation of Cell-Cell Junctional Complexes by Matrix Metalloproteinases. *JOURNAL OF DENTAL RESEARCH*, *92*(1), 10–17. https://doi.org/10.1177/0022034512463397

Beckman, B. (2013). Anesthesia and Pain Management for Small Animals. *Veterinary Clinics of North America - Small Animal Practice*, *43*(3), 669–688. Scopus. https://doi.org/10.1016/j.cvsm.2013.02.006

Bellows, J. (2013). Laser and Radiosurgery in Veterinary Dentistry. *Veterinary Clinics of North America - Small Animal Practice*, *43*(3), 651–668. Scopus. https://doi.org/10.1016/j.cvsm.2013.02.012

Bereiter, D., & Okamoto, K. (2011). NEUROBIOLOGY OF ESTROGEN STATUS IN DEEP CRANIOFACIAL PAIN. In M. Kobayashi, N. Koshikawa, K. Iwata, & J. Waddington (Eds.), *TRANSLATING MECHANISMS OF OROFACIAL NEUROLOGICAL DISORDER* (WOS:000292531100010; Vol. 97, pp. 251–284). https://doi.org/10.1016/B978-0-12-385198-7.00010-2

Bertl, K., Melsen, B., & Stavropoulos, A. (2017). Orthodontic Therapy in Periodontitis Patients—What Should be Taken Care of? *INFORMATIONEN AUS ORTHODONTIE UND KIEFERORTHOPAEDIE*, *49*(1), 11–17.

Blanc-Sylvestre, N., Bouchard, P., Chaussain, C., & Bardet, C. (2021). Pre-clinical models in implant dentistry: Past, present, future. *Biomedicines*, *9*(11). Scopus. https://doi.org/10.3390/biomedicines9111538

Boehlke, C., Zierau, O., & Hannig, C. (2015). Salivary amylase—The enzyme of unspecialized euryphagous animals. *ARCHIVES OF ORAL BIOLOGY*, *60*(8), 1162–1176. https://doi.org/10.1016/j.archoralbio.2015.05.008

Bosshardt, D., Chappuis, V., & Buser, D. (2017). Osseointegration of titanium, titanium alloy and zirconia dental implants: Current knowledge and open questions. *PERIODONTOLOGY 2000*, *73*(1), 22–40. https://doi.org/10.1111/prd.12179

Bourguignon, C., Cohenca, N., Lauridsen, E., Flores, M., O’Connell, A., Day, P., Tsilingaridis, G., Abbott, P., Fouad, A., Hicks, L., Andreasen, J., Cehreli, Z., Harlamb, S., Kahler, B., Oginni, A., Semper, M., & Levin, L. (2020). International Association of Dental Traumatology guidelines for the management of traumatic dental injuries: 1. Fractures and luxations. *DENTAL TRAUMATOLOGY*, *36*(4), 314–330. https://doi.org/10.1111/edt.12578

Brkić, H. (2020). Dental medicine and COVID-19 pandemic. *Acta Stomatologica Croatica*, *54*(2), 118–120. Scopus.

Bronckers, A. (2017). Ion Transport by Ameloblasts during Amelogenesis. *JOURNAL OF DENTAL RESEARCH*, *96*(3), 243–253. https://doi.org/10.1177/0022034516681768

Campillo, V.-E., Langonnet, S., Pierrefeu, A., & Chaux-Bodard, A.-G. (2014). Anatomic and histological study of the rabbit mandible as an experimental model for wound healing and surgical therapies. *Laboratory Animals*, *48*(4), 273–277. Scopus. https://doi.org/10.1177/0023677214540635

Capello, V. (2016a). Diagnostic Imaging of Dental Disease in Pet Rabbits and Rodents. *Veterinary Clinics of North America - Exotic Animal Practice*, *19*(3), 757–782. Scopus. https://doi.org/10.1016/j.cvex.2016.05.001

Capello, V. (2016b). Intraoral Treatment of Dental Disease in Pet Rabbits. *Veterinary Clinics of North America - Exotic Animal Practice*, *19*(3), 783–798. Scopus. https://doi.org/10.1016/j.cvex.2016.05.002

Castejón-González, A. C., & Reiter, A. M. (2022). Oral and Maxillofacial Tumor Management—From Biopsy to Surgical Removal. *Veterinary Clinics of North America - Small Animal Practice*, *52*(1), 235–270. Scopus. https://doi.org/10.1016/j.cvsm.2021.09.005

Castro, D. S., & Guillemot, F. (2011). Old and new functions of proneural factors revealed by the genome-wide characterization of their transcriptional targets. *Cell Cycle*, *10*(23), 4026–4031. Scopus. https://doi.org/10.4161/cc.10.23.18578

Ceusters, W., Nasri-Heir, C., Alnaas, D., Cairns, B., Michelotti, A., & Ohrbach, R. (2015). Perspectives on next steps in classification of oro-facial pain—Part 3: Biomarkers of chronic oro-facial pain—From research to clinic. *JOURNAL OF ORAL REHABILITATION*, *42*(12), 956–966. https://doi.org/10.1111/joor.12324

Chasma, F., Chasma, T., & Irshad, H. (2022). Dental sedation—Airing a concept. *Oral Surgery*. Scopus. https://doi.org/10.1111/ors.12736

Chaves, B. D., Brashears, M. M., & Nightingale, K. K. (2017). Applications and safety considerations of Lactobacillus salivarius as a probiotic in animal and human health. *Journal of Applied Microbiology*, *123*(1), 18–28. Scopus. https://doi.org/10.1111/jam.13438

Chen, C., Zhang, Q., Yu, W., Chang, B., & Le, A. (2020). Oral Mucositis: An Update on Innate Immunity and New Interventional Targets. *JOURNAL OF DENTAL RESEARCH*, *99*(10), 1122–1130. https://doi.org/10.1177/0022034520925421

Chmurska, M., Sowińska, N., & Pietsch-Fulbiszewska, A. (2017). Dental anesthesia in small mammals on the example of domestic rabbits. *Medycyna Weterynaryjna*, *73*(4), 208–213. Scopus. https://doi.org/10.21521/mw.5682

Cho, Y.-D., Kim, S.-J., Bae, H.-S., Yoon, W.-J., Kim, K.-H., Ryoo, H.-M., Seol, Y.-J., Lee, Y.-M., Rhyu, I.-C., & Ku, Y. (2016). Biomimetic approach to stimulate osteogenesis on titanium implant surfaces using fibronectin derived oligopeptide. *Current Pharmaceutical Design*, *22*(30), 4729–4735. Scopus. https://doi.org/10.2174/1381612822666160203143053

Chopra, D., Jayasree, A., Guo, T., Gulati, K., & Ivanovski, S. (2022). Advancing dental implants: Bioactive and therapeutic modifications of zirconia. *Bioactive Materials*, *13*, 161–178. Scopus. https://doi.org/10.1016/j.bioactmat.2021.10.010

Coffman, C. R., & Brigden, G. M. (2013). Oral and Dental Imaging Equipment and Techniques for Small Animals. *Veterinary Clinics of North America - Small Animal Practice*, *43*(3), 489–506. Scopus. https://doi.org/10.1016/j.cvsm.2013.02.007

Coli, P., Christiaens, V., Sennerby, L., & De Bruyn, H. (2017). Reliability of periodontal diagnostic tools for monitoring peri-implant health and disease. *PERIODONTOLOGY 2000*, *73*(1), 203–217. https://doi.org/10.1111/prd.12162

Dadlani, S. (2021). Porcine Acellular Dermal Matrix: An Alternative to Connective Tissue Graft—A Narrative Review. *International Journal of Dentistry*, *2021*. Scopus. https://doi.org/10.1155/2021/1652032

Dam, V. V., Trinh, H. A., Rokaya, D., & Trinh, D. H. (2022). Bone Augmentation for Implant Placement: Recent Advances. *International Journal of Dentistry*, *2022*. Scopus. https://doi.org/10.1155/2022/8900940

Darvell, B., & Wu, R. (2011). “MTA”-An Hydraulic Silicate Cement: Review update and setting reaction. *DENTAL MATERIALS*, *27*(5), 407–422. https://doi.org/10.1016/j.dental.2011.02.001

de la Macorra, J. C., & Pérez-Higueras, J. J. (2014). Microtensile bond strength test bias caused by variations in bonded areas. *The Journal of Adhesive Dentistry*, *16*(3), 207–219. https://doi.org/10.3290/j.jad.a32068

Deng, P., Chen, Q., Hong, C., & Wang, C. (2015). Histone methyltransferases and demethylases: Regulators in balancing osteogenic and adipogenic differentiation of mesenchymal stem cells. *INTERNATIONAL JOURNAL OF ORAL SCIENCE*, *7*(4), 197–204. https://doi.org/10.1038/ijos.2015.41

Di Domenico, M., D’Apuzzo, F., Feola, A., Cito, L., Monsurrò, A., Pierantoni, G. M., Berrino, L., De Rosa, A., Polimeni, A., & Perillo, L. (2012). Cytokines and VEGF induction in orthodontic movement in animal models. *Journal of Biomedicine and Biotechnology*, *2012*. Scopus. https://doi.org/10.1155/2012/201689

Diaz-Sanchez, R., Yanez-Vico, R., Fernandez-Olavarria, A., Mosquera-Perez, R., Iglesias-Linares, A., & Torres-Lagares, D. (2015). Current Approaches of Bone Morphogenetic Proteins in Dentistry. *JOURNAL OF ORAL IMPLANTOLOGY*, *41*(3), 337–342. https://doi.org/10.1563/AAID-JOI-D-13-00012

Dimova, C., Popovska, M., Evrosimovska, B., Zlatanovska, K., Papakoca, K., Petrovski, M., Ivanovska-Stojanoska, M., & Spasovski, S. (2019). Various suturing material and wound healing process after oral surgery procedure—A review paper. *Journal of Hygienic Engineering and Design*, *30*, 95–100. Scopus.

Diniz, M., Gomes, C., de Sousa, S., Xavier, G., & Gomez, R. (2017). Oncogenic signalling pathways in benign odontogenic cysts and tumours. *ORAL ONCOLOGY*, *72*, 165–173. https://doi.org/10.1016/j.oraloncology.2017.07.021

Divya, T., Muddappa, S., Singh, P., Rajan, R., Remya, M., & Sreehari, D. (2021). Drug repurposing for tooth regeneration: The promising premises. *Journal of Pharmacy and Bioallied Sciences*, *13*(6), S957–S959. Scopus. https://doi.org/10.4103/jpbs.jpbs_67_21

Donos, N., Park, J., Vajgel, A., Farias, B., & Dereka, X. (2018). Description of the periodontal pocket in preclinical models: Limitations and considerations. *PERIODONTOLOGY 2000*, *76*(1), 16–34. https://doi.org/10.1111/prd.12155

Duncan, H. F., Smith, A. J., Fleming, G. J. P., & Cooper, P. R. (2016). Epigenetic modulation of dental pulp stem cells: Implications for regenerative endodontics. *International Endodontic Journal*, *49*(5), 431–446. Scopus. https://doi.org/10.1111/iej.12475

Egusa, H. (2012). [IPS cells in dentistry]. *Clinical calcium*, *22*(1), 67–73. Scopus.

Eisner, E. R. (2013). Standard of Care in North American Small Animal Dental Service. *Veterinary Clinics of North America - Small Animal Practice*, *43*(3), 447–469. Scopus. https://doi.org/10.1016/j.cvsm.2013.02.002

Elmanfi, S., Yilmaz, M., Ong, W. W. S., Yeboah, K. S., Sintim, H. O., Gürsoy, M., Könönen, E., & Gürsoy, U. K. (2021). Bacterial cyclic dinucleotides and the cgas–cgamp–sting pathway: A role in periodontitis? *Pathogens*, *10*(6). Scopus. https://doi.org/10.3390/pathogens10060675

Emilia, E., & Neelakantan, P. (2015). Biomarkers in the Dentin-Pulp Complex: Role in Health and Disease. *JOURNAL OF CLINICAL PEDIATRIC DENTISTRY*, *39*(2), 94–99. https://doi.org/10.17796/jcpd.39.2.r32617516412p710

Estrela, C., Decurcio, D., Rossi-Fedele, G., Silva, J., Guedes, O., & Borges, A. (2018). Root perforations: A review of diagnosis, prognosis and materials. *BRAZILIAN ORAL RESEARCH*, *32*, 133–146. https://doi.org/10.1590/1807-3107bor-2018.vol32.0073

Everett, E. (2011). Fluoride’s Effects on the Formation of Teeth and Bones, and the Influence of Genetics. *JOURNAL OF DENTAL RESEARCH*, *90*(5), 552–560. https://doi.org/10.1177/0022034510384626

Eweida, A. M., Horch, R. E., Marei, M. K., Elhammady, H. A., Etaby, A. N., Nabawi, A. S., & Sakr, M. F. (2015). Axially vascularised mandibular constructs: Is it time for a clinical trial? *Journal of Cranio-Maxillo-Facial Surgery : Official Publication of the European Association for Cranio-Maxillo-Facial Surgery*, *43*(7), 1028–1032. https://doi.org/10.1016/j.jcms.2014.10.018

Faggion, C. M. J., Giannakopoulos, N. N., & Listl, S. (2011). Risk of bias of animal studies on regenerative procedures for periodontal and peri-implant bone defects—A systematic review. *Journal of Clinical Periodontology*, *38*(12), 1154–1160. https://doi.org/10.1111/j.1600-051X.2011.01783.x

Fauzi, N., Ardini, Y., Zainuddin, Z., & Lestari, W. (2018). A review on non-syndromic tooth agenesis associated with PAX9 mutations. *JAPANESE DENTAL SCIENCE REVIEW*, *54*(1), 30–36. https://doi.org/10.1016/j.jdsr.2017.08.001

Feigin, K., & Shope, B. (2017). Regenerative endodontics. *Journal of Veterinary Dentistry*, *34*(3), 161–178. Scopus. https://doi.org/10.1177/0898756417722022

Ferracane, J., Cooper, P., & Smith, A. (2010). Can interaction of materials with the dentin-pulp complex contribute to dentin regeneration? *ODONTOLOGY*, *98*(1), 2–14. https://doi.org/10.1007/s10266-009-0116-5

Fleischmannova, J., Matalova, E., Sharpe, P., Misek, I., & Radlanski, R. (2010). Formation of the Tooth-Bone Interface. *JOURNAL OF DENTAL RESEARCH*, *89*(2), 108–115. https://doi.org/10.1177/0022034509355440

Foster, B. (2012). Methods for studying tooth root cementum by light microscopy. *INTERNATIONAL JOURNAL OF ORAL SCIENCE*, *4*(3), 119–128. https://doi.org/10.1038/ijos.2012.57

Fouad, A. (2019). Microbiological aspects of traumatic injuries. *DENTAL TRAUMATOLOGY*, *35*(6), 324–332. https://doi.org/10.1111/edt.12494

Fouad, A., Abbott, P., Tsilingaridis, G., Cohenca, N., Lauridsen, E., Bourguignon, C., O’Connell, A., Flores, M., Day, P., Hicks, L., Andreasen, J., Cehreli, Z., Harlamb, S., Kahler, B., Oginni, A., Semper, M., & Levin, L. (2020). International Association of Dental Traumatology guidelines for the management of traumatic dental injuries: 2. Avulsion of permanent teeth. *DENTAL TRAUMATOLOGY*, *36*(4), 331–342. https://doi.org/10.1111/edt.12573

Francisco, I., Ribeiro, M. P., Marques, F., Travassos, R., Nunes, C., Pereira, F., Caramelo, F., Paula, A. B., & Vale, F. (2022). Application of Three-Dimensional Digital Technology in Orthodontics: The State of the Art. *Biomimetics*, *7*(1). Scopus. https://doi.org/10.3390/biomimetics7010023

Fresia, R., Marangoni, P., Burstyn-Cohen, T., & Sharir, A. (2021). From Bite to Byte: Dental Structures Resolved at a Single-Cell Resolution. *Journal of Dental Research*, *100*(9), 897–905. Scopus. https://doi.org/10.1177/00220345211001848

Fujita, Y., & Maki, K. (2018). Association of feeding behavior with jaw bone metabolism and tongue pressure. *JAPANESE DENTAL SCIENCE REVIEW*, *54*(4), 174–182. https://doi.org/10.1016/j.jdsr.2018.05.001

Fulton, A. J., Fiani, N., Arzi, B., Lommer, M. J., Kuntsi-Vaattovaara, H., & Verstraete, F. J. M. (2012). Outcome of surgical endodontic treatment in dogs: 15 cases (1995-2011). *Journal of the American Veterinary Medical Association*, *241*(12), 1633–1638. Scopus. https://doi.org/10.2460/javma.241.12.1633

Gengler, B. (2013). Exodontics. Extraction of Teeth in the Dog and Cat. *Veterinary Clinics of North America - Small Animal Practice*, *43*(3), 573–585. Scopus. https://doi.org/10.1016/j.cvsm.2013.02.008

Ghoneima, A., Allam, E., Zunt, S., & Windsor, L. (2010). Bisphosphonates treatment and orthodontic considerations. *ORTHODONTICS & CRANIOFACIAL RESEARCH*, *13*(1), 1–10. https://doi.org/10.1111/j.1601-6343.2009.01472.x

Goldstein, G., & Goodacre, C. (2021). Frankfort Mandibular Plane Angle: Critically Appraised Topic (CAT). *Journal of Prosthodontics*, *30*, 61–63. Scopus. https://doi.org/10.1111/jopr.13314

Greenstein, G., & Cavallaro, J. (2010). CRITICAL REVIEW Cantilevers extending from unilateral implant-supported fixed prostheses A review of the literature and presentation of practical guidelines. *JOURNAL OF THE AMERICAN DENTAL ASSOCIATION*, *141*(10), 1221–1230. https://doi.org/10.14219/jada.archive.2010.0049

Gruber, R., Stadlinger, B., & Terheyden, H. (2017). Cell-to-cell communication in guided bone regeneration: Molecular and cellular mechanisms. *CLINICAL ORAL IMPLANTS RESEARCH*, *28*(9), 1139–1146. https://doi.org/10.1111/clr.12929

Guglielmotti, M., Olmedo, D., & Cabrini, R. (2019). Research on implants and osseointegration. *PERIODONTOLOGY 2000*, *79*(1), 178–189. https://doi.org/10.1111/prd.12254

Habelitz, S. (2015). Materials Engineering by Ameloblasts. *JOURNAL OF DENTAL RESEARCH*, *94*(6), 759–767. https://doi.org/10.1177/0022034515577963

Hagandora, C., & Almarza, A. (2012). TMJ Disc Removal: Comparison between Pre-clinical Studies and Clinical Findings. *JOURNAL OF DENTAL RESEARCH*, *91*(8), 745–752. https://doi.org/10.1177/0022034512453324

Hamama, H., Yiu, C., & Burrow, M. (2014). Current update of chemomechanical caries removal methods. *AUSTRALIAN DENTAL JOURNAL*, *59*(4), 446–456. https://doi.org/10.1111/adj.12214

Harunaga, J., Hsu, J., & Yamada, K. (2011). Dynamics of Salivary Gland Morphogenesis. *JOURNAL OF DENTAL RESEARCH*, *90*(9), 1070–1077. https://doi.org/10.1177/0022034511405330

Hasegawa, T., Yamamoto, T., Tsuchiya, E., Hongo, H., Tsuboi, K., Kudo, A., Abe, M., Yoshida, T., Nagai, T., Khadiza, N., Yokoyama, A., Oda, K., Ozawa, H., de Freitas, P., Li, M., & Amizukaa, N. (2017). Ultrastructural and biochemical aspects of matrix vesicle-mediated mineralization. *JAPANESE DENTAL SCIENCE REVIEW*, *53*(2), 34–45. https://doi.org/10.1016/j.jdsr.2016.09.002

Hayakawa, T. (2015). Biochemical surface modifications to titanium implants using the tresyl chloride-activated method. *DENTAL MATERIALS JOURNAL*, *34*(6), 725–739. https://doi.org/10.4012/dmj.2015-067

He, Y., Sui, B., Li, M., Huang, J., Chen, S., & Wu, L. (2016). Site-specific function and regulation of Osterix in tooth root formation. *INTERNATIONAL ENDODONTIC JOURNAL*, *49*(12), 1124–1131. https://doi.org/10.1111/iej.12585

Heath, B., Michmerhuizen, N., Donnelly, C., Sansanaphongpricha, K., Sun, D., Brenner, J., & Lei, Y. (2019). Head and Neck Cancer Immunotherapy beyond the Checkpoint Blockade. *JOURNAL OF DENTAL RESEARCH*, *98*(10), 1073–1080. https://doi.org/10.1177/0022034519864112

Hermann, J. S., Jones, A. A., Bakaeen, L. G., Buser, D., Schoolfield, J. D., & Cochran, D. L. (2011). Influence of a machined collar on crestal bone changes around titanium implants: A histometric study in the canine mandible. *Journal of Periodontology*, *82*(9), 1329–1338. Scopus. https://doi.org/10.1902/jop.2011.090728

Hirate, Y., Yamaguchi, M., & Kasai, K. (2012). Effects of relaxin on relapse and periodontal tissue remodeling after experimental tooth movement in rats. *Connective Tissue Research*, *53*(3), 207–219. Scopus. https://doi.org/10.3109/03008207.2011.628060

Hitomi, S., Ujihara, I., & Ono, K. (2019). Pain mechanism of oral ulcerative mucositis and the therapeutic traditional herbal medicine hangeshashinto. *JOURNAL OF ORAL BIOSCIENCES*, *61*(1), 12–15. https://doi.org/10.1016/j.job.2019.01.004

Holmstrom, S. E. (2012). Veterinary Dentistry in Senior Canines and Felines. *Veterinary Clinics of North America - Small Animal Practice*, *42*(4), 793–808. Scopus. https://doi.org/10.1016/j.cvsm.2012.04.001

Honda, M. J., & Suda, N. (2011). Overview: A new function of amelogenin—From bench to clinics, and clinics to bench—From b. *Journal of Oral Biosciences*, *53*(3), 241–247. Scopus. https://doi.org/10.2330/joralbiosci.53.241

Hossain, M., Shinoda, M., Unno, S., Ando, H., Masuda, Y., Iwata, K., & Kitagawa, J. (2017). Involvement of microglia and astroglia in modulation of the orofacial motor functions in rats with neuropathic pain. *JOURNAL OF ORAL BIOSCIENCES*, *59*(1), 17–22. https://doi.org/10.1016/j.job.2016.11.003

Hovav, A. H., Wilharm, A., Barel, O., & Prinz, I. (2020). Development and Function of γδT Cells in the Oral Mucosa. *Journal of Dental Research*, *99*(5), 498–505. Scopus. https://doi.org/10.1177/0022034520908839

Hovav, A.-H. (2014). Dendritic cells of the oral mucosa. *Mucosal Immunology*, *7*(1), 27–37. Scopus. https://doi.org/10.1038/mi.2013.42

Hsu, J., & Yamada, K. (2010). Salivary Gland Branching Morphogenesis—Recent Progress and Future Opportunities. *INTERNATIONAL JOURNAL OF ORAL SCIENCE*, *2*(3), 117–126. https://doi.org/10.4248/IJOS10042

Iglesias, J., Salum, F., Figueiredo, M., & Cherubini, K. (2015). Important aspects concerning alendronate-related osteonecrosis of the jaws: A literature review. *GERODONTOLOGY*, *32*(3), 169–178. https://doi.org/10.1111/ger.12093

Iglesias-Linares, A., & Hartsfield, J. (2017). Cellular and Molecular Pathways Leading to External Root Resorption. *JOURNAL OF DENTAL RESEARCH*, *96*(2), 145–152. https://doi.org/10.1177/0022034516677539

Ikeda, T., Gion, Y., Nishimura, Y., Nishimura, M. F., Yoshino, T., & Sato, Y. (2021). Epstein-Barr Virus-Positive Mucocutaneous Ulcer: A Unique and Curious Disease Entity. *International Journal of Molecular Sciences*, *22*(3). https://doi.org/10.3390/ijms22031053

Imai, A., & Tsujimura, M. (2017). The small GTPase, Rab27, and its effectors and regulators participate in granule exocytosis by parotid acinar cells. *JOURNAL OF ORAL BIOSCIENCES*, *59*(1), 12–16. https://doi.org/10.1016/j.job.2016.10.003

Imel, E. A., & Peacock, M. (2010). X-linked hypophosphatemia: Understanding and management. *Drugs of the Future*, *35*(9), 755–763. Scopus. https://doi.org/10.1358/dof.2010.035.09.1526651

Inoue, T., Nakayama, K., Ihara, Y., Tachikawa, S., Nakamura, S., Mochizuki, A., Takahashi, K., & Iijima, T. (2017). Coordinated control of the tongue during suckling-like activity and respiration. *JOURNAL OF ORAL SCIENCE*, *59*(2), 183–188. https://doi.org/10.2334/josnusd.16-0850

Ishikawa, M., & Yamada, Y. (2017). The Role of Pannexin 3 in Bone Biology. *JOURNAL OF DENTAL RESEARCH*, *96*(4), 372–379. https://doi.org/10.1177/0022034516678203

Isola, G., Matarese, G., Cordasco, G., Perillo, L., & Ramaglia, L. (2016). Mechanobiology of the tooth movement during the orthodontic treatment: A literature review. *Minerva Stomatologica*, *65*(5), 299–327. Scopus.

Ivanovski, S., & Lee, R. (2018). Comparison of peri-implant and periodontal marginal soft tissues in health and disease. *PERIODONTOLOGY 2000*, *76*(1), 116–130. https://doi.org/10.1111/prd.12150

Iwata, K., Imamura, Y., Honda, K., & Shinoda, M. (2011). PHYSIOLOGICAL MECHANISMS OF NEUROPATHIC PAIN: THE OROFACIAL REGION. In M. Kobayashi, N. Koshikawa, K. Iwata, & J. Waddington (Eds.), *TRANSLATING MECHANISMS OF OROFACIAL NEUROLOGICAL DISORDER* (WOS:000292531100009; Vol. 97, pp. 227–250). https://doi.org/10.1016/B978-0-12-385198-7.00009-6

Iwata, K., Katagiri, A., & Shinoda, M. (2017). Neuron-glia interaction is a key mechanism underlying persistent orofacial pain. *JOURNAL OF ORAL SCIENCE*, *59*(2), 173–175. https://doi.org/10.2334/josnusd.16-0858

Jang, A., Chen, L., Shimotake, A., Landis, W., Altoe, V., Aloni, S., Ryder, M., & Ho, S. (2018). A Force on the Crown and Tug of War in the Periodontal Complex. *JOURNAL OF DENTAL RESEARCH*, *97*(3), 241–250. https://doi.org/10.1177/0022034517744556

Javed, F., Al-Askar, M., & Al-Hezaimi, K. (2012). Cytokine Profile in the Gingival Crevicular Fluid of Periodontitis Patients With and Without Type 2 Diabetes: A Literature Review. *JOURNAL OF PERIODONTOLOGY*, *83*(2), 156–161. https://doi.org/10.1902/jop.2011.110207

Javed, F., Rahman, I., & Romanos, G. E. (2019). Tobacco-product usage as a risk factor for dental implants. *Periodontology 2000*, *81*(1), 48–56. Scopus. https://doi.org/10.1111/prd.12282

Kahler, B., Hu, J., Marriot-Smith, C., & Heithersay, G. (2016). Splinting of teeth following trauma: A review and a new splinting recommendation. *AUSTRALIAN DENTAL JOURNAL*, *61*, 59–73. https://doi.org/10.1111/adj.12398

Kang, J., & Kho, H. (2021). Is a neuropathic mechanism involved in the perception of oral dryness? *ARCHIVES OF ORAL BIOLOGY*, *130*. https://doi.org/10.1016/j.archoralbio.2021.105213

Kashimata, M., & Hayashi, T. (2018). Regulatory mechanisms of branching morphogenesis in mouse submandibular gland rudiments. *JAPANESE DENTAL SCIENCE REVIEW*, *54*(1), 2–7. https://doi.org/10.1016/j.jdsr.2017.06.002

Kawaharada, M., Maruyama, S., Abe, T., Yamazaki, M., Kurokawa, A., Katagiri, W., Takagi, R., Hayashi, T., Kobayashi, T., & Tanuma, J. (2021). Other iatrogenic immunodeficiency-associated lymphoproliferative disorders in the oral cavity: A clinicopathologic study of 4 cases and literature review. *ORAL SURGERY ORAL MEDICINE ORAL PATHOLOGY ORAL RADIOLOGY*, *132*(6), 687–697. https://doi.org/10.1016/j.oooo.2021.05.015

Keinan, D., & Cohen, R. (2013). The Significance of Epithelial Rests of Malassez in the Periodontal Ligament. *JOURNAL OF ENDODONTICS*, *39*(5), 582–587. https://doi.org/10.1016/j.joen.2013.01.004

Ketabi, M., & Deporter, D. (2013). The Effects of Laser Microgrooves on Hard and Soft Tissue Attachment to Implant Collar Surfaces: A Literature Review and Interpretation. *INTERNATIONAL JOURNAL OF PERIODONTICS & RESTORATIVE DENTISTRY*, *33*(6), E145–E152.

Khoo, L., Sakdajeyont, W., Khanijou, M., Seriwatanachai, D., Kiattavorncharoen, S., Pairuchvej, V., & Wongsirichat, N. (2019). Titanium fixture implants treated by laser in dentistry: Review article. *JOURNAL OF ORAL AND MAXILLOFACIAL SURGERY MEDICINE AND PATHOLOGY*, *31*(6), 381–385. https://doi.org/10.1016/j.ajoms.2019.08.001

Kido, M., Yoshimoto, R., Aijima, R., Cao, A., & Gao, W. (2017). The oral mucosal membrane and transient receptor potential channels. *JOURNAL OF ORAL SCIENCE*, *59*(2), 189–193. https://doi.org/10.2334/josnusd.16-0862

Kikuchi, T., Mogi, M., Okabe, I., Okada, K., Goto, H., Sasaki, Y., Fujimura, T., Fukuda, M., & Mitani, A. (2015). Adjunctive application of antimicrobial photodynamic therapy in nonsurgical periodontal treatment: A review of literature. *International Journal of Molecular Sciences*, *16*(10), 24111–24126. Scopus. https://doi.org/10.3390/ijms161024111

Klineberg, I., Trulsson, M., & Murray, G. (2012). Occlusion on implants—Is there a problem? *JOURNAL OF ORAL REHABILITATION*, *39*(7), 522–537. https://doi.org/10.1111/j.1365-2842.2012.02305.x

Kobayashi, M. (2011). MACROSCOPIC CONNECTION OF RAT INSULAR CORTEX: ANATOMICAL BASES UNDERLYING ITS PHYSIOLOGICAL FUNCTIONS. In M. Kobayashi, N. Koshikawa, K. Iwata, & J. Waddington (Eds.), *TRANSLATING MECHANISMS OF OROFACIAL NEUROLOGICAL DISORDER* (WOS:000292531100011; Vol. 97, pp. 285–303). https://doi.org/10.1016/B978-0-12-385198-7.00011-4

Kobayashi, M. (2018). Mechanisms of orofacial sensory processing in the rat insular cortex. *JOURNAL OF ORAL BIOSCIENCES*, *60*(3), 59–64. https://doi.org/10.1016/j.job.2018.04.002

Kobayashi, M., & Horinuki, E. (2017). Neural mechanisms of nociception during orthodontic treatment. *JOURNAL OF ORAL SCIENCE*, *59*(2), 167–171. https://doi.org/10.2334/josnusd.16-0847

Koh, B., Sulaiman, N., Ismadi, S. N. S. W., Ramli, R., Yunus, S. S. M., Idrus, R. B. H., Ariffin, S. H. Z., Wahab, R. M. A., & Yazid, M. D. (2021). Mesenchymal stem cells: A comprehensive methods for odontoblastic induction. *Biological Procedures Online*, *23*(1). Scopus. https://doi.org/10.1186/s12575-021-00155-7

Koshikawa, N., Fujita, S., & Adachi, K. (2011). BEHAVIORAL PHARMACOLOGY OF OROFACIAL MOVEMENT DISORDERS. In M. Kobayashi, N. Koshikawa, K. Iwata, & J. Waddington (Eds.), *TRANSLATING MECHANISMS OF OROFACIAL NEUROLOGICAL DISORDER* (WOS:000292531100001; Vol. 97, pp. 1–38). https://doi.org/10.1016/B978-0-12-385198-7.00001-1

Küchler, E. C., Mazzi-Chaves, J. F., Antunes, L. S., Kirschneck, C., Baratto-Filho, F., & Sousa-Neto, M. D. (2018). Current trends of genetics in apical periodontitis research. *Brazilian Oral Research*, *32*, 126–132. Scopus. https://doi.org/10.1590/1807-3107bor-2018.vol32.0072

Kumar, G., Tewari, S., Tagg, J., Chikindas, M. L., Popov, I. V., & Tiwari, S. K. (2021). Can Probiotics Emerge as Effective Therapeutic Agents in Apical Periodontitis? A Review. *Probiotics and Antimicrobial Proteins*, *13*(2), 299–314. Scopus. https://doi.org/10.1007/s12602-021-09750-2

Kushali, R., Maiti, S., Geetha, R. V., & Jessy. (2020). Animal model used in dentistry-a review. *Indian Journal of Forensic Medicine and Toxicology*, *14*(4), 4534–4543. Scopus. https://doi.org/10.37506/ijfmt.v14i4.12356

Lee, W. (2018). Corticotomy for orthodontic tooth movement. *JOURNAL OF THE KOREAN ASSOCIATION OF ORAL AND MAXILLOFACIAL SURGEONS*, *44*(6), 251–258. https://doi.org/10.5125/jkaoms.2018.44.6.251

Lee, Y.-K., Kim, J.-W., Baek, S.-H., Kim, T.-W., & Chang, Y.-I. (2010). Root and bone response to the proximity of a mini-implant under orthodontic loading. *Angle Orthodontist*, *80*(3), 452–458. Scopus. https://doi.org/10.2319/070209-369.1

Leethanakul, C., Phusuntornsakul, P., & Pravitharangul, A. (2018). Vibratory stimulus and accelerated tooth movement: A critical appraisal. *JOURNAL OF THE WORLD FEDERATION OF ORTHODONTISTS*, *7*(3), 106–112. https://doi.org/10.1016/j.ejwf.2018.07.005

Legendre, L. (2016). Anatomy and Disorders of the Oral Cavity of Guinea Pigs. *Veterinary Clinics of North America - Exotic Animal Practice*, *19*(3), 825–842. Scopus. https://doi.org/10.1016/j.cvex.2016.04.006

Lemmons, M. (2013). Clinical Feline Dental Radiography. *Veterinary Clinics of North America - Small Animal Practice*, *43*(3), 533–554. Scopus. https://doi.org/10.1016/j.cvsm.2013.02.003

Lennox, A. M., & Miwa, Y. (2016). Anatomy and Disorders of the Oral Cavity of Miscellaneous Exotic Companion Mammals. *Veterinary Clinics of North America - Exotic Animal Practice*, *19*(3), 929–945. Scopus. https://doi.org/10.1016/j.cvex.2016.04.005

Levi, B., Brugman, S., Wong, V. W., Grova, M., Longaker, M. T., & Wan, D. C. (2011). Palatogenesis: Engineering, pathways and pathologies. *Organogenesis*, *7*(4), 242–254. Scopus. https://doi.org/10.4161/org.7.4.17926

Lewis, J. R. (2013). Therapeutic Decision Making and Planning in Veterinary Dentistry and Oral Surgery. *Veterinary Clinics of North America - Small Animal Practice*, *43*(3), 471–487. Scopus. https://doi.org/10.1016/j.cvsm.2013.02.009

Li, C., Zhang, X., Zheng, Z., Nguyen, A., Ting, K., & Soo, C. (2019). Nell-1 Is a Key Functional Modulator in Osteochondrogenesis and Beyond. *JOURNAL OF DENTAL RESEARCH*, *98*(13), 1458–1468. https://doi.org/10.1177/0022034519882000

Li, J., Rodriguez, G., Han, X., Janeckova, E., Kahng, S., Song, B., & Chai, Y. (2019). Regulatory Mechanisms of Soft Palate Development and Malformations. *JOURNAL OF DENTAL RESEARCH*, *98*(9), 959–967. https://doi.org/10.1177/0022034519851786

Lin, J., Jang, A., Kurylo, M., Hurng, J., Yang, F., Yang, L., Pal, A., Chen, L., & Ho, S. (2017). Periodontal ligament entheses and their adaptive role in the context of dentoalveolar joint function. *DENTAL MATERIALS*, *33*(6), 650–666. https://doi.org/10.1016/j.dental.2017.03.007

Lin, S., Ashkenazi, M., Karawani, M., Teich, S., & Gutmacher, Z. (2017). Management of Ankylotic Root Resorption Following Dental Trauma: A Short Review and Proposal of a Treatment Protocol. *ORAL HEALTH & PREVENTIVE DENTISTRY*, *15*(5), 467–474. https://doi.org/10.3290/j.ohpd.a38736

Lippert, F., & Hara, A. (2013). Strontium and Caries: A Long and Complicated Relationship. *CARIES RESEARCH*, *47*(1), 34–49. https://doi.org/10.1159/000343008

Liu, Q., Huang, S., Matinlinna, J. P., Chen, Z., & Pan, H. (2013). Insight into biological apatite: Physiochemical properties and preparation approaches. *BioMed Research International*, *2013*. Scopus. https://doi.org/10.1155/2013/929748

Luan, X., Zhou, X., Naqvi, A., Francis, M., Foyle, D., Nares, S., & Diekwisch, T. (2018). MicroRNAs and immunity in periodontal health and disease. *INTERNATIONAL JOURNAL OF ORAL SCIENCE*, *10*. https://doi.org/10.1038/s41368-018-0025-y

Ludwig, K. U., Böhmer, A. C., Bowes, J., Nikolic, M., Ishorst, N., Wyatt, N., Hammond, N. L., Gölz, L., Thieme, F., Barth, S., Schuenke, H., Klamt, J., Spielmann, M., Aldhorae, K., Rojas-Martinez, A., Nöthen, M. M., Rada-Iglesias, A., Dixon, M. J., Knapp, M., & Mangold, E. (2017). Imputation of orofacial clefting data identifies novel risk loci and sheds light on the genetic background of cleft lip ± cleft palate and cleft palate only. *Human Molecular Genetics*, *26*(4), 829–842. https://doi.org/10.1093/hmg/ddx012

MacDonald, K. (2010). Infective endocarditis in dogs: Diagnosis and therapy. *Veterinary Clinics of North America - Small Animal Practice*, *40*(4), 665–684. Scopus. https://doi.org/10.1016/j.cvsm.2010.03.010

Magloire, H., Maurin, J., Couble, M., Shibukawa, Y., Tsumura, M., Thivichon-Prince, B., & Bleicher, F. (2010). Topical Review. Dental Pain and Odontoblasts: Facts and Hypotheses. *JOURNAL OF OROFACIAL PAIN*, *24*(4), 335–349.

Malhotra, N., & Mala, K. (2012). Regenerative endodontics as a tissue engineering approach: Past, current and future. *Australian Endodontic Journal*, *38*(3), 137–148. Scopus. https://doi.org/10.1111/j.1747-4477.2012.00355.x

Manley, L. (2016). On the use of pets to manage dental anxiety. *Dental Hypotheses*, *7*(3), 117–119. Scopus. https://doi.org/10.4103/2155-8213.190518

Manuja, N., Nagpal, R., Pandit, I., & Chaudhary, S. (2010). Dental Pulp Neuropathophysiology. *JOURNAL OF CLINICAL PEDIATRIC DENTISTRY*, *35*(2), 121–127. https://doi.org/10.17796/jcpd.35.2.t13t4834j3567rp5

Mardas, N., Dereka, X., Donos, N., & Dard, M. (2014). Experimental model for bone regeneration in oral and cranio-maxillo-facial surgery. *Journal of Investigative Surgery*, *27*(1), 32–49. Scopus. https://doi.org/10.3109/08941939.2013.817628

Martens, W., Bronckaers, A., Politis, C., Jacobs, R., & Lambrichts, I. (2013). Dental stem cells and their promising role in neural regeneration: An update. *CLINICAL ORAL INVESTIGATIONS*, *17*(9), 1969–1983. https://doi.org/10.1007/s00784-013-1030-3

Martins-Júnior, P. A., Alcântara, C. E., Resende, R. R., & Ferreira, A. J. (2013). Carbon nanotubes: Directions and perspectives in oral regenerative medicine. *Journal of Dental Research*, *92*(7), 575–583. Scopus. https://doi.org/10.1177/0022034513490957

Marton, I., & Kiss, C. (2014). Overlapping Protective and Destructive Regulatory Pathways in Apical Periodontitis. *JOURNAL OF ENDODONTICS*, *40*(2), 155–163. https://doi.org/10.1016/j.joen.2013.10.036

Maruyama, C., Monroe, M., Hunt, J., Buchmann, L., & Baker, O. (2019). Comparing human and mouse salivary glands: A practice guide for salivary researchers. *ORAL DISEASES*, *25*(2), 403–415. https://doi.org/10.1111/odi.12840

Masic, F. (2012). Information systems in dentistry. *Acta Informatica Medica*, *20*(1), 47–55. Scopus. https://doi.org/10.5455/aim.2012.20.47-55

Mickenautsch, S., & Yengopal, V. (2015). Do Laboratory Results Concerning High-Viscosity Glass-Ionomers versus Amalgam for Tooth Restorations Indicate Similar Effect Direction and Magnitude than that of Controlled Clinical Trials? - A Meta-Epidemiological Study. *PloS One*, *10*(7), e0132246. https://doi.org/10.1371/journal.pone.0132246

Miranda, S. C. C. C., Silva, G. A. B., Hell, R. C. R., Martins, M. D., Alves, J. B., & Goes, A. M. (2011). Three-dimensional culture of rat BMMSCs in a porous chitosan-gelatin scaffold: A promising association for bone tissue engineering in oral reconstruction. *Archives of Oral Biology*, *56*(1), 1–15. Scopus. https://doi.org/10.1016/j.archoralbio.2010.08.018

Mitthra, S., Hema, R., Anuradha, B., & Reddy, T. V. K. (2020). Role of silorane composites in dentistry-an overview of the composition and properties. *Indian Journal of Forensic Medicine and Toxicology*, *14*(4), 1179–1183. Scopus. https://doi.org/10.37506/ijfmt.v14i4.11684

Montero-Miralles, P., Martín-González, J., Alonso-Ezpeleta, O., Jiménez-Sánchez, M. C., Velasco-Ortega, E., & Segura-Egea, J. J. (2018). Effectiveness and clinical implications of the use of topical antibiotics in regenerative endodontic procedures: A review. *International Endodontic Journal*, *51*(9), 981–988. Scopus. https://doi.org/10.1111/iej.12913

Moriguchi, K., Utsumi, M., Jogahara, T., Oda, S., & Honda, M. (2017). Structural characterization of endogenous peroxidase activity in human, rat, hamster, and Suncus murinus salivary glands. *JOURNAL OF ORAL BIOSCIENCES*, *59*(4), 184–191. https://doi.org/10.1016/j.job.2017.06.005

Moynihan, P., Makino, Y., Petersen, P., & Ogawa, H. (2018). Implications of WHO Guideline on Sugars for dental health professionals. *COMMUNITY DENTISTRY AND ORAL EPIDEMIOLOGY*, *46*(1), 1–7. https://doi.org/10.1111/cdoe.12353

Murphy, M., MacBarb, R., Wong, M., & Athanasiou, K. (2013). Temporomandibular Disorders: A Review of Etiology, Clinical Management, and Tissue Engineering Strategies. *INTERNATIONAL JOURNAL OF ORAL & MAXILLOFACIAL IMPLANTS*, *28*(6), E393–E414. https://doi.org/10.11607/jomi.te20

Myneni, V., & Mezey, E. (2017). Regulation of bone remodeling by vitamin K2. *ORAL DISEASES*, *23*(8), 1021–1028. https://doi.org/10.1111/odi.12624

Nagata, M., Ono, N., & Ono, W. (2020). Mesenchymal Progenitor Regulation of Tooth Eruption: A View from PTHrP. *JOURNAL OF DENTAL RESEARCH*, *99*(2), 133–142. https://doi.org/10.1177/0022034519882692

Nakamichi, Y., Horibe, K., Takahashi, N., & Udagawa, N. (2014). Roles of cathelicidins in inflammation and bone loss. *ODONTOLOGY*, *102*(2), 137–146. https://doi.org/10.1007/s10266-014-0167-0

Naveh, G., Chattah, N., Zaslansky, P., Shahar, R., & Weiner, S. (2012). Tooth-PDL-bone complex: Response to compressive loads encountered during mastication—A review. *ARCHIVES OF ORAL BIOLOGY*, *57*(12), 1575–1584. https://doi.org/10.1016/j.archoralbio.2012.07.006

Nelson, J., Manzella, K., & Baker, O. (2013). Current cell models for bioengineering a salivary gland: A mini-review of emerging technologies. *ORAL DISEASES*, *19*(3), 236–244. https://doi.org/10.1111/j.1601-0825.2012.01958.x

Nevins, M., Nevins, M. L., Schupbach, P., Kim, S.-W., Lin, Z., & Kim, D. M. (2013). A prospective, randomized controlled preclinical trial to evaluate different formulations of biphasic calcium phosphate in combination with a hydroxyapatite collagen membrane to reconstruct deficient alveolar ridges. *Journal of Oral Implantology*, *39*(2), 133–139. Scopus. https://doi.org/10.1563/AAID-JOI-D-12-00185

Ngeow, W. (2010). Scar less: A review of methods of scar reduction at sites of peripheral nerve repair. *ORAL SURGERY ORAL MEDICINE ORAL PATHOLOGY ORAL RADIOLOGY AND ENDODONTOLOGY*, *109*(3), 357–366. https://doi.org/10.1016/j.tripleo.2009.06.030

Nishikawa, S. (2017). Cytoskeleton, intercellular junctions, planar cell polarity, and cell movement in amelogenesis. *JOURNAL OF ORAL BIOSCIENCES*, *59*(4), 197–204. https://doi.org/10.1016/j.job.2017.07.002

Nosrat, A., Homayounfar, N., & Oloomi, K. (2012). Drawbacks and Unfavorable Outcomes of Regenerative Endodontic Treatments of Necrotic Immature Teeth: A Literature Review and Report of a Case. *JOURNAL OF ENDODONTICS*, *38*(10), 1428–1434. https://doi.org/10.1016/j.joen.2012.06.025

Nuñez-Anita, R. E., Acosta-Torres, L. S., Vilar-Pineda, J., Martínez-Espinosa, J. C., de la Fuente-Hernández, J., & Castaño, V. M. (2014). Toxicology of antimicrobial nanoparticles for prosthetic devices. *International Journal of Nanomedicine*, *9*(1), 3999–4006. Scopus. https://doi.org/10.2147/IJN.S63064

Obuli Ganesh Kishore, S., Don, K. R., & Jothi Priya, A. (2020). Therapeutic potential of stem cells from human exfoliated deciduous teeth(Shed)-a review. *Indian Journal of Forensic Medicine and Toxicology*, *14*(4), 4624–4629. Scopus. https://doi.org/10.37506/ijfmt.v14i4.12367

Ohana, E. (2015). Transepithelial ion transport across duct cells of the salivary gland. *ORAL DISEASES*, *21*(7), 826–835. https://doi.org/10.1111/odi.12201

Okawa, H., Egusa, H., & Nishimura, I. (2020). Implications of the circadian clock in implant dentistry. *Dental Materials Journal*, *39*(2), 173–180. Scopus. https://doi.org/10.4012/dmj.2019-291

Ono, T., & Nakashima, T. (2022). Oral bone biology. *Journal of Oral Biosciences*, *64*(1), 8–17. Scopus. https://doi.org/10.1016/j.job.2022.01.008

Ono, Y., Yamamoto, T., Kubo, K., & Onozuka, M. (2010). Occlusion and brain function: Mastication as a prevention of cognitive dysfunction. *JOURNAL OF ORAL REHABILITATION*, *37*(8), 624–640. https://doi.org/10.1111/j.1365-2842.2010.02079.x

Panzarini, S., Trevisan, C., Brandini, D., Poi, W., Sonoda, C., Luvizuto, E., & dos Santos, C. (2012). Intracanal dressing and root canal filling materials in tooth replantation: A literature review. *DENTAL TRAUMATOLOGY*, *28*(1), 42–48. https://doi.org/10.1111/j.1600-9657.2011.01023.x

Papadimitriou, S. A., & Kouki, M. I. (2016). Teeth and oral cavity diseases in young dogs and cats. *Journal of the Hellenic Veterinary Medical Society*, *67*(3), E.13-E.24. Scopus.

Passarelli, P. C., Saccomanno, S., Angelis, P. D. E., Romeo, A., Piccirillo, G. B., Desantis, V., Grippaudo, C., & D’Addona, A. (2020). Study of cellular toxicity in vitro of two resins for orthodontic use. *European Review for Medical and Pharmacological Sciences*, *24*(2), 930–934. Scopus. https://doi.org/10.26355/eurrev_202001_20078

Pasupuleti, M. K., Molahally, S. S., & Salwaji, S. (2016). Ethical guidelines, animal profile, various animal models used in periodontal research with alternatives and future perspectives. *Journal of Indian Society of Periodontology*, *20*(4), 360–368. Scopus. https://doi.org/10.4103/0972-124X.186931

Pei, Y., Liu, H., Yang, Y., Yang, Y., Jiao, Y., Tay, F. R., & Chen, J. (2018). Biological activities and potential oral applications of N-acetylcysteine: Progress and prospects. *Oxidative Medicine and Cellular Longevity*, *2018*. Scopus. https://doi.org/10.1155/2018/2835787

Pereira, L., Nascimento, J., Rego, J., Canuto, K., Crespo-Lopez, M., Alvarez-Leite, J., Baysan, A., & Oria, R. (2019). Apolipoprotein E, periodontal disease and the risk for atherosclerosis: A review. *ARCHIVES OF ORAL BIOLOGY*, *98*, 204–212. https://doi.org/10.1016/j.archoralbio.2018.11.009

Pescetto, N., Cespedes, A., Molina, R., & Prado, V. (2021). Molecular mechanisms of amelogenesis imperfecta. A review of the ENAM, AMBN, FAM83H, MMP20, and KLK4 genes. *ODONTOESTOMATOLOGIA*, *23*(38). https://doi.org/10.22592/ode2021n37e306

Pitak-Arnnop, P., Hemprich, A., Dhanuthai, K., & Pausch, N. (2013). Fibular flap for mandibular reconstruction: Are there old tricks for an old dog? *REVUE DE STOMATOLOGIE DE CHIRURGIE MAXILLO-FACIALE ET DE CHIRURGIE ORALE*, *114*(1), 15–18. https://doi.org/10.1016/j.stomax.2012.05.001

Pitak-Arnnop, P., Schubert, S., Dhanuthai, K., Sappayatosok, K., Bauer, U., Ngamwannagul, P., Liebert, U., & Hemprich, A. (2010). Swine-origin H1N1 influenza A virus and dental practice: A critical review. *CLINICAL ORAL INVESTIGATIONS*, *14*(1), 11–17. https://doi.org/10.1007/s00784-009-0373-2

Pradhan, S. R., Singh, R., & Banwait, S. S. (2021). On crown fabrication in prosthetic dentistry of veterinary patients: A review. *Advances in Materials and Processing Technologies*. Scopus. https://doi.org/10.1080/2374068X.2021.1970991

Quade, B. N., Parker, M. D., & Occhipinti, R. (2021). The therapeutic importance of acid-base balance. *Biochemical Pharmacology*, *183*. Scopus. https://doi.org/10.1016/j.bcp.2020.114278

Rajaji, D., Sunil, E., Mukunda, A., Pynadath, M., Mohan, A., & Samuel, S. (2018). Emerging and Re-emerging Infections. *ORAL & MAXILLOFACIAL PATHOLOGY JOURNAL*, *9*(2), 76–78. https://doi.org/10.5005/jp-journals-10037-1134

Raju, B., & Ibrahim, S. (2011). Pathophysiology of oral cancer in experimental animal models: A review with focus on the role of sympathetic nerves. *JOURNAL OF ORAL PATHOLOGY & MEDICINE*, *40*(1), 1–9. https://doi.org/10.1111/j.1600-0714.2010.00928.x

Raskó, Z., Nagy, L., Radnai, M., Piffkó, J., & Baráth, Z. (2016). Assessing the accuracy of cone-beam computerized tomography in measuring thinning oral and buccal bone. *Journal of Oral Implantology*, *42*(3), 311–314. Scopus. https://doi.org/10.1563/aaid-joi-D-15-00188

Reiter, A. M. (2013). Equipment for Oral Surgery in Small Animals. *Veterinary Clinics of North America - Small Animal Practice*, *43*(3), 587–608. Scopus. https://doi.org/10.1016/j.cvsm.2013.02.005

Retzepi, M., & Donos, N. (2010). The effect of diabetes mellitus on osseous healing. *CLINICAL ORAL IMPLANTS RESEARCH*, *21*(7), 673–681. https://doi.org/10.1111/j.1600-0501.2010.01923.x

Ritchie, H. (2018). The functional significance of dentin sialoprotein-phosphophoryn and dentin sialoprotein. *INTERNATIONAL JOURNAL OF ORAL SCIENCE*, *10*. https://doi.org/10.1038/s41368-018-0035-9

Rodriguez-Caballero, A., Torres-Lagares, D., Rodriguez-Perez, A., Serrera-Figallo, M., Hernandez-Guisado, J., & Machuca-Portillo, G. (2010). Cri du chat syndrome: A critical review. *MEDICINA ORAL PATOLOGIA ORAL Y CIRUGIA BUCAL*, *15*(3), E473–E478. https://doi.org/10.4317/medoral.15.e473

Rodriguez-Lozano, F., Bueno, C., Insausti, C., Meseguer, L., Ramirez, M., Blanquer, M., Marin, N., Martinez, S., & Moraleda, J. (2011). Mesenchymal stem cells derived from dental tissues. *INTERNATIONAL ENDODONTIC JOURNAL*, *44*(9), 800–806. https://doi.org/10.1111/j.1365-2591.2011.01877.x

Roelen, B. A. J., & Chuva De Sousa Lopes, S. M. (2011). Origins of pluripotent stem cells. *Minerva Ginecologica*, *63*(4), 351–363. Scopus.

Romano, R., Solomon, L., & Sinha, S. (2012). Tp63 in Oral Development, Neoplasia, and Autoimmunity. *JOURNAL OF DENTAL RESEARCH*, *91*(2), 125–132. https://doi.org/10.1177/0022034511411302

Sahoo, S., Goel, M., Gandhi, P., & Saxena, S. (2013). Biological aspects of dental implant; Current knowledge and perspectives in oral implantology. *Dental Hypotheses*, *4*(3), 87–91. Scopus. https://doi.org/10.4103/2155-8213.116336

Saito, K., Chiba, Y., Yamada, A., & Fukumoto, S. (2020). Identification and function analysis of ameloblast differentiation-related molecules using mouse incisors. *Pediatric Dental Journal*, *30*(3), 129–138. Scopus. https://doi.org/10.1016/j.pdj.2020.08.001

Saito, K., & Ohshima, H. (2017). Differentiation capacity and maintenance of dental pulp stem/progenitor cells in the process of pulpal healing following tooth injuries. *JOURNAL OF ORAL BIOSCIENCES*, *59*(2), 63–70. https://doi.org/10.1016/j.job.2017.03.001

Saruta, J., To, M., Sakaguchi, W., Kondo, Y., & Tsukinoki, K. (2020). Brain-derived neurotrophic factor is related to stress and chewing in saliva and salivary glands. *JAPANESE DENTAL SCIENCE REVIEW*, *56*(1), 43–49. https://doi.org/10.1016/j.jdsr.2019.11.001

Sasano, Y., Nakamura, M., Henmi, A., Okata, H., Suzuki, O., Kayaba, A., & Mayanagi, M. (2019). Degradation of extracellular matrices propagates calcification during development and healing in bones and teeth. *JOURNAL OF ORAL BIOSCIENCES*, *61*(3), 149–156. https://doi.org/10.1016/j.job.2019.07.004

Sato, T., & Ishii, H. (2017). Regulation of hemodynamics in major salivary glands by parasympathetic vasodilation. *JOURNAL OF ORAL BIOSCIENCES*, *59*(2), 80–86. https://doi.org/10.1016/j.job.2017.03.002

Sawan, N. M. (2021). Clear Aligners in Patients with Amelogenesis and Dentinogenesis Imperfecta. *International Journal of Dentistry*, *2021*. Scopus. https://doi.org/10.1155/2021/7343094

Schweigel, H., Wicht, M., & Schwendicke, F. (2016). Salivary and pellicle proteome: A datamining analysis. *Scientific Reports*, *6*, 38882. https://doi.org/10.1038/srep38882

Sciote, J., Raoul, G., Ferri, J., Close, J., Horton, M., & Rowlerson, A. (2013). Masseter function and skeletal malocclusion. *REVUE DE STOMATOLOGIE ET DE CHIRURGIE MAXILLO-FACIALE*, *114*(2), 79–85. https://doi.org/10.1016/j.revsto.2013.01.015

Scott, M. A., Nguyen, V. T., Levi, B., & James, A. W. (2011). Current methods of adipogenic differentiation of mesenchymal stem cells. *Stem Cells and Development*, *20*(10), 1793–1804. https://doi.org/10.1089/scd.2011.0040

Seol, S., & Chung, G. (2022). Review article Estrogen-dependent regulation of transient receptor potential vanilloid 1 (TRPV1) and P2X purinoceptor 3 (P2X3): Implication in burning mouth syndrome. *JOURNAL OF DENTAL SCIENCES*, *17*(1), 8–13. https://doi.org/10.1016/j.jds.2021.06.007

Sharma, D., Hamlet, S., Petcu, E., & Ivanovski, S. (2013). Animal models for bisphosphonate-related osteonecrosis of the jaws—An appraisal. *ORAL DISEASES*, *19*(8), 747–754. https://doi.org/10.1111/odi.12067

Sharma, R. (2016). iPS cells—The triumphs and tribulations. *Dentistry Journal*, *4*(2). Scopus. https://doi.org/10.3390/dj4020019

Sicilia, A., & Botticelli, D. (2012). Computer-guided implant therapy and soft- and hard-tissue aspects. The Third EAO Consensus Conference 2012. *Clinical Oral Implants Research*, *23 Suppl 6*, 157–161. https://doi.org/10.1111/j.1600-0501.2012.02553.x

Simmer, J., Papagerakis, P., Smith, C., Fisher, D., Rountrey, A., Zheng, L., & Hu, J. (2010). Regulation of Dental Enamel Shape and Hardness. *JOURNAL OF DENTAL RESEARCH*, *89*(10), 1024–1038. https://doi.org/10.1177/0022034510375829

Simmer, J., Richardson, A., Hu, Y., Smith, C., & Hu, J. (2012). A post-classical theory of enamel biomineralization ... And why we need one. *INTERNATIONAL JOURNAL OF ORAL SCIENCE*, *4*(3), 129–134. https://doi.org/10.1038/ijos.2012.59

Singh, P. (2011). Understanding Peri-implantitis: A Strategic Review. *JOURNAL OF ORAL IMPLANTOLOGY*, *37*(5), 622–626. https://doi.org/10.1563/AAID-JOI-D-10-00134

Sisti, A., Canullo, L., Mottola, M., Covani, U., Barone, A., & Botticelli, D. (2012). Clinical evaluation of a ridge augmentation procedure for the severely resorbed alveolar socket: Multicenter randomized controlled trial, preliminary results. *CLINICAL ORAL IMPLANTS RESEARCH*, *23*(5), 526–535. https://doi.org/10.1111/j.1600-0501.2011.02386.x

Smith, S. B., Parisien, M., Bair, E., Belfer, I., Chabot-Doré, A.-J., Gris, P., Khoury, S., Tansley, S., Torosyan, Y., Zaykin, D. V., Bernhardt, O., de Oliveira Serrano, P., Gracely, R. H., Jain, D., Järvelin, M.-R., Kaste, L. M., Kerr, K. F., Kocher, T., Lähdesmäki, R., … Diatchenko, L. (2019). Genome-wide association reveals contribution of MRAS to painful temporomandibular disorder in males. *Pain*, *160*(3), 579–591. https://doi.org/10.1097/j.pain.0000000000001438

Song, L., Dong, G., Guo, L., & Graves, D. (2018). The function of dendritic cells in modulating the host response. *MOLECULAR ORAL MICROBIOLOGY*, *33*(1), 13–21. https://doi.org/10.1111/omi.12195

Sood, S., Gupta, S., & Mahendra, A. (2012). Gene therapy with growth factors for periodontal tissue engineering-A review. *MEDICINA ORAL PATOLOGIA ORAL Y CIRUGIA BUCAL*, *17*(2), E301–E310. https://doi.org/10.4317/medoral.17472

Staubli, N., Schmidt, J. C., Rinne, C. A., Signer-Buset, S. L., Rodriguez, F. R., & Walter, C. (2019). Animal experiments in periodontal and peri-implant research: Are there any changes? *Dentistry Journal*, *7*(2). Scopus. https://doi.org/10.3390/dj7020046

Steindorff, M., Lehl, H., Winkel, A., & Stiesch, M. (2014). Innovative approaches to regenerate teeth by tissue engineering. *ARCHIVES OF ORAL BIOLOGY*, *59*(2), 158–166. https://doi.org/10.1016/j.archoralbio.2013.11.005

Strydom, H., Maltha, J., Kuijpers-Jagtman, A., & Von den Hoff, J. (2012). The oxytalan fibre network in the periodontium and its possible mechanical function. *ARCHIVES OF ORAL BIOLOGY*, *57*(8), 1003–1011. https://doi.org/10.1016/j.archoralbio.2012.06.003

Stübinger, S., & Dard, M. (2013). The Rabbit as Experimental Model for Research in Implant Dentistry and Related Tissue Regeneration. *Journal of Investigative Surgery*, *26*(5), 266–282. Scopus. https://doi.org/10.3109/08941939.2013.778922

Su, Y., Cockerill, I., Zheng, Y., Tang, L., Qin, Y.-X., & Zhu, D. (2019). Biofunctionalization of metallic implants by calcium phosphate coatings. *Bioactive Materials*, *4*, 196–206. Scopus. https://doi.org/10.1016/j.bioactmat.2019.05.001

Sumit, B., & Geetika, A. (2012). Therapeutic benefits of holy basil (TULSI) in general and oral medicine: A review. *International Journal of Research in Ayurveda and Pharmacy*, *3*(6), 761–764. Scopus. https://doi.org/10.7897/2277-4343.03611

Suzuki, A., & Iwata, J. (2016). Mouse genetic models for temporomandibular joint development and disorders. *ORAL DISEASES*, *22*(1), 33–38. https://doi.org/10.1111/odi.12353

Tatara, A., Wong, M., & Mikos, A. (2014). In Vivo Bioreactors for Mandibular Reconstruction. *JOURNAL OF DENTAL RESEARCH*, *93*(12), 1196–1202. https://doi.org/10.1177/0022034514547763

Tatullo, M., Marrelli, M., Shakesheff, K. M., & White, L. J. (2015). Dental pulp stem cells: Function, isolation and applications in regenerative medicine. *Journal of Tissue Engineering and Regenerative Medicine*, *9*(11), 1205–1216. Scopus. https://doi.org/10.1002/term.1899

Thennavan, A., Sharma, M., Chandrashekar, C., Hunter, K., & Radhakrishnan, R. (2017). Exploring the potential of laser capture microdissection technology in integrated oral biosciences. *ORAL DISEASES*, *23*(6), 737–748. https://doi.org/10.1111/odi.12578

Toan, N. K., & Ahn, S.-G. (2021). Aging-Related Metabolic Dysfunction in the Salivary Gland: A Review of the Literature. *International Journal of Molecular Sciences*, *22*(11). https://doi.org/10.3390/ijms22115835

Tomiyama, K., O’Tuathaigh, C., & Waddington, J. (2011). REGULATION OF OROFACIAL MOVEMENT: AMINO ACID MECHANISMS AND MUTANT MODELS. In M. Kobayashi, N. Koshikawa, K. Iwata, & J. Waddington (Eds.), *TRANSLATING MECHANISMS OF OROFACIAL NEUROLOGICAL DISORDER* (WOS:000292531100003; Vol. 97, pp. 61–75). https://doi.org/10.1016/B978-0-12-385198-7.00003-5

Torabinejad, M., & Parirokh, M. (2010). Mineral Trioxide Aggregate: A Comprehensive Literature Review-Part II: Leakage and Biocompatibility Investigations. *JOURNAL OF ENDODONTICS*, *36*(2), 190–202. https://doi.org/10.1016/j.joen.2009.09.010

Trope, M. (2011). Avulsion of permanent teeth: Theory to practice. *DENTAL TRAUMATOLOGY*, *27*(4), 281–294. https://doi.org/10.1111/j.1600-9657.2011.01003.x

Tsai, M. H., Megat Abdul Wahab, R., & Yazid, F. (2021). Timing of orthodontic tooth movement in bone defects repaired with synthetic scaffolds: A scoping review of animal studies. *Archives of Oral Biology*, *132*, 105278. https://doi.org/10.1016/j.archoralbio.2021.105278

Tsukuba, T., Sakai, E., Nishishita, K., Kadowaki, T., & Okamoto, K. (2017). New functions of lysosomes in bone cells. *JOURNAL OF ORAL BIOSCIENCES*, *59*(2), 92–95. https://doi.org/10.1016/j.job.2017.01.004

Uehara, S., Udagawa, N., & Kobayashi, Y. (2019). Regulation of osteoclast function via Rho-Pkn3-c-Src pathways. *JOURNAL OF ORAL BIOSCIENCES*, *61*(3), 135–140. https://doi.org/10.1016/j.job.2019.07.002

Vali, S., Khosravani, S., Nobar, B. R., & Motamedian, S. R. (2022). Rapid maxillary expansion supplementary methods: A scoping review of animal studies. *International Orthodontics*, *20*(1), 100614. https://doi.org/10.1016/j.ortho.2022.100614

Varma, S., AlShayeb, M., Narayanan, J., Abuhijleh, E., Hadi, A., Jaber, M., & Abu Fanas, S. (2020). Applications of Lasers in Refractory Periodontitis: A Narrative Review. *JOURNAL OF INTERNATIONAL SOCIETY OF PREVENTIVE AND COMMUNITY DENTISTRY*, *10*(4), 384–393. https://doi.org/10.4103/jispcd.JISPCD_241_20

Vaseenon, S., Chattipakorn, N., & Chattipakorn, S. (2021). Effects of melatonin in wound healing of dental pulp and periodontium: Evidence from in vitro, in vivo and clinical studies. *ARCHIVES OF ORAL BIOLOGY*, *123*. https://doi.org/10.1016/j.archoralbio.2020.105037

Velliyagounder, K., Bahdila, D., Pawar, S., & Fine, D. (2019). Role of lactoferrin and lactoferrin-derived peptides in oral and maxillofacial diseases. *ORAL DISEASES*, *25*(3), 652–669. https://doi.org/10.1111/odi.12868

Viet, C., & Schmidt, B. (2012). Biologic Mechanisms of Oral Cancer Pain and Implications for Clinical Therapy. *JOURNAL OF DENTAL RESEARCH*, *91*(5), 447–453. https://doi.org/10.1177/0022034511424156

Vignoletti, F., & Abrahamsson, I. (2012). Quality of reporting of experimental research in implant dentistry. Critical aspects in design, outcome assessment and model validation. *JOURNAL OF CLINICAL PERIODONTOLOGY*, *39*, 6–27. https://doi.org/10.1111/j.1600-051X.2011.01830.x

Wang, J., & Feng, J. (2017). Signaling Pathways Critical for Tooth Root Formation. *JOURNAL OF DENTAL RESEARCH*, *96*(11), 1221–1228. https://doi.org/10.1177/0022034517717478

Wang, X.-P. (2013). Tooth eruption without roots. *Journal of Dental Research*, *92*(3), 212–214. Scopus. https://doi.org/10.1177/0022034512474469

Weeden, A. M., & Degner, D. A. (2016). Surgical Approaches to the Nasal Cavity and Sinuses. *Veterinary Clinics of North America - Small Animal Practice*, *46*(4), 719–733. Scopus. https://doi.org/10.1016/j.cvsm.2016.02.004

Wen, X., Yi, L.-Z., Liu, F., Wei, J.-H., & Xue, Y. (2016). The role of cathepsin K in oral and maxillofacial disorders. *Oral Diseases*, *22*(2), 109–115. Scopus. https://doi.org/10.1111/odi.12378

Werneck, R., Mira, M., & Trevilatto, P. (2010). A critical review: An overview of genetic influence on dental caries. *ORAL DISEASES*, *16*(7), 613–623. https://doi.org/10.1111/j.1601-0825.2010.01675.x

Westberg, K., & Kolta, A. (2011). THE TRIGEMINAL CIRCUITS RESPONSIBLE FOR CHEWING. In M. Kobayashi, N. Koshikawa, K. Iwata, & J. Waddington (Eds.), *TRANSLATING MECHANISMS OF OROFACIAL NEUROLOGICAL DISORDER* (WOS:000292531100004; Vol. 97, pp. 77–98). https://doi.org/10.1016/B978-0-12-385198-7.00004-7

Whitlock, J., & Richman, J. (2013). Biology of tooth replacement in amniotes. *INTERNATIONAL JOURNAL OF ORAL SCIENCE*, *5*(2), 66–70. https://doi.org/10.1038/ijos.2013.36

Wilensky, A., Segev, H., Mizraji, G., Shaul, Y., Capucha, T., Shacham, M., & Hovav, A. (2014). Dendritic cells and their role in periodontal disease. *ORAL DISEASES*, *20*(2), 119–126. https://doi.org/10.1111/odi.12122

Wilson, C., Martin-Saavedra, F., Vilaboa, N., & Franceschi, R. (2013). Advanced BMP Gene Therapies for Temporal and Spatial Control of Bone Regeneration. *JOURNAL OF DENTAL RESEARCH*, *92*(5), 409–417. https://doi.org/10.1177/0022034513483771

Wojda, S. J., & Donahue, S. W. (2018). Parathyroid hormone for bone regeneration. *Journal of Orthopaedic Research*, *36*(10), 2586–2594. Scopus. https://doi.org/10.1002/jor.24075

Yamakoshi, Y., & Simmer, J. (2018). Structural features, processing mechanism and gene splice variants of dentin sialophosphoprotein. *JAPANESE DENTAL SCIENCE REVIEW*, *54*(4), 183–196. https://doi.org/10.1016/j.jdsr.2018.03.006

Yamamoto, T., Hasegawa, T., Hongo, H., & Amizuka, N. (2019). Alternating lamellar structure in human cellular cementum and rat compact bone: Its structure and formation. *JOURNAL OF ORAL BIOSCIENCES*, *61*(2), 105–114. https://doi.org/10.1016/j.job.2019.03.006

Yassir, Y., Nabbat, S., McIntyre, G., & Bearn, D. (2022). Clinical effectiveness of clear aligner treatment compared to fixed appliance treatment: An overview of systematic reviews. *CLINICAL ORAL INVESTIGATIONS*, *26*(3), 2353–2370. https://doi.org/10.1007/s00784-021-04361-1

Yeung, A. W. K., Tzvetkov, N. T., Georgieva, M. G., Ognyanov, I. V., Kordos, K., Jóźwik, A., Kühl, T., Perry, G., Petralia, M. C., Mazzon, E., & Atanasov, A. G. (2021). Reactive Oxygen Species and Their Impact in Neurodegenerative Diseases: Literature Landscape Analysis. *Antioxidants & Redox Signaling*, *34*(5), 402–420. https://doi.org/10.1089/ars.2019.7952

Yin, X., Li, J., Salmon, B., Huang, L., Lim, W., Liu, B., Hunter, D., Ransom, R., Singh, G., Gillette, M., Zou, S., & Helms, J. (2015). Wnt Signaling and Its Contribution to Craniofacial Tissue Homeostasis. *JOURNAL OF DENTAL RESEARCH*, *94*(11), 1487–1494. https://doi.org/10.1177/0022034515599772

Yoshizawa, M., Koyama, T., Izumi, N., Niimi, K., Ono, Y., Ajima, H., Funayama, A., Mikami, T., Kobayashi, T., Ono, K., Takagi, R., & Saito, C. (2014). Autotransplantation or replantation of cryopreserved teeth: A case series and literature review. *DENTAL TRAUMATOLOGY*, *30*(1), 71–75. https://doi.org/10.1111/edt.12039

Yu, C., & Abbott, P. (2018). Pulp microenvironment and mechanisms of pain arising from the dental pulp: From an endodontic perspective. *AUSTRALIAN ENDODONTIC JOURNAL*, *44*(2), 82–98. https://doi.org/10.1111/aej.12257

Yu, N., Yang, J., Mishina, Y., & Giannobile, W. (2019). Genome Editing: A New Horizon for Oral and Craniofacial Research. *JOURNAL OF DENTAL RESEARCH*, *98*(1), 36–45. https://doi.org/10.1177/0022034518805978

Yuan, X., & Yang, S. (2016). Primary Cilia and Intraflagellar Transport Proteins in Bone and Cartilage. *JOURNAL OF DENTAL RESEARCH*, *95*(12), 1341–1349. https://doi.org/10.1177/0022034516652383

Zacher, A. M., & Marretta, S. M. (2013). Oral and Maxillofacial Surgery in Dogs and Cats. *Veterinary Clinics of North America - Small Animal Practice*, *43*(3), 609–649. Scopus. https://doi.org/10.1016/j.cvsm.2013.02.010

Zacher, A., & Manfra Marretta, S. (2021). Decision-Making and Management of Immature Permanent Teeth with Crown Fractures in Small Animals—A Review. *Journal of Veterinary Dentistry*, *38*(2), 81–92. Scopus. https://doi.org/10.1177/08987564211046325

Zhang, X., Zara, J., Siu, R., Ting, K., & Soo, C. (2010). The Role of NELL-1, a Growth Factor Associated with Craniosynostosis, in Promoting Bone Regeneration. *JOURNAL OF DENTAL RESEARCH*, *89*(9), 865–878. https://doi.org/10.1177/0022034510376401

Zhao, N., Foster, B., & Bonewald, L. (2016). The Cementocyte-An Osteocyte Relative? *JOURNAL OF DENTAL RESEARCH*, *95*(7), 734–741. https://doi.org/10.1177/0022034516641898

No animals included

Adnan, S., Lone, M. M., Khan, F. R., Hussain, S. M., & Nagi, S. E. (2018). Which is the most recommended medium for the storage and transport of avulsed teeth? A systematic review. *Dental Traumatology : Official Publication of International Association for Dental Traumatology*, *34*(2), 59–70. https://doi.org/10.1111/edt.12382

Alakhali, M. S., Al-Maweri, S. A., Al-Shamiri, H. M., Al-Haddad, K., & Halboub, E. (2018). The potential association between periodontitis and non-alcoholic fatty liver disease: A systematic review. *Clinical Oral Investigations*, *22*(9), 2965–2974. https://doi.org/10.1007/s00784-018-2726-1

Al-Maweri, S. A., Ibraheem, W. I., Al-Ak’hali, M. S., Shamala, A., Halboub, E., & Alhajj, M. N. (2021). Association of periodontitis and tooth loss with liver cancer: A systematic review. *Critical Reviews in Oncology/Hematology*, *159*, 103221. https://doi.org/10.1016/j.critrevonc.2021.103221

Alves, K., Franco, K., Sassaki, K., Buzalaf, M., & Delbem, A. (2011). Effect of iron on enamel demineralization and remineralization in vitro. *ARCHIVES OF ORAL BIOLOGY*, *56*(11), 1192–1198. https://doi.org/10.1016/j.archoralbio.2011.04.011

Amerio, E., Mainas, G., Petrova, D., Tarrida, L., Nart, J., & Monje, A. (2020). Compliance with supportive periodontal/peri-implant therapy: A systematic review. *JOURNAL OF CLINICAL PERIODONTOLOGY*, *47*(1), 81–100. https://doi.org/10.1111/jcpe.13204

Amid, R., Kheiri, A., Kheiri, L., Kadkhodazadeh, M., & Ekhlasmandkermani, M. (2021). Structural and chemical features of xenograft bone substitutes: A systematic review of in vitro studies. *Biotechnology and Applied Biochemistry*, *68*(6), 1432–1452. https://doi.org/10.1002/bab.2065

Anna, D., Dimitra, S., & Kleoniki, L. (2020). Histological assessment of human regenerative endodontic procedures (REP) of immature permanent teeth with necrotic pulp/apical periodontitis: A systematic review. *AUSTRALIAN ENDODONTIC JOURNAL*, *46*(1), 140–153. https://doi.org/10.1111/aej.12371

Arqub, S. A., Gandhi, V., Iverson, M. G., Ahmed, M., Kuo, C.-L., Mu, J., Dutra, E., & Uribe, F. (2021). The effect of the local administration of biological substances on the rate of orthodontic tooth movement: A systematic review of human studies. *Progress in Orthodontics*, *22*(1), 5. https://doi.org/10.1186/s40510-021-00349-5

Arun, D., Adikari Mudiyanselage, D., Gulam Mohamed, R., Liddell, M., Monsur Hassan, N. M., & Sharma, D. (2021). Does the addition of zinc oxide nanoparticles improve the antibacterial properties of direct dental composite resins? A systematic review. *Materials*, *14*(1), 1–15. Scopus. https://doi.org/10.3390/ma14010040

Avila-Ortiz, G., Chambrone, L., & Vignoletti, F. (2019). Effect of alveolar ridge preservation interventions following tooth extraction: A systematic review and meta-analysis. *Journal of Clinical Periodontology*, *46 Suppl 21*, 195–223. https://doi.org/10.1111/jcpe.13057

Badran, Z., Struillou, X., Strube, N., Bourdin, D., Dard, M., Soueidan, A., & Hoornaert, A. (2017). Clinical Performance of Narrow-Diameter Titanium-Zirconium Implants: A Systematic Review. *Implant Dentistry*, *26*(2), 316–323. https://doi.org/10.1097/ID.0000000000000557

Chaves, C. de A. L., Machado, A. L., Vergani, C. E., de Souza, R. F., & Giampaolo, E. T. (2012). Cytotoxicity of denture base and hard chairside reline materials: A systematic review. *The Journal of Prosthetic Dentistry*, *107*(2), 114–127. https://doi.org/10.1016/S0022-3913(12)60037-7

Chaware, S. H., Thakare, V., Chaudhary, R., Jankar, A., Thakkar, S., & Borse, S. (2021). The rehabilitation of posterior atrophic maxilla by using the graftless option of short implant versus conventional long implant with sinus graft: A systematic review and meta-analysis of randomized controlled clinical trial. *Journal of Indian Prosthodontic Society*, *21*(1), 28–44. https://doi.org/10.4103/jips.jips_400_20

Chrcanovic, B., Guimara, L., Gomes, C., & Gomez, R. (2021). Cherubism: A systematic literature review of clinical and molecular aspects. *INTERNATIONAL JOURNAL OF ORAL AND MAXILLOFACIAL SURGERY*, *50*(1), 43–53. https://doi.org/10.1016/j.ijom.2020.05.021

Coelho Dos Santos, D., Silva Barboza, A. da, Ribeiro, J. S., Rodrigues Junior, S. A., Campos, Â. D., & Lund, R. G. (2022). Bixa orellana L. (Achiote, Annatto) as an antimicrobial agent: A scoping review of its efficiency and technological prospecting. *Journal of Ethnopharmacology*, *287*, 114961. https://doi.org/10.1016/j.jep.2021.114961

Curtin, J. P., & Wang, M. (2017). Are clinical findings of systemic titanium dispersion following implantation explained by available in vitro evidence? An evidence-based analysis. *Journal of Biological Inorganic Chemistry : JBIC : A Publication of the Society of Biological Inorganic Chemistry*, *22*(6), 799–806. https://doi.org/10.1007/s00775-017-1464-1

d’Afflitto, M., Upadhyaya, A., Green, A., & Peiris, M. (2022). Association Between Sex Hormone Levels and Gut Microbiota Composition and Diversity-A Systematic Review. *Journal of Clinical Gastroenterology*, *56*(5), 384–392. https://doi.org/10.1097/MCG.0000000000001676

Decker, A., Askar, H., Tattan, M., Taichman, R., & Wang, H.-L. (2020). The assessment of stress, depression, and inflammation as a collective risk factor for periodontal diseases: A systematic review. *Clinical Oral Investigations*, *24*(1), 1–12. https://doi.org/10.1007/s00784-019-03089-3

Fournier, B., Bruneau, M., Toupenay, S., Kerner, S., Berdal, A., Cormier-Daire, V., Hadj-Rabia, S., Coudert, A., & de La Dure-Molla, M. (2018). Patterns of Dental Agenesis Highlight the Nature of the Causative Mutated Genes. *JOURNAL OF DENTAL RESEARCH*, *97*(12), 1306–1316. https://doi.org/10.1177/0022034518777460

Ginani, F., Soares, D. M., Barreto, M. P. E. V., & Barboza, C. A. G. (2015). Effect of low-level laser therapy on mesenchymal stem cell proliferation: A systematic review. *Lasers in Medical Science*, *30*(8), 2189–2194. https://doi.org/10.1007/s10103-015-1730-9

Gobin, R., Tian, D., Liu, Q., & Wang, J. (2020). Periodontal Diseases and the Risk of Metabolic Syndrome: An Updated Systematic Review and Meta-Analysis. *Frontiers in Endocrinology*, *11*, 336. https://doi.org/10.3389/fendo.2020.00336

Gonçalves, K. K. N., de Araújo, E. S. M., Barbirato, D. S., do Lago, C. A. P., & do Egito Vasconcelos, B. C. (2022). Head and neck cancer associated with myiasis. *International Journal of Oral and Maxillofacial Surgery*, *51*(7), 847–853. https://doi.org/10.1016/j.ijom.2021.08.011

Hindy, A., Farahmand, F., & Tabatabaei, F. S. (2017). In vitro biological outcome of laser application for modification or processing of titanium dental implants. *Lasers in Medical Science*, *32*(5), 1197–1206. https://doi.org/10.1007/s10103-017-2217-7

Hjalmarsson, L., Smedberg, J.-I., Aaronsson, G., & Wennerberg, A. (2011). Cellular responses to cobaltchrome and CP titanium—An in vitro comparison of frameworks for implant-retained oral prostheses. *Swedish Dental Journal*, *35*(4), 177–186. Scopus.

Huang, C., & Lee, B. (2015). Diagnosis of vertical root fracture in endodontically treated teeth using computed tomography. *JOURNAL OF DENTAL SCIENCES*, *10*(3), 227–232. https://doi.org/10.1016/j.jds.2015.01.002

Ikhar, A., Kolte, R., Kolte, A., Purohit, A., & Dahake, R. (n.d.). Efficacy of platelet rich fibrin with and without metformin in the treatment of periodontal osseous defects: A systematic review and meta-analysis. *ACTA ODONTOLOGICA SCANDINAVICA*. https://doi.org/10.1080/00016357.2022.2095024

Isola, G., Anastasi, G., Matarese, G., Williams, R., Cutroneo, G., Bracco, P., & Piancino, M. (2018). Functional and molecular outcomes of the human masticatory muscles. *ORAL DISEASES*, *24*(8), 1428–1441. https://doi.org/10.1111/odi.12806

Jain, A., & Taneja, S. (2022). Oral myiasis affecting paediatric patients: A systematic review. *Journal of Stomatology, Oral and Maxillofacial Surgery*, *123*(3), e32–e36. https://doi.org/10.1016/j.jormas.2021.07.006

Kan, J., Judge, R., & Palamara, J. (2014). In vitro bone strain analysis of implant following occlusal overload. *CLINICAL ORAL IMPLANTS RESEARCH*, *25*(2), E73–E82. https://doi.org/10.1111/clr.12059

Kaschwich, M., Behrendt, C.-A., Heydecke, G., Bayer, A., Debus, E. S., Seedorf, U., & Aarabi, G. (2019). The Association of Periodontitis and Peripheral Arterial Occlusive Disease-A Systematic Review. *International Journal of Molecular Sciences*, *20*(12). https://doi.org/10.3390/ijms20122936

Kaur, H., Kochhar, A. S., Gupta, H., Singh, G., & Kubavat, A. (2020). Appropriate orthodontic appliances during the COVID-19 pandemic: A scoping review. *Journal of Oral Biology and Craniofacial Research*, *10*(4), 782–787. Scopus. https://doi.org/10.1016/j.jobcr.2020.10.014

Kim, Y., Nowzari, H., & Rich, S. (2013). Risk of Prion Disease Transmission through Bovine-Derived Bone Substitutes: A Systematic Review. *CLINICAL IMPLANT DENTISTRY AND RELATED RESEARCH*, *15*(5), 645–653. https://doi.org/10.1111/j.1708-8208.2011.00407.x

Krastl, G., Allgayer, N., Lenherr, P., Filippi, A., Taneja, P., & Weiger, R. (2013). Tooth discoloration induced by endodontic materials: A literature review. *DENTAL TRAUMATOLOGY*, *29*(1), 2–7. https://doi.org/10.1111/j.1600-9657.2012.01141.x

Krastl, G., Weiger, R., Ebeleseder, K., & Galler, K. (n.d.). Present status and future directions: Endodontic management of traumatic injuries to permanent teeth. *INTERNATIONAL ENDODONTIC JOURNAL*. https://doi.org/10.1111/iej.13672

Kunert-Keil, C., Gredes, T., Richter, D.-U., Szyba, M., Dominiak, M., & Gedrange, T. (2012). The survival and proliferation of fibroblasts on ceramic implants: An in vitro study. *Biomedizinische Technik*, *57*(1), 11–15. Scopus. https://doi.org/10.1515/bmt-2011-0032

Laaksonen, M., Sorsa, T., & Salo, T. (2010). Emdogain in carcinogenesis: A systematic review of in vitro studies. *Journal of Oral Science*, *52*(1), 1–11. https://doi.org/10.2334/josnusd.52.1

Liu, T. J., Zhou, J. N., & Guo, L. H. (2021). Impact of different regenerative techniques and materials on the healing outcome of endodontic surgery: A systematic review and meta-analysis. *International Endodontic Journal*, *54*(4), 536–555. https://doi.org/10.1111/iej.13440

Liu, X., & Li, H. (2021). A Systematic Review and Meta-Analysis on Multiple Cytokine Gene Polymorphisms in the Pathogenesis of Periodontitis. *Frontiers in Immunology*, *12*, 713198. https://doi.org/10.3389/fimmu.2021.713198

Logan, D., Wallace, S. M., Woodside, J. V., & McKenna, G. (2021). The potential of salivary biomarkers of nutritional status and dietary intake: A Systematic Review. *Journal of Dentistry*, *115*, 103840. https://doi.org/10.1016/j.jdent.2021.103840

Ludwig, K. U., Ahmed, S. T., Böhmer, A. C., Sangani, N. B., Varghese, S., Klamt, J., Schuenke, H., Gültepe, P., Hofmann, A., Rubini, M., Aldhorae, K. A., Steegers-Theunissen, R. P., Rojas-Martinez, A., Reiter, R., Borck, G., Knapp, M., Nakatomi, M., Graf, D., Mangold, E., & Peters, H. (2016). Meta-analysis Reveals Genome-Wide Significance at 15q13 for Nonsyndromic Clefting of Both the Lip and the Palate, and Functional Analyses Implicate GREM1 As a Plausible Causative Gene. *PLoS Genetics*, *12*(3), e1005914. https://doi.org/10.1371/journal.pgen.1005914

Lv, L., Tang, Y., Zhang, P., Liu, Y., Bai, X., & Zhou, Y. (2018). Biomaterial Cues Regulate Epigenetic State and Cell Functions-A Systematic Review. *Tissue Engineering. Part B, Reviews*, *24*(2), 112–132. https://doi.org/10.1089/ten.teb.2017.0287

Manfredini, D., Ahlberg, J., Mura, R., & Lobbezoo, F. (2015). Bruxism Is Unlikely to Cause Damage to the Periodontium: Findings From a Systematic Literature Assessment. *JOURNAL OF PERIODONTOLOGY*, *86*(4), 546–555. https://doi.org/10.1902/jop.2014.140539

Mikulewicz, M., & Chojnacka, K. (2011). Cytocompatibility of medical biomaterials containing nickel by osteoblasts: A systematic literature review. *Biological Trace Element Research*, *142*(3), 865–889. https://doi.org/10.1007/s12011-010-8798-7

Mohammadrezaei, D., Golzar, H., Rezai Rad, M., Omidi, M., Rashedi, H., Yazdian, F., Khojasteh, A., & Tayebi, L. (2018). In vitro effect of graphene structures as an osteoinductive factor in bone tissue engineering: A systematic review. *Journal of Biomedical Materials Research. Part A*, *106*(8), 2284–2343. https://doi.org/10.1002/jbm.a.36422

Mousa, M. A., Abdullah, J. Y., Jamayet, N. B., Alam, M. K., & Husein, A. (2021). Biomechanical Stress in Obturator Prostheses: A Systematic Review of Finite Element Studies. *BioMed Research International*, *2021*, 6419774. https://doi.org/10.1155/2021/6419774

Możyńska, J., Metlerski, M., Lipski, M., & Nowicka, A. (2017). Tooth Discoloration Induced by Different Calcium Silicate-based Cements: A Systematic Review of In Vitro Studies. *Journal of Endodontics*, *43*(10), 1593–1601. https://doi.org/10.1016/j.joen.2017.04.002

Müller-Heupt, L. K., Schiegnitz, E., Kaya, S., Jacobi-Gresser, E., Kämmerer, P. W., & Al-Nawas, B. (2022). Diagnostic tests for titanium hypersensitivity in implant dentistry: A systematic review of the literature. *International Journal of Implant Dentistry*, *8*(1), 29. https://doi.org/10.1186/s40729-022-00428-0

Nibali, L., Koidou, V. P., Nieri, M., Barbato, L., Pagliaro, U., & Cairo, F. (2020). Regenerative surgery versus access flap for the treatment of intra-bony periodontal defects: A systematic review and meta-analysis. *Journal of Clinical Periodontology*, *47 Suppl 22*, 320–351. https://doi.org/10.1111/jcpe.13237

Ntrouka, V. I., Slot, D. E., Louropoulou, A., & Van der Weijden, F. (2011). The effect of chemotherapeutic agents on contaminated titanium surfaces: A systematic review. *Clinical Oral Implants Research*, *22*(7), 681–690. https://doi.org/10.1111/j.1600-0501.2010.02037.x

Pafitanis, G., Narushima, M., Yamamoto, T., Raveendran, M., Veljanoski, D., Ghanem, A. M., Myers, S., & Koshima, I. (2018). Evolution of an evidence-based supermicrosurgery simulation training curriculum: A systematic review. *Journal of Plastic, Reconstructive & Aesthetic Surgery : JPRAS*, *71*(7), 976–988. https://doi.org/10.1016/j.bjps.2018.04.005

Paños-Crespo, A., Sánchez-Torres, A., & Gay-Escoda, C. (2021). Retrograde filling material in periapical surgery: A systematic review. *Medicina Oral, Patologia Oral y Cirugia Bucal*, *26*(4), e422–e429. https://doi.org/10.4317/medoral.24262

Pardo-Aldave, K., Pareja-Vásquez, M., Guillén, A., & Ureta-Tapia, J. M. (2019). [Antimicrobial activity in vitro of Camu-Camu (Myrciaria Dubia) against oral microorganisms: A systematic review]. *Revista peruana de medicina experimental y salud publica*, *36*(4), 573–582. https://doi.org/10.17843/rpmesp.2019.364.4270

Paula, A. B., Toste, D., Marinho, A., Amaro, I., Marto, C.-M., Coelho, A., Marques-Ferreira, M., & Carrilho, E. (2019). Once Resin Composites and Dental Sealants Release Bisphenol-A, How Might This Affect Our Clinical Management?-A Systematic Review. *International Journal of Environmental Research and Public Health*, *16*(9). https://doi.org/10.3390/ijerph16091627

Pintor, A. V. B., Queiroz, L. D., Barcelos, R., Primo, L. S. G., Maia, L. C., & Alves, G. G. (2020). MTT versus other cell viability assays to evaluate the biocompatibility of root canal filling materials: A systematic review. *International Endodontic Journal*, *53*(10), 1348–1373. https://doi.org/10.1111/iej.13353

Raffat, M. A., Hadi, N. I., Hosein, M., Mirza, S., Ikram, S., & Akram, Z. (2018). S100 proteins in oral squamous cell carcinoma. *Clinica Chimica Acta; International Journal of Clinical Chemistry*, *480*, 143–149. https://doi.org/10.1016/j.cca.2018.02.013

Reis, A., Dourado Loguercio, A., Schroeder, M., Luque-Martinez, I., Masterson, D., & Cople Maia, L. (2015). Does the adhesive strategy influence the post-operative sensitivity in adult patients with posterior resin composite restorations?: A systematic review and meta-analysis. *Dental Materials : Official Publication of the Academy of Dental Materials*, *31*(9), 1052–1067. https://doi.org/10.1016/j.dental.2015.06.001

Rosen, E., Goldberger, T., Taschieri, S., Del Fabbro, M., Corbella, S., & Tsesis, I. (2016). The Prognosis of Altered Sensation after Extrusion of Root Canal Filling Materials: A Systematic Review of the Literature. *Journal of Endodontics*, *42*(6), 873–879. https://doi.org/10.1016/j.joen.2016.03.018

Sabado-Bundo, H., Sanchez-Garces, M., & Gay-Escoda, C. (2019). Bone regeneration in diabetic patients. A systematic review. *MEDICINA ORAL PATOLOGIA ORAL Y CIRUGIA BUCAL*, *24*(4), E425–E432. https://doi.org/10.4317/medoral.22889

Schroeder, F., Palma, V., Rados, P., & Visioli, F. (2022). Clinical and immunological features of chronic ulcerative stomatitis: A systematic review. *JOURNAL OF ORAL PATHOLOGY & MEDICINE*, *51*(6), 501–509. https://doi.org/10.1111/jop.13279

Sculean, A., Nikolidakis, D., Nikou, G., Ivanovic, A., Chapple, I. L. C., & Stavropoulos, A. (2015). Biomaterials for promoting periodontal regeneration in human intrabony defects: A systematic review. *Periodontology 2000*, *68*(1), 182–216. https://doi.org/10.1111/prd.12086

Starch-Jensen, T., Mordenfeld, A., Becktor, J. P., & Jensen, S. S. (2018). Maxillary Sinus Floor Augmentation With Synthetic Bone Substitutes Compared With Other Grafting Materials: A Systematic Review and Meta-analysis. *Implant Dentistry*, *27*(3), 363–374. https://doi.org/10.1097/ID.0000000000000768

Stasic, J. N., Pficer, J. K., Milicic, B., Puač, N., & Miletic, V. (2021). Effects of non-thermal atmospheric plasma on dentin wetting and adhesive bonding efficiency: Systematic review and meta-analysis. *Journal of Dentistry*, *112*, 103765. https://doi.org/10.1016/j.jdent.2021.103765

Stumbras, A., Krukis, M. M., Januzis, G., & Juodzbalys, G. (2019). Regenerative bone potential after sinus floor elevation using various bone graft materials: A systematic review. *Quintessence International (Berlin, Germany : 1985)*, *50*(7), 548–558. https://doi.org/10.3290/j.qi.a42482

Tallarico, M., Fiorellini, J., Nakajima, Y., Omori, Y., Takahisa, I., & Canullo, L. (2018). Mechanical Outcomes, Microleakage, and Marginal Accuracy at the Implant-Abutment Interface of Original versus Nonoriginal Implant Abutments: A Systematic Review of In Vitro Studies. *BioMed Research International*, *2018*, 2958982. https://doi.org/10.1155/2018/2958982

Tan, W., Wong, T., Wong, M., & Lang, N. (2012). A systematic review of post-extractional alveolar hard and soft tissue dimensional changes in humans. *CLINICAL ORAL IMPLANTS RESEARCH*, *23*, 1–21. https://doi.org/10.1111/j.1600-0501.2011.02375.x

Ting, M., Whitaker, E., & Albandar, J. (2016). Systematic review of the in vitro effects of statins on oral and perioral microorganisms. *EUROPEAN JOURNAL OF ORAL SCIENCES*, *124*(1), 4–10. https://doi.org/10.1111/eos.12239

Uzunoglu-Özyürek, E., Küçükkaya Eren, S., & Karahan, S. (2018). Effect of root canal sealers on the fracture resistance of endodontically treated teeth: A systematic review of in vitro studies. *Clinical Oral Investigations*, *22*(7), 2475–2485. https://doi.org/10.1007/s00784-018-2540-9

Vale, F. A., Moreira, M. S., de Almeida, F. C. S., & Ramalho, K. M. (2015). Low-level laser therapy in the treatment of recurrent aphthous ulcers: A systematic review. *TheScientificWorldJournal*, *2015*, 150412. https://doi.org/10.1155/2015/150412

Wessing, B., Lettner, S., & Zechner, W. (2018). Guided Bone Regeneration with Collagen Membranes and Particulate Graft Materials: A Systematic Review and Meta-Analysis. *The International Journal of Oral & Maxillofacial Implants*, *33*(1), 87–100. https://doi.org/10.11607/jomi.5461

Yamamoto, T., Li, M., Liu, Z., Guo, Y., Hasegawa, T., Masuki, H., Suzuki, R., & Amizuka, N. (2010). Histological review of the human cellular cementum with special reference to an alternating lamellar pattern. *ODONTOLOGY*, *98*(2), 102–109. https://doi.org/10.1007/s10266-010-0134-3

Yengopal, V., Chikte, U. M. E., Mickenautsch, S., Oliveira, L. B., & Bhayat, A. (2010). Salt fluoridation: A meta-analysis of its efficacy for caries prevention. *SADJ : Journal of the South African Dental Association = Tydskrif van Die Suid-Afrikaanse Tandheelkundige Vereniging*, *65*(2), 60–64, 66–67.

Yeung, C. A., Chong, L. Y., & Glenny, A.-M. (2015). Fluoridated milk for preventing dental caries. *The Cochrane Database of Systematic Reviews*, *2015*(9), CD003876. https://doi.org/10.1002/14651858.CD003876.pub4

Zhang, B., Huang, X., Huo, S., Zhang, C., Zhao, S., Cen, X., & Zhao, Z. (2020). Effect of clear aligners on oral health-related quality of life: A systematic review. *Orthodontics and Craniofacial Research*, *23*(4), 363–370. Scopus. https://doi.org/10.1111/ocr.12382

No dentistry topic

Abdelmoneim, A. S., Hasenbank, S. E., Seubert, J. M., Brocks, D. R., Light, P. E., & Simpson, S. H. (2012). Variations in tissue selectivity amongst insulin secretagogues: A systematic review. *Diabetes, Obesity & Metabolism*, *14*(2), 130–138. https://doi.org/10.1111/j.1463-1326.2011.01496.x

Adiguzel, D., & Celik-Ozenci, C. (2021). FoxO1 is a cell-specific core transcription factor for endometrial remodeling and homeostasis during menstrual cycle and early pregnancy. *Human Reproduction Update*, *27*(3), 570–583. Scopus. https://doi.org/10.1093/humupd/dmaa060

Ahmed, M., Best, L. M., Pereira, C. F., Boileau, I., & Kloiber, S. (2022). Effects of endocannabinoid system modulation on social behaviour: A systematic review of animal studies. *Neuroscience and Biobehavioral Reviews*, *138*, 104680. https://doi.org/10.1016/j.neubiorev.2022.104680

Alhassan, A., Young, J., Lean, M. E. J., & Lara, J. (2017). Consumption of fish and vascular risk factors: A systematic review and meta-analysis of intervention studies. *Atherosclerosis*, *266*, 87–94. https://doi.org/10.1016/j.atherosclerosis.2017.09.028

Alsalahi, A., Alshawsh, M. A., Mohamed, R., Alyousefi, N. A., Alshagga, M. A., Shwter, A. N., Al-Maqtari, A., Ahmed, R. H., & Mohamed, Z. (2016). Conflicting reports on the role of the glycemic effect of Catha edulis (Khat): A systematic review and meta-analysis. *Journal of Ethnopharmacology*, *186*, 30–43. https://doi.org/10.1016/j.jep.2016.03.045

Al-Waeli, H., Reboucas, A. P., Mansour, A., Morris, M., Tamimi, F., & Nicolau, B. (2021). Non-steroidal anti-inflammatory drugs and bone healing in animal models-a systematic review and meta-analysis. *Systematic Reviews*, *10*(1), 201. https://doi.org/10.1186/s13643-021-01690-w

Arockiam, A., Maheswari, R., Vijayalakshmi, R., & Abinaya, S. (2022). Can IGF-1 serve as a reliable skeletal maturity indicator? A meta-analysis. *JOURNAL OF OROFACIAL ORTHOPEDICS-FORTSCHRITTE DER KIEFERORTHOPADIE*, *83*(2), 124–140. https://doi.org/10.1007/s00056-021-00357-4

Artus, J., Hue, I., & Acloque, H. (2020). Preimplantation development in ungulates: A ‘ménage à quatre’ scenario. *Reproduction*, *159*(3), R151–R172. Scopus. https://doi.org/10.1530/REP-19-0348

Augustine, S., Cheng, W., Avey, M. T., Chan, M. L., Lingappa, S. M. C., Hutton, B., & Thébaud, B. (2020). Are all stem cells equal? Systematic review, evidence map, and meta-analyses of preclinical stem cell-based therapies for bronchopulmonary dysplasia. *Stem Cells Translational Medicine*, *9*(2), 158–168. https://doi.org/10.1002/sctm.19-0193

Ayudhya, C., Roy, S., Thapaliya, M., & Ali, H. (2020). Roles of a Mast Cell-Specific Receptor MRGPRX2 in Host Defense and Inflammation. *JOURNAL OF DENTAL RESEARCH*, *99*(8), 882–890. https://doi.org/10.1177/0022034520919107

Bando, Y., Sakashita, H., Nagasaka, A., Sakiyama, K., Tokuda, N., Iseki, S., Owada, Y., & Amano, O. (2022). Septoclasts expressing epidermal fatty acid-binding protein (E-FABP, FABP5) in endochondral ossification. *JOURNAL OF ORAL BIOSCIENCES*, *64*(1), 18–25. https://doi.org/10.1016/j.job.2021.12.003

Bazer, F. W. (2013). Pregnancy recognition signaling mechanisms in ruminants and pigs. *Journal of Animal Science and Biotechnology*, *4*(1). Scopus. https://doi.org/10.1186/2049-1891-4-23

Bazer, F. W., Song, G., Kim, J., Dunlap, K. A., Satterfield, M. C., Johnson, G. A., Burghardt, R. C., & Wu, G. (2012). Uterine biology in pigs and sheep. *Journal of Animal Science and Biotechnology*, *3*(1). Scopus. https://doi.org/10.1186/2050-7445-3-23

Bazer, F. W., Wang, X., Johnson, G. A., & Wu, G. (2015). Select nutrients and their effects on conceptus development in mammals. *Animal Nutrition*, *1*(3), 85–95. Scopus. https://doi.org/10.1016/j.aninu.2015.07.005

Bensadoun, R.-J., Epstein, J. B., Nair, R. G., Barasch, A., Raber-Durlacher, J. E., Migliorati, C., Genot-Klastersky, M.-T., Treister, N., Arany, P., Lodewijckx, J., & Robijns, J. (2020). Safety and efficacy of photobiomodulation therapy in oncology: A systematic review. *Cancer Medicine*, *9*(22), 8279–8300. https://doi.org/10.1002/cam4.3582

Binnekade, T. T., Van Kooten, J., Lobbezoo, F., Rhebergen, D., Van der Wouden, J. C., Smalbrugge, M., & Scherder, E. J. A. (2017). Pain Experience in Dementia Subtypes: A Systematic Review. *Current Alzheimer Research*, *14*(5), 471–485. https://doi.org/10.2174/1567205013666160602234109

Borges, G. A., Webber, L. P., M Marques, A. E., Guerra, E. N., Castilho, R. M., & Squarize, C. H. (2020). Pharmacological PTEN inhibition: Potential clinical applications and effects in tissue regeneration. *Regenerative Medicine*, *15*(2), 1329–1344. https://doi.org/10.2217/rme-2019-0065

Bottagisio, M., Coman, C., & Lovati, A. B. (2019). Animal models of orthopaedic infections. A review of rabbit models used to induce long bone bacterial infections. *Journal of Medical Microbiology*, *68*(4), 506–537. Scopus. https://doi.org/10.1099/jmm.0.000952

Bousnaki, M., Bakopoulou, A., Kritis, A., & Koidis, P. (2020). The Efficacy of Stem Cells Secretome Application in Osteoarthritis: A Systematic Review of In Vivo Studies. *Stem Cell Reviews and Reports*, *16*(6), 1222–1241. https://doi.org/10.1007/s12015-020-09980-x

Brickley, E. B., Coulibaly, M., Gabriel, E. E., Healy, S. A., Hume, J. C. C., Sagara, I., Traore, S. F., Doumbo, O., & Duffy, P. E. (2016). Utilizing direct skin feeding assays for development of vaccines that interrupt malaria transmission: A systematic review of methods and case study. *Vaccine*, *34*(48), 5863–5870. https://doi.org/10.1016/j.vaccine.2016.10.027

Bromfield, J. J. (2014). Seminal fluid and reproduction: Much more than previously thought. *Journal of Assisted Reproduction and Genetics*, *31*(6), 627–636. Scopus. https://doi.org/10.1007/s10815-014-0243-y

Brown, J., & Rapaport, B. (2019). Role of live animals in the training of microvascular surgery: A systematic review. *BRITISH JOURNAL OF ORAL & MAXILLOFACIAL SURGERY*, *57*(7), 616–619. https://doi.org/10.1016/j.bjoms.2019.06.003

Burli, A., Kashetsky, N., Feschuk, A., Law, R. M., & Maibach, H. I. (2021). Efficacy of soap and water based skin decontamination using in vivo animal models: A systematic review. *Journal of Toxicology and Environmental Health. Part B, Critical Reviews*, *24*(7), 325–336. https://doi.org/10.1080/10937404.2021.1943087

Canaan, M. M., Reis-Canaan, J. C., Zangerônimo, M. G., Andrade, E. F., Gonçalves, T. M. S. V., Pereira, M. C. A., Lima, R. R., Pardi, V., Murata, R. M., & Pereira, L. J. (2021). Yeast Beta-Glucans Ingestion Does Not Influence Body Weight: A Systematic Review and Meta-Analysis of Pre-Clinical Studies. *Nutrients*, *13*(12). https://doi.org/10.3390/nu13124250

Carrington, E. V., Evers, J., Grossi, U., Dinning, P. G., Scott, S. M., O’Connell, P. R., Jones, J. F. X., & Knowles, C. H. (2014). A systematic review of sacral nerve stimulation mechanisms in the treatment of fecal incontinence and constipation. *Neurogastroenterology and Motility : The Official Journal of the European Gastrointestinal Motility Society*, *26*(9), 1222–1237. https://doi.org/10.1111/nmo.12388

Carvalho, A. C. A. de, Souza, G. A. de, Marqui, S. V. de, Guiguer, É. L., Araújo, A. C., Rubira, C. J., Goulart, R. de A., Flato, U. A. P., Bueno, P. C. D. S., Buchaim, R. L., & Barbalho, S. M. (2020). Cannabis and Canabidinoids on the Inflammatory Bowel Diseases: Going Beyond Misuse. *International Journal of Molecular Sciences*, *21*(8). https://doi.org/10.3390/ijms21082940

Castejón-González, A. C., & Reiter, A. M. (2019). Locoregional Anesthesia of the Head. *Veterinary Clinics of North America - Small Animal Practice*, *49*(6), 1041–1061. Scopus. https://doi.org/10.1016/j.cvsm.2019.07.011

Cha, J., Sun, X., & Dey, S. K. (2012). Mechanisms of implantation: Strategies for successful pregnancy. *Nature Medicine*, *18*(12), 1754–1767. Scopus. https://doi.org/10.1038/nm.3012

Chiang, C., Kashetsky, N., Feschuk, A., Burli, A., Law, R. M., & Maibach, H. I. (2022). Efficacy of water-only or soap and water skin decontamination of chemical warfare agents or simulants using in vitro human models: A systematic review. *Journal of Applied Toxicology : JAT*, *42*(6), 930–941. https://doi.org/10.1002/jat.4251

Chiang, C., Kashetsky, N., Feschuk, A., Burli, A., Law, R., & Maibach, H. (2021). Efficacy of water-based skin decontamination of occupational chemicals using in vitro human skin models: A systematic review. *Journal of Toxicology and Environmental Health. Part B, Critical Reviews*, *24*(7), 337–353. https://doi.org/10.1080/10937404.2021.1957048

Chinipardaz, Z., Liu, M., Graves, D., & Yang, S. (2022). Role of Primary Cilia in Bone and Cartilage. *JOURNAL OF DENTAL RESEARCH*, *101*(3), 253–260. https://doi.org/10.1177/00220345211046606

Chisari, E., Rehak, L., Khan, W. S., & Maffulli, N. (2019). Tendon healing in presence of chronic low-level inflammation: A systematic review. *British Medical Bulletin*, *132*(1), 97–116. https://doi.org/10.1093/bmb/ldz035

Chisini, L. A., Conde, M. C. M., Grazioli, G., Martin, A. S. S., Carvalho, R. V. de, Nör, J. E., & Demarco, F. F. (2017). Venous Blood Derivatives as FBS-Substitutes for Mesenchymal Stem Cells: A Systematic Scoping Review. *Brazilian Dental Journal*, *28*(6), 657–668. https://doi.org/10.1590/0103-6440201701646

Chiu, B., Jantuan, E., Shen, F., Chiu, B., & Sergi, C. (2017). Autophagy-Inflammasome Interplay in Heart Failure: A Systematic Review on Basics, Pathways, and Therapeutic Perspectives. *Annals of Clinical and Laboratory Science*, *47*(3), 243–252.

Chuinsiri, N., Edwards, D., Telezhkin, V., Nile, C., Van der Cruyssen, F., & Durham, J. (2021). Exploring the roles of neuropeptides in trigeminal neuropathic pain: A systematic review and narrative synthesis of animal studies. *ARCHIVES OF ORAL BIOLOGY*, *130*. https://doi.org/10.1016/j.archoralbio.2021.105247

Claussen, A. D., Quevedo, R. V., Kirk, J. R., Higgins, T., Mostaert, B., Rahman, M. T., Oleson, J., Hernandez, R., Hirose, K., & Hansen, M. R. (2022). Chronic cochlear implantation with and without electric stimulation in a mouse model induces robust cochlear influx of CX3CR1+/GFP macrophages. *Hearing Research*. Scopus. https://doi.org/10.1016/j.heares.2022.108510

Crowe, W., Allsopp, P. J., Watson, G. E., Magee, P. J., Strain, J. J., Armstrong, D. J., Ball, E., & McSorley, E. M. (2017). Mercury as an environmental stimulus in the development of autoimmunity—A systematic review. *Autoimmunity Reviews*, *16*(1), 72–80. https://doi.org/10.1016/j.autrev.2016.09.020

da Silva, V. P., Mesquita, C. B., Nunes, J. S., de Bem Prunes, B., Rados, P. V., & Visioli, F. (2018). Effects of extracellular acidity on resistance to chemotherapy treatment: A systematic review. *Medical Oncology (Northwood, London, England)*, *35*(12), 161. https://doi.org/10.1007/s12032-018-1214-4

de Bartolomeis, A., Barone, A., Begni, V., & Riva, M. A. (2022). Present and future antipsychotic drugs: A systematic review of the putative mechanisms of action for efficacy and a critical appraisal under a translational perspective. *Pharmacological Research*, *176*, 106078. https://doi.org/10.1016/j.phrs.2022.106078

Dean, M. (2019). Glycogen in the uterus and fallopian tubes is an important source of glucose during early pregnancy. *Biology of Reproduction*, *101*(2), 297–305. Scopus. https://doi.org/10.1093/biolre/ioz102

deCatanzaro, D. (2015). Sex steroids as pheromones in mammals: The exceptional role of estradiol. *Hormones and Behavior*, *68*, 103–116. Scopus. https://doi.org/10.1016/j.yhbeh.2014.08.003

Delic, N. C., Cai, J. R., Watson, S. L., Downie, L. E., & Di Girolamo, N. (2022). Evaluating the clinical translational relevance of animal models for limbal stem cell deficiency: A systematic review. *The Ocular Surface*, *23*, 169–183. https://doi.org/10.1016/j.jtos.2021.09.006

Dsouza, C., & Komarova, S. V. (2021). Characterization of Potency of the P2Y13 Receptor Agonists: A Meta-Analysis. *International Journal of Molecular Sciences*, *22*(7). https://doi.org/10.3390/ijms22073468

El-Qushayri, A. E., Kamel, A. M. A., Faraj, H. A., Vuong, N. L., Diab, O. M., Istanbuly, S., Elshafei, T. A., Makram, O. M., Sattar, Z., Istanbuly, O., Mukit, S. A. A., Elfaituri, M. K., Low, S. K., & Huy, N. T. (2020). Association between pet ownership and cardiovascular risks and mortality: A systematic review and meta-analysis. *Journal of Cardiovascular Medicine (Hagerstown, Md.)*, *21*(5), 359–367. https://doi.org/10.2459/JCM.0000000000000920

Essig, G. F. J., Sheehan, C., Rikhi, S., Elmaraghy, C. A., & Christophel, J. J. (2019). Dog bite injuries to the face: Is there risk with breed ownership? A systematic review with meta-analysis. *International Journal of Pediatric Otorhinolaryngology*, *117*, 182–188. https://doi.org/10.1016/j.ijporl.2018.11.028

Ferreux, L., Firmin, J., Pocate-Cheriet, K., & Patrat, C. (2019). What do post-implantation extended culture models tell us about the development of the human embryo? *Medecine de la Reproduction*, *21*(1), 69–77. Scopus. https://doi.org/10.1684/mte.2019.0736

Fiani, N., Verstraete, F. J. M., & Arzi, B. (2016). Reconstruction of Congenital Nose, Cleft Primary Palate, and Lip Disorders. *Veterinary Clinics of North America - Small Animal Practice*, *46*(4), 663–675. Scopus. https://doi.org/10.1016/j.cvsm.2016.02.001

Fischer, B., Chavatte-Palmer, P., Viebahn, C., Santos, A., & Duranthon, V. (2012). Rabbit as a reproductive model for human health. *Reproduction*, *144*(1), 1–10. Scopus. https://doi.org/10.1530/REP-12-0091

Fujita-Yoshigaki, J., Yokoyama, M., & Katsumata-Kato, O. (2017). Determinants for selective transport of exogenously expressed cargo proteins into regulated and constitutive secretory pathways. *JOURNAL OF ORAL BIOSCIENCES*, *59*(2), 87–91. https://doi.org/10.1016/j.job.2017.01.001

Fukui, Y., Hirota, Y., Matsuo, M., Gebril, M., Akaeda, S., Hiraoka, T., & Osuga, Y. (2019). Uterine receptivity, embryo attachment, and embryo invasion: Multistep processes in embryo implantation. *Reproductive Medicine and Biology*, *18*(3), 234–240. Scopus. https://doi.org/10.1002/rmb2.12280

Gamirova, A., Berbenyuk, A., Levina, D., Peshko, D., Simpson, M. R., Azad, M. B., Järvinen, K. M., Brough, H. A., Genuneit, J., Greenhawt, M., Verhasselt, V., Peroni, D. G., Perkin, M. R., Warner, J. O., Palmer, D. J., Boyle, R. J., & Munblit, D. (2022). Food Proteins in Human Breast Milk and Probability of IgE-Mediated Allergic Reaction in Children During Breastfeeding: A Systematic Review. *The Journal of Allergy and Clinical Immunology. In Practice*, *10*(5), 1312-1324.e8. https://doi.org/10.1016/j.jaip.2022.01.028

Geisert, R. D., Lucy, M. C., Whyte, J. J., Ross, J. W., & Mathew, D. J. (2014). Cytokines from the pig conceptus: Roles in conceptus development in pigs. *Journal of Animal Science and Biotechnology*, *5*(1). Scopus. https://doi.org/10.1186/2049-1891-5-51

Geisert, R. D., Meyer, A. E., Pfeiffer, C. A., Johns, D. N., Lee, K., Wells, K. D., Spencer, T. E., & Prather, R. S. (2021). Gene editing to investigate the role of conceptus factors in the establishment of pregnancy in the pig. *Reproduction*, *161*(4), R79–R88. Scopus. https://doi.org/10.1530/REP-20-0604

Golledge, J., & Thanigaimani, S. (2022). Role of Sclerostin in Cardiovascular Disease. *Arteriosclerosis, Thrombosis, and Vascular Biology*, *42*(7), e187–e202. https://doi.org/10.1161/ATVBAHA.122.317635

Grosso, M. C., Bellingeri, R. V., Motta, C. E., Alustiza, F. E., Picco, N. Y., & Vivas, A. B. (2015). Immunohistochemical distribution of early pregnancy factor in ovary, oviduct and placenta of pregnant gilts. *Biotechnic and Histochemistry*, *90*(1), 14–24. Scopus. https://doi.org/10.3109/10520295.2014.931599

Guerra, E. N. S., Rêgo, D. F., Elias, S. T., Coletta, R. D., Mezzomo, L. A. M., Gozal, D., & De Luca Canto, G. (2016). Diagnostic accuracy of serum biomarkers for head and neck cancer: A systematic review and meta-analysis. *Critical Reviews in Oncology/Hematology*, *101*, 93–118. https://doi.org/10.1016/j.critrevonc.2016.03.002

Gupta, A. A., Kheur, S., Arakeri, G., Thirumal Raj, A., Badhe, R. V., Patil, S., Rao Us, V., Patil, S., Gomez, R. S., Thomson, P., & Brennan, P. A. (2020). Efficacy of scaffold-mediated localized chemotherapy in cancer: A systematic review of current research. *Journal of Oral Pathology & Medicine : Official Publication of the International Association of Oral Pathologists and the American Academy of Oral Pathology*, *49*(5), 375–385. https://doi.org/10.1111/jop.12994

Hambly, J. L., Khan, S., McDermott, B., Bor, W., & Haywood, A. (2016). Pharmacotherapy of conduct disorder: Challenges, options and future directions. *Journal of Psychopharmacology (Oxford, England)*, *30*(10), 967–975. https://doi.org/10.1177/0269881116658985

Hancock, G. V., Wamaitha, S. E., Peretz, L., & Clark, A. T. (2021). Mammalian primordial germ cell specification. *Development (Cambridge)*, *148*(6). Scopus. https://doi.org/10.1242/dev.189217

Hao, J., Stavljenić Milašin, I., Batu Eken, Z., Mravak-Stipetic, M., Pavelić, K., & Ozer, F. (2021). Effects of Zeolite as a Drug Delivery System on Cancer Therapy: A Systematic Review. *Molecules (Basel, Switzerland)*, *26*(20). https://doi.org/10.3390/molecules26206196

Hassan, M. N., Yassin, M. A., Suliman, S., Lie, S. A., Gjengedal, H., & Mustafa, K. (2019). The bone regeneration capacity of 3D-printed templates in calvarial defect models: A systematic review and meta-analysis. *Acta Biomaterialia*, *91*, 1–23. https://doi.org/10.1016/j.actbio.2019.04.017

Hayes, K., Kim, Y.-K., & Pera, M. F. (2021). A case for revisiting Nodal signaling in human pluripotent stem cells. *Stem Cells*, *39*(9), 1137–1144. Scopus. https://doi.org/10.1002/stem.3383

Hodges, V., Hynes, C., Lassa, S., & Mitchell, C. (2021). Support needs of carers making proxy healthcare decisions for people with dementia: A systematic review based on the Noblit and Hare meta-ethnographic synthesis of qualitative studies. *BMJ Open*, *11*(12), e052608. https://doi.org/10.1136/bmjopen-2021-052608

Hosford, P. S., & Gourine, A. V. (2019). What is the key mediator of the neurovascular coupling response? *Neuroscience and Biobehavioral Reviews*, *96*, 174–181. https://doi.org/10.1016/j.neubiorev.2018.11.011

Hunt, K. J., Hung, S. K., & Ernst, E. (2010). Botanical extracts as anti-aging preparations for the skin: A systematic review. *Drugs & Aging*, *27*(12), 973–985. https://doi.org/10.2165/11584420-000000000-00000

Islam, M. T., Ali, E. S., Uddin, S. J., Shaw, S., Islam, M. A., Ahmed, M. I., Chandra Shill, M., Karmakar, U. K., Yarla, N. S., Khan, I. N., Billah, M. M., Pieczynska, M. D., Zengin, G., Malainer, C., Nicoletti, F., Gulei, D., Berindan-Neagoe, I., Apostolov, A., Banach, M., … Atanasov, A. G. (2018). Phytol: A review of biomedical activities. *Food and Chemical Toxicology : An International Journal Published for the British Industrial Biological Research Association*, *121*, 82–94. https://doi.org/10.1016/j.fct.2018.08.032

Ismail, M., Alsalahi, A., Imam, M. U., Ooi, D. J., Khaza’ai, H., Aljaberi, M. A., Shamsudin, M. N., & Idrus, Z. (2020). Safety and Neuroprotective Efficacy of Palm Oil and Tocotrienol-Rich Fraction from Palm Oil: A Systematic Review. *Nutrients*, *12*(2). https://doi.org/10.3390/nu12020521

Jensen, D. H., Oliveri, R. S., Trojahn Kølle, S.-F., Fischer-Nielsen, A., Specht, L., Bardow, A., & Buchwald, C. (2014). Mesenchymal stem cell therapy for salivary gland dysfunction and xerostomia: A systematic review of preclinical studies. *Oral Surgery, Oral Medicine, Oral Pathology and Oral Radiology*, *117*(3), 335-342.e1. https://doi.org/10.1016/j.oooo.2013.11.496

Jepson, R. E., Warren, H., Wallace, M. D., Syme, H. M., Elliott, J., & Munroe, P. B. (2022). First genome-wide association study investigating blood pressure and renal traits in domestic cats. *Scientific Reports*, *12*(1), 1899. https://doi.org/10.1038/s41598-022-05494-3

Johnson, G. A., Burghardt, R. C., & Bazer, F. W. (2014). Osteopontin: A leading candidate adhesion molecule for implantation in pigs and sheep. *Journal of Animal Science and Biotechnology*, *5*(1). Scopus. https://doi.org/10.1186/2049-1891-5-56

Johnson, G. A., Seo, H., Bazer, F. W., Wu, G., Kramer, A. C., McLendon, B. A., & Cain, J. W. (2022). Metabolic pathways utilized by the porcine conceptus, uterus, and placenta. *Molecular Reproduction and Development*. Scopus. https://doi.org/10.1002/mrd.23570

Juodzbalys, G., Kasradze, D., Cicciù, M., Sudeikis, A., Banys, L., Galindo-Moreno, P., & Guobis, Z. (2016). Modern molecular biomarkers of head and neck cancer. Part I. Epigenetic diagnostics and prognostics: Systematic review. *Cancer Biomarkers : Section A of Disease Markers*, *17*(4), 487–502. https://doi.org/10.3233/CBM-160666

Justice, A. E., Karaderi, T., Highland, H. M., Young, K. L., Graff, M., Lu, Y., Turcot, V., Auer, P. L., Fine, R. S., Guo, X., Schurmann, C., Lempradl, A., Marouli, E., Mahajan, A., Winkler, T. W., Locke, A. E., Medina-Gomez, C., Esko, T., Vedantam, S., … Lindgren, C. M. (2019). Protein-coding variants implicate novel genes related to lipid homeostasis contributing to body-fat distribution. *Nature Genetics*, *51*(3), 452–469. https://doi.org/10.1038/s41588-018-0334-2

Juurikka, K., Butler, G. S., Salo, T., Nyberg, P., & Åström, P. (2019). The Role of MMP8 in Cancer: A Systematic Review. *International Journal of Molecular Sciences*, *20*(18). https://doi.org/10.3390/ijms20184506

Kabir, W., Di Bella, C., Jo, I., Gould, D., & Choong, P. F. M. (2021). Human Stem Cell Based Tissue Engineering for In Vivo Cartilage Repair: A Systematic Review. *Tissue Engineering. Part B, Reviews*, *27*(1), 74–93. https://doi.org/10.1089/ten.TEB.2020.0155

Kafer, G. R., & Cesare, A. J. (2020). A Survey of Essential Genome Stability Genes Reveals That Replication Stress Mitigation Is Critical for Peri-Implantation Embryogenesis. *Frontiers in Cell and Developmental Biology*, *8*. Scopus. https://doi.org/10.3389/fcell.2020.00416

Kaibuchi, N., Iwata, T., Koga, Y. K., & Okamoto, T. (2022). Novel Cell Therapy Using Mesenchymal Stromal Cell Sheets for Medication-Related Osteonecrosis of the Jaw. *Frontiers in Bioengineering and Biotechnology*, *10*. Scopus. https://doi.org/10.3389/fbioe.2022.902349

Kaklamanos, E. G., Makrygiannakis, M. A., & Athanasiou, A. E. (2020). Does medication administration affect the rate of orthodontic tooth movement and root resorption development in humans? A systematic review. *European Journal of Orthodontics*, *42*(4), 407–414. https://doi.org/10.1093/ejo/cjz063

Kalladka, M., Nasri-Heir, C., Eliav, E., Ananthan, S., Viswanath, A., & Heir, G. (2016). Continuous neuropathic pain secondary to endoscopic procedures: Report of two cases and review of the literature. *ORAL SURGERY ORAL MEDICINE ORAL PATHOLOGY ORAL RADIOLOGY*, *122*(2), E55–E59. https://doi.org/10.1016/j.oooo.2016.05.012

Kanematsu, T., Oue, K., Okumura, T., Harada, K., Yamawaki, Y., Asano, S., Mizokami, A., Irifune, M., & Hirata, M. (2019). Phospholipase C-related catalytically inactive protein: A novel signaling molecule for modulating fat metabolism and energy expenditure. *JOURNAL OF ORAL BIOSCIENCES*, *61*(2), 65–72. https://doi.org/10.1016/j.job.2019.04.002

Khayat, M., Lois, N., Williams, M., & Stitt, A. W. (2017). Animal Models of Retinal Vein Occlusion. *Investigative Ophthalmology & Visual Science*, *58*(14), 6175–6192. https://doi.org/10.1167/iovs.17-22788

Khosh, E., Bahmaie, N., Elahi, R., & Esmaeilzadeh, A. (2020). Clinical Applications of Interleukin-37: A Key Player in the Immunopathogenesis of Immune Disorders. *Iranian Journal of Allergy, Asthma, and Immunology*, *19*(3), 209–228. https://doi.org/10.18502/ijaai.v19i3.3450

Khoury, S., Wang, Q.-P., Parisien, M., Gris, P., Bortsov, A. V., Linnstaedt, S. D., McLean, S. A., Tungate, A. S., Sofer, T., Lee, J., Louie, T., Redline, S., Kaunisto, M. A., Kalso, E. A., Munter, H. M., Nackley, A. G., Slade, G. D., Smith, S. B., Zaykin, D. V., … Diatchenko, L. (2021). Multi-ethnic GWAS and meta-analysis of sleep quality identify MPP6 as a novel gene that functions in sleep center neurons. *Sleep*, *44*(3). https://doi.org/10.1093/sleep/zsaa211

Kim, Y. S., & Bedzhov, I. (2022). Mechanisms of formation and functions of the early embryonic cavities. *Seminars in Cell and Developmental Biology*. Scopus. https://doi.org/10.1016/j.semcdb.2022.04.020

Knapik, D. M., Harris, J. D., Pangrazzi, G., Griesser, M. J., Siston, R. A., Agarwal, S., & Flanigan, D. C. (2013). The basic science of continuous passive motion in promoting knee health: A systematic review of studies in a rabbit model. *Arthroscopy : The Journal of Arthroscopic & Related Surgery : Official Publication of the Arthroscopy Association of North America and the International Arthroscopy Association*, *29*(10), 1722–1731. https://doi.org/10.1016/j.arthro.2013.05.028

Kobayashi, M., & Nakaya, Y. (2020). Anatomical aspects of corticotrigeminal projections to the medullary dorsal horn. *JOURNAL OF ORAL SCIENCE*, *62*(2), 144–146. https://doi.org/10.2334/josnusd.19-0386

Korczeniewska, O. A., Kohli, D., Katzmann Rider, G., Zaror, C., Iturriaga, V., & Benoliel, R. (2021). Effects of melanocortin-4 receptor (MC4R) antagonist on neuropathic pain hypersensitivity in rats—A systematic review and meta-analysis. *European Journal of Oral Sciences*, *129*(4), e12786. https://doi.org/10.1111/eos.12786

Kumada, A., Matsuka, Y., Spigelman, I., Maruhama, K., Yamamoto, Y., Neubert, J., Nolan, T., Watanabe, K., Maekawa, K., Kamioka, H., Yamashiro, T., Kuboki, T., & Oguma, K. (2012). Intradermal injection of Botulinum toxin type A alleviates infraorbital nerve constriction-induced thermal hyperalgesia in an operant assay. *JOURNAL OF ORAL REHABILITATION*, *39*(1), 63–72. https://doi.org/10.1111/j.1365-2842.2011.02236.x

LaCroix-Fralish, M. L., Austin, J.-S., Zheng, F. Y., Levitin, D. J., & Mogil, J. S. (2011). Patterns of pain: Meta-analysis of microarray studies of pain. *Pain*, *152*(8), 1888–1898. https://doi.org/10.1016/j.pain.2011.04.014

Langdon, K., Phie, J., Thapa, C. B., Biros, E., Loukas, A., & Haleagrahara, N. (2019). Helminth-based therapies for rheumatoid arthritis: A systematic review and meta-analysis. *International Immunopharmacology*, *66*, 366–372. https://doi.org/10.1016/j.intimp.2018.11.034

Liewen, C., Krenn, V. T., Arens, N., Dierkes, C., & Krenn, V. (2021). Joint tumors: Rare but important differential diagnoses of malignant and benign tumors as well as pseudotumors in rheumatology. *Zeitschrift fur Rheumatologie*, *80*(2), 165–175. Scopus. https://doi.org/10.1007/s00393-020-00936-7

Lu, Y., Day, F. R., Gustafsson, S., Buchkovich, M. L., Na, J., Bataille, V., Cousminer, D. L., Dastani, Z., Drong, A. W., Esko, T., Evans, D. M., Falchi, M., Feitosa, M. F., Ferreira, T., Hedman, Å. K., Haring, R., Hysi, P. G., Iles, M. M., Justice, A. E., … Loos, R. J. F. (2016). New loci for body fat percentage reveal link between adiposity and cardiometabolic disease risk. *Nature Communications*, *7*, 10495. https://doi.org/10.1038/ncomms10495

Luo, X., Qiu, Y., Dinesh, P., Gong, W., Jiang, L., Feng, X., Li, J., Jiang, Y., Lei, Y. L., & Chen, Q. (2021). The functions of autophagy at the tumour-immune interface. *Journal of Cellular and Molecular Medicine*, *25*(5), 2333–2341. https://doi.org/10.1111/jcmm.16331

Lyu, C., Li, W., Liu, S., Gao, S., Zhang, H., Hao, L., Yu, H., Wei, W., Song, J., Yang, Y., Wang, C., Zhang, Z., & Wang, N. (2018). Systematic review on the efficacy and safety of immune checkpoint inhibition in renal cell carcinoma. *Future Oncology (London, England)*, *14*(21), 2207–2221. https://doi.org/10.2217/fon-2018-0193

Maffulli, N., Rodriguez, H. C., Stone, I. W., Nam, A., Song, A., Gupta, M., Alvarado, R., Ramon, D., & Gupta, A. (2020). Artificial intelligence and machine learning in orthopedic surgery: A systematic review protocol. *Journal of Orthopaedic Surgery and Research*, *15*(1), 478. https://doi.org/10.1186/s13018-020-02002-z

Maroulakos, M., Kamperos, G., Tayebi, L., Halazonetis, D., & Ren, Y. (2019). Applications of 3D printing on craniofacial bone repair: A systematic review. *Journal of Dentistry*, *80*, 1–14. https://doi.org/10.1016/j.jdent.2018.11.004

Martin, S., Foulon, A., El Hage, W., Dufour-Rainfray, D., & Denis, F. (2022). Is There a Link between Oropharyngeal Microbiome and Schizophrenia? A Narrative Review. *International Journal of Molecular Sciences*, *23*(2). https://doi.org/10.3390/ijms23020846

Matsumoto, H. (2017). Molecular and cellular events during blastocyst implantation in the receptive uterus: Clues from mouse models. *Journal of Reproduction and Development*, *63*(5), 445–454. Scopus. https://doi.org/10.1262/jrd.2017-047

Matsumoto, H., Fukui, E., & Yoshizawa, M. (2015). Angiogenesis and hormonal regulation on uterine receptivity for blastocyst implantation. *Journal of Mammalian Ova Research*, *32*(3), 79–85. Scopus. https://doi.org/10.1274/jmor.32.79

Matsumoto, H., Fukui, E., & Yoshizawa, M. (2016). Molecular and cellular events involved in the completion of blastocyst implantation. *Reproductive Medicine and Biology*, *15*(2), 53–58. Scopus. https://doi.org/10.1007/s12522-015-0222-8

Mercurio, A. (2011). Complications of upper airway surgery in companion animals. *Veterinary Clinics of North America - Small Animal Practice*, *41*(5), 969–980. Scopus. https://doi.org/10.1016/j.cvsm.2011.05.016

Mich, P. M. (2014). The Emerging Role of Veterinary Orthotics and Prosthetics (V-OP) in Small Animal Rehabilitation and Pain Management. *Topics in Companion Animal Medicine*, *29*(1), 10–19. Scopus. https://doi.org/10.1053/j.tcam.2014.04.002

Mielgo, V., Valls i Soler, A., & Rey-Santano, C. (2014). Dobutamine in paediatric population: A systematic review in juvenile animal models. *PloS One*, *9*(4), e95644. https://doi.org/10.1371/journal.pone.0095644

Mikolajewicz, N., Mohammed, A., Morris, M., & Komarova, S. V. (2018). Mechanically stimulated ATP release from mammalian cells: Systematic review and meta-analysis. *Journal of Cell Science*, *131*(22), jcs223354. https://doi.org/10.1242/jcs.223354

Miles, J. R., Walsh, S. C., Rempel, L. A., & Pannier, A. K. (2022). Mechanisms regulating the initiation of porcine conceptus elongation. *Molecular Reproduction and Development*. Scopus. https://doi.org/10.1002/mrd.23623

Miyake, Y., Takaki, A., Iwasaki, Y., & Yamamoto, K. (2010). Meta-analysis: Interferon-alpha prevents the recurrence after curative treatment of hepatitis C virus-related hepatocellular carcinoma. *Journal of Viral Hepatitis*, *17*(4), 287–292. https://doi.org/10.1111/j.1365-2893.2009.01181.x

Mohamed Omer, S., Krishna, S. M., Li, J., Moxon, J. V., Nsengiyumva, V., & Golledge, J. (2016). The efficacy of extraembryonic stem cells in improving blood flow within animal models of lower limb ischaemia. *Heart (British Cardiac Society)*, *102*(1), 69–74. https://doi.org/10.1136/heartjnl-2015-308322

Moran, M. M., Wilson, B. M., Ross, R. D., Virdi, A. S., & Sumner, D. R. (2017). Arthrotomy-based preclinical models of particle-induced osteolysis: A systematic review. *Journal of Orthopaedic Research*, *35*(12), 2595–2605. Scopus. https://doi.org/10.1002/jor.23619

Moretti, A., Paoletta, M., Liguori, S., Bertone, M., Toro, G., & Iolascon, G. (2020). Choline: An Essential Nutrient for Skeletal Muscle. *Nutrients*, *12*(7). https://doi.org/10.3390/nu12072144

Nakamura, T., Fujiwara, K., Saitou, M., & Tsukiyama, T. (2021). Non-human primates as a model for human development. *Stem Cell Reports*, *16*(5), 1093–1103. Scopus. https://doi.org/10.1016/j.stemcr.2021.03.021

Niño-Sandoval, T. C., Rodrigues, E. D. R., & Vasconcelos, B. C. (2021). Latency phase in mandibular distraction osteogenesis: A systematic review in animal models. *The British Journal of Oral & Maxillofacial Surgery*, *59*(9), 993–1004. https://doi.org/10.1016/j.bjoms.2020.12.012

Niwa, H. (2010). Mouse ES cell culture system as a model of development. *Development Growth and Differentiation*, *52*(3), 275–283. Scopus. https://doi.org/10.1111/j.1440-169X.2009.01166.x

Noguchi, S., Saito, A., Horie, M., Mikami, Y., Suzuki, H. I., Morishita, Y., Ohshima, M., Abiko, Y., Mattsson, J. S. M., König, H., Lohr, M., Edlund, K., Botling, J., Micke, P., & Nagase, T. (2014). An integrative analysis of the tumorigenic role of TAZ in human non-small cell lung cancer. *Clinical Cancer Research : An Official Journal of the American Association for Cancer Research*, *20*(17), 4660–4672. https://doi.org/10.1158/1078-0432.CCR-13-3328

Oloyo, A. K., Ambele, M. A., & Pepper, M. S. (2018). Contrasting Views on the Role of Mesenchymal Stromal/Stem Cells in Tumour Growth: A Systematic Review of Experimental Design. *Advances in Experimental Medicine and Biology*, *1083*, 103–124. https://doi.org/10.1007/5584_2017_118

Olsson, D. C., Teixeira, B. L., Jeremias, T. D. S., Réus, J. C., De Luca Canto, G., Porporatti, A. L., & Trentin, A. G. (2021). Administration of mesenchymal stem cells from adipose tissue at the hip joint of dogs with osteoarthritis: A systematic review. *Research in Veterinary Science*, *135*, 495–503. https://doi.org/10.1016/j.rvsc.2020.11.014

Ouwehand, A. C., Invernici, M. M., Furlaneto, F. A. C., & Messora, M. R. (2018). Effectiveness of Multistrain Versus Single-strain Probiotics: Current Status and Recommendations for the Future. *Journal of Clinical Gastroenterology*, *52 Suppl 1, Proceedings from the 9th Probiotics, Prebiotics and New Foods, Nutraceuticals and Botanicals for Nutrition&Human and Microbiota Health Meeting, held in Rome, Italy from September 10 to 12, 2017*, S35–S40. https://doi.org/10.1097/MCG.0000000000001052

Phie, J., Krishna, S. M., Moxon, J. V., Omer, S. M., Kinobe, R., & Golledge, J. (2017). Flavonols reduce aortic atherosclerosis lesion area in apolipoprotein E deficient mice: A systematic review and meta-analysis. *PloS One*, *12*(7), e0181832. https://doi.org/10.1371/journal.pone.0181832

Phie, J., Thanigaimani, S., & Golledge, J. (2021). Systematic Review and Meta-Analysis of Interventions to Slow Progression of Abdominal Aortic Aneurysm in Mouse Models. *Arteriosclerosis, Thrombosis, and Vascular Biology*, *41*(4), 1504–1517. https://doi.org/10.1161/ATVBAHA.121.315942

Purdue, P. E., Levin, A. S., Ren, K., Sculco, T. P., Wang, D., & Goldring, S. R. (2013). Development of Polymeric Nanocarrier System for Early Detection and Targeted Therapeutic Treatment of Peri-Implant Osteolysis. *HSS Journal*, *9*(1), 79–85. Scopus. https://doi.org/10.1007/s11420-012-9307-7

Puty, B., Leão, L. K. R., Crespo-Lopez, M. E., Almeida, A. P. C. P. S. C., Fagundes, N. C. F., Maia, L. C., & Lima, R. R. (2019). Association between methylmercury environmental exposure and neurological disorders: A systematic review. *Journal of Trace Elements in Medicine and Biology : Organ of the Society for Minerals and Trace Elements (GMS)*, *52*, 100–110. https://doi.org/10.1016/j.jtemb.2018.12.001

Rastegar-Pouyani, S., Khazaei, N., Wee, P., Yaqubi, M., & Mohammadnia, A. (2017). Meta-Analysis of Transcriptome Regulation During Induction to Cardiac Myocyte Fate From Mouse and Human Fibroblasts. *Journal of Cellular Physiology*, *232*(8), 2053–2062. https://doi.org/10.1002/jcp.25580

Rathbone, J., Franklin, R., Gibbs, C., & Williams, D. (2017). Review article: Role of magnesium sulphate in the management of Irukandji syndrome: A systematic review. *Emergency Medicine Australasia : EMA*, *29*(1), 9–17. https://doi.org/10.1111/1742-6723.12694

Rebuzzini, P., Zuccotti, M., & Garagna, S. (2021). Building pluripotency identity in the early embryo and derived stem cells. *Cells*, *10*(8). Scopus. https://doi.org/10.3390/cells10082049

Respuela, P., & Rada-Iglesias, A. (2016). Enhancer Remodeling During Early Mammalian Embryogenesis: Lessons for Somatic Reprogramming, Rejuvenation, and Aging. *Current Stem Cell Reports*, *2*(3), 263–272. Scopus. https://doi.org/10.1007/s40778-016-0050-8

Rojczyk, E., Klama-Baryła, A., Łabuś, W., Wilemska-Kucharzewska, K., & Kucharzewski, M. (2020). Historical and modern research on propolis and its application in wound healing and other fields of medicine and contributions by Polish studies. *Journal of Ethnopharmacology*, *262*, 113159. https://doi.org/10.1016/j.jep.2020.113159

Saigusa, T., Aono, Y., & Waddington, J. (2017). Mechanisms underlying delta- and mu-opioid receptor agonist-induced increases in extracellular dopamine level in the nucleus accumbens of freely moving rats. *JOURNAL OF ORAL SCIENCE*, *59*(2), 195–200. https://doi.org/10.2334/josnusd.16-0874

Sakaguchi, W., To, M., Yamamoto, Y., Inaba, K., Yakeishi, M., Saruta, J., Fuchida, S., Hamada, N., & Tsukinoki, K. (2019). Detection of anti-citrullinated protein antibody (ACPA) in saliva for rheumatoid arthritis using DBA mice infected with Porphyromonas gingivalis. *ARCHIVES OF ORAL BIOLOGY*, *108*. https://doi.org/10.1016/j.archoralbio.2019.104510

Salhi, L., Rompen, E., Sakalihasan, N., Laleman, I., Teughels, W., Michel, J.-B., & Lambert, F. (2019). Can Periodontitis Influence the Progression of Abdominal Aortic Aneurysm? A Systematic Review. *Angiology*, *70*(6), 479–491. https://doi.org/10.1177/0003319718821243

Sanematsu, K., Shigemura, N., & Ninomiya, Y. (2017). Binding properties between human sweet receptor and sweet-inhibitor, gymnemic acids. *JOURNAL OF ORAL BIOSCIENCES*, *59*(3), 127–130. https://doi.org/10.1016/j.job.2017.05.004

Schuch, L. F., Silveira, F. M., Wagner, V. P., Borgato, G. B., Rocha, G. Z., Castilho, R. M., Vargas, P. A., & Martins, M. D. (2020). Head and neck cancer patient-derived xenograft models—A systematic review. *Critical Reviews in Oncology/Hematology*, *155*, 103087. https://doi.org/10.1016/j.critrevonc.2020.103087

Semi, K., & Takashima, Y. (2021). Pluripotent stem cells for the study of early human embryology. *Development Growth and Differentiation*, *63*(2), 104–115. Scopus. https://doi.org/10.1111/dgd.12715

Serra, R., Andreucci, M., De Caridi, G., Massara, M., Mastroroberto, P., & de Franciscis, S. (2017). Functional chronic venous disease: A systematic review. *Phlebology*, *32*(9), 588–592. https://doi.org/10.1177/0268355516686451

Shanbhag, S., Pandis, N., Mustafa, K., Nyengaard, J. R., & Stavropoulos, A. (2017). Cell Cotransplantation Strategies for Vascularized Craniofacial Bone Tissue Engineering: A Systematic Review and Meta-Analysis of Preclinical In Vivo Studies. *Tissue Engineering. Part B, Reviews*, *23*(2), 101–117. https://doi.org/10.1089/ten.TEB.2016.0283

Shanbhag, S., Stavropoulos, A., Suliman, S., Hervig, T., & Mustafa, K. (2017). Efficacy of Humanized Mesenchymal Stem Cell Cultures for Bone Tissue Engineering: A Systematic Review with a Focus on Platelet Derivatives. *Tissue Engineering. Part B, Reviews*, *23*(6), 552–569. https://doi.org/10.1089/ten.TEB.2017.0093

Shao, J., Kolwijck, E., Jansen, J. A., Yang, F., & Walboomers, X. F. (2017). Animal models for percutaneous-device-related infections: A review. *International Journal of Antimicrobial Agents*, *49*(6), 659–667. Scopus. https://doi.org/10.1016/j.ijantimicag.2017.01.022

Sorial, A. K., Anjum, S. A., Cook, M. J., Board, T. N., & O’Neill, T. W. (2020). Statins, bone biology and revision arthroplasty: Review of clinical and experimental evidence. *Therapeutic Advances in Musculoskeletal Disease*, *12*. Scopus. https://doi.org/10.1177/1759720X20966229

Spriet, M., Willcox, J. L., & Culp, W. T. N. (2019). Role of Positron Emission Tomography in Imaging of Non-neurologic Disorders of the Head, Neck, and Teeth in Veterinary Medicine. *Frontiers in Veterinary Science*, *6*(JUN). Scopus. https://doi.org/10.3389/fvets.2019.00180

Stepniewska, K., Humphreys, G. S., Gonçalves, B. P., Craig, E., Gosling, R., Guerin, P. J., Price, R. N., Barnes, K. I., Raman, J., Smit, M. R., D’Alessandro, U., Stone, W. J. R., Bjorkman, A., Samuels, A. M., Arroyo-Arroyo, M. I., Bastiaens, G. J. H., Brown, J. M., Dicko, A., El-Sayed, B. B., … Bousema, T. (2022). Efficacy of Single-Dose Primaquine With Artemisinin Combination Therapy on Plasmodium falciparum Gametocytes and Transmission: An Individual Patient Meta-Analysis. *The Journal of Infectious Diseases*, *225*(7), 1215–1226. https://doi.org/10.1093/infdis/jiaa498

Stracke, K., Jex, A. R., & Traub, R. J. (2020). Zoonotic Ancylostomiasis: An Update of a Continually Neglected Zoonosis. *The American Journal of Tropical Medicine and Hygiene*, *103*(1), 64–68. https://doi.org/10.4269/ajtmh.20-0060

Suzawa, T., Yoshida, H., Takahashi, M., Itose, M., Takimoto, R., Sasama, Y., Tanaka, M., Ikezaki, K., Shirora, T., Maki, K., & Kamijo, R. (2020). Prospects of neural crest-derived cells from oral and dentofacial tissues for application in regenerative medicine. *ORAL SCIENCE INTERNATIONAL*, *17*(3), 115–125. https://doi.org/10.1002/osi2.1064

Suzuki, A., Abdallah, N., Gajera, M., Jun, G., Jia, P., Zhao, Z., & Iwata, J. (2018). Genes and microRNAs associated with mouse cleft palate: A systematic review and bioinformatics analysis. *Mechanisms of Development*, *150*, 21–27. https://doi.org/10.1016/j.mod.2018.02.003

Suzuki, Y. (2013). Chaperone therapy update: Fabry disease, GM1-gangliosidosis and Gaucher disease. *Brain and Development*, *35*(6), 515–523. Scopus. https://doi.org/10.1016/j.braindev.2012.12.002

Sysoeva, O. V., Smirnov, K., & Stroganova, T. A. (2020). Sensory evoked potentials in patients with Rett syndrome through the lens of animal studies: Systematic review. *Clinical Neurophysiology : Official Journal of the International Federation of Clinical Neurophysiology*, *131*(1), 213–224. https://doi.org/10.1016/j.clinph.2019.11.003

Tan, S. F., Tong, H. J., Lin, X. Y., Mok, B., & Hong, C. H. (2016). The cariogenicity of commercial infant formulas: A systematic review. *European Archives of Paediatric Dentistry : Official Journal of the European Academy of Paediatric Dentistry*, *17*(3), 145–156. https://doi.org/10.1007/s40368-016-0228-x

Tan, S. S. H., Tjio, C. K. E., Wong, J. R. Y., Wong, K. L., Chew, J. R. J., Hui, J. H. P., & Toh, W. S. (2021). Mesenchymal Stem Cell Exosomes for Cartilage Regeneration: A Systematic Review of Preclinical In Vivo Studies. *Tissue Engineering. Part B, Reviews*, *27*(1), 1–13. https://doi.org/10.1089/ten.TEB.2019.0326

Tanaka, Y., Fukumoto, S., & Sugawara, S. (2019). Mechanisms underlying the induction of regulatory T cells by sublingual immunotherapy. *JOURNAL OF ORAL BIOSCIENCES*, *61*(2), 73–77. https://doi.org/10.1016/j.job.2019.02.001

Tang, Z.-Y., Li, Y., Tang, Y.-T., Ma, X.-D., & Tang, Z.-Y. (2022). Anticancer activity of oleanolic acid and its derivatives: Recent advances in evidence, target profiling and mechanisms of action. *Biomedicine & Pharmacotherapy = Biomedecine & Pharmacotherapie*, *145*, 112397. https://doi.org/10.1016/j.biopha.2021.112397

Tavares, S. J. S., & Lima, V. (2021). Bone anti-resorptive effects of coumarins on RANKL downstream cellular signaling: A systematic review of the literature. *Fitoterapia*, *150*, 104842. https://doi.org/10.1016/j.fitote.2021.104842

Tee, B. C., & Sun, Z. (2015). Mandibular distraction osteogenesis assisted by cell-based tissue engineering: A systematic review. *Orthodontics & Craniofacial Research*, *18 Suppl 1*(0 1), 39–49. https://doi.org/10.1111/ocr.12087

Thanigaimani, S., & Golledge, J. (2021). Role of Adipokines and Perivascular Adipose Tissue in Abdominal Aortic Aneurysm: A Systematic Review and Meta-Analysis of Animal and Human Observational Studies. *Frontiers in Endocrinology*, *12*, 618434. https://doi.org/10.3389/fendo.2021.618434

Thanigaimani, S., Phie, J., & Golledge, J. (2020). Animal models of ischemic limb ulcers: A systematic review and meta-analysis. *BMJ Open Diabetes Research & Care*, *8*(1). https://doi.org/10.1136/bmjdrc-2020-001676

Toyoda, H. (2019). Role of nicotinic acetylcholine receptors for modulation of microcircuits in the agranular insular cortex. *JOURNAL OF ORAL BIOSCIENCES*, *61*(1), 5–11. https://doi.org/10.1016/j.job.2018.12.001

Tracey, E. F., McDermott, R. A., & McDonald, M. I. (2016). Do worms protect against the metabolic syndrome? A systematic review and meta-analysis. *Diabetes Research and Clinical Practice*, *120*, 209–220. https://doi.org/10.1016/j.diabres.2016.08.014

Ulbrich, S. E., Groebner, A. E., & Bauersachs, S. (2013). Transcriptional profiling to address molecular determinants of endometrial receptivity—Lessons from studies in livestock species. *Methods*, *59*(1), 108–115. Scopus. https://doi.org/10.1016/j.ymeth.2012.10.013

Vaidyanathan, K., & Gopalakrishnan, S. (2017). Nanomedicine in the Diagnosis and Treatment of Atherosclerosis-A Systematic Review. *Cardiovascular & Hematological Disorders Drug Targets*, *17*(2), 119–131. https://doi.org/10.2174/1871529X17666170918142653

Valeria, C., Carmine, S., Valentina, M., Teresa, I., Maria, C., Martina, T., Giancarlo, A., Giovanna, N., Graziamaria, C., & Amelia, F. (2021). The need of a multicomponent guiding approach to personalize clopidogrel treatment. *The Pharmacogenomics Journal*, *21*(2), 116–127. https://doi.org/10.1038/s41397-020-00189-2

Vandooren, J., Van Den Steen, P. E., & Opdenakker, G. (2013). Biochemistry and molecular biology of gelatinase B or matrix metalloproteinase-9 (MMP-9): The next decade. *Critical Reviews in Biochemistry and Molecular Biology*, *48*(3), 222–272. Scopus. https://doi.org/10.3109/10409238.2013.770819

Vrhovac Madunić, I., Karin-Kujundžić, V., Madunić, J., Šola, I. M., & Šerman, L. (2021). Endometrial Glucose Transporters in Health and Disease. *Frontiers in Cell and Developmental Biology*, *9*. Scopus. https://doi.org/10.3389/fcell.2021.703671

Watts, V., Attie, M., & McClure, S. (2019). Reconstruction of Complex Full-Thickness Scalp Defects After Dog-Bite Injuries Using Dermal Regeneration Template (Integra): Case Report and Literature Review. *JOURNAL OF ORAL AND MAXILLOFACIAL SURGERY*, *77*(2), 338–351. https://doi.org/10.1016/j.joms.2018.08.022

Williams, A. L., Bates, C. A., Pace, N. D., Leonhard, M. J., Chang, E. T., & DeSesso, J. M. (2018). Impact of chloroform exposures on reproductive and developmental outcomes: A systematic review of the scientific literature. *Birth Defects Research*, *110*(17), 1267–1313. https://doi.org/10.1002/bdr2.1382

Williams, A. M., Zent, C. S., & Janelsins, M. C. (2016). What is known and unknown about chemotherapy-related cognitive impairment in patients with haematological malignancies and areas of needed research. *British Journal of Haematology*, *174*(6), 835–846. https://doi.org/10.1111/bjh.14211

Winterhager, E., & Kidder, G. M. (2015). Gap junction connexins in female reproductive organs: Implications for women’s reproductive health. *Human Reproduction Update*, *21*(3), 340–352. https://doi.org/10.1093/humupd/dmv007

Xu, J., Gong, T., Heng, B. C., & Zhang, C. F. (2017). A systematic review: Differentiation of stem cells into functional pericytes. *FASEB Journal : Official Publication of the Federation of American Societies for Experimental Biology*, *31*(5), 1775–1786. https://doi.org/10.1096/fj.201600951RRR

Xu, S., Kamato, D., Little, P. J., Nakagawa, S., Pelisek, J., & Jin, Z. G. (2019). Targeting epigenetics and non-coding RNAs in atherosclerosis: From mechanisms to therapeutics. *Pharmacology & Therapeutics*, *196*, 15–43. https://doi.org/10.1016/j.pharmthera.2018.11.003

Yamawaki, Y., Oue, K., Shirawachi, S., Asano, S., Harada, K., & Kanematsu, T. (2017). Phospholipase C-related catalytically inactive protein can regulate obesity, a state of peripheral inflammation. *JAPANESE DENTAL SCIENCE REVIEW*, *53*(1), 18–24. https://doi.org/10.1016/j.jdsr.2016.06.001

Ye, Y., Jensen, D., Viet, C., Pan, H., Campana, W., Amit, M., & Boada, M. (n.d.). Advances in Head and Neck Cancer Pain. *JOURNAL OF DENTAL RESEARCH*. https://doi.org/10.1177/00220345221088527

Yeung, A. W. K., El-Demerdash, A., Berindan-Neagoe, I., Atanasov, A. G., & Ho, Y.-S. (2018). Molecular Responses of Cancers by Natural Products: Modifications of Autophagy Revealed by Literature Analysis. *Critical Reviews in Oncogenesis*, *23*(5–6), 347–370. https://doi.org/10.1615/CritRevOncog.2018027566

Yoshida, A., Moritani, M., Nagase, Y., & Bae, Y. (2017). Projection and synaptic connectivity of trigeminal mesencephalic nucleus neurons controlling jaw reflexes. *JOURNAL OF ORAL SCIENCE*, *59*(2), 177–182. https://doi.org/10.2334/josnusd.16-0845

Zakis, D. R., Paulissen, E., Kornete, L., Kaan, A. M. M., Nicu, E. A., & Zaura, E. (2022). The evidence for placental microbiome and its composition in healthy pregnancies: A systematic review. *Journal of Reproductive Immunology*, *149*, 103455. https://doi.org/10.1016/j.jri.2021.103455

Zhang, L., Zhang, W., Jin, H., Wang, D., Wei, N., & Wang, Y. (2016). Effect and safety of Shengxuening (extract from excrement of bombyxin) for renal anemia: A systematic review. *Journal of Traditional Chinese Medicine = Chung i Tsa Chih Ying Wen Pan*, *36*(5), 588–595. https://doi.org/10.1016/s0254-6272(16)30077-2

Ziecik, A., Waclawik, A., Kaczmarek, M., Blitek, A., Jalali, B. M., & Andronowska, A. (2011). Mechanisms for the Establishment of Pregnancy in the Pig. *Reproduction in Domestic Animals*, *46*(SUPPL. 3), 31–41. Scopus. https://doi.org/10.1111/j.1439-0531.2011.01843.x

No pair-wise meta-analysis

Hao, C.-P., Cao, N.-J., Zhu, Y.-H., & Wang, W. (2021). The osseointegration and stability of dental implants with different surface treatments in animal models: A network meta-analysis. *Scientific Reports*, *11*(1), 13849. https://doi.org/10.1038/s41598-021-93307-4

Khijmatgar, S., Panda, S., Das, M., Arbildo-Vega, H., & Del Fabbro, M. (2021). Recombinant factors for periodontal intrabony defects: A systematic review and network meta-analysis of preclinical studies. *Journal of Tissue Engineering and Regenerative Medicine*, *15*(12), 1069–1081. https://doi.org/10.1002/term.3250

Zhang, S., Zhang, X., Li, Y., Mao, X., Liu, R., Qi, Y., Lee, E.-S., & Jiang, H. B. (2022). Clinical Reference Strategy for the Selection of Treatment Materials for Maxillofacial Bone Transplantation: A Systematic Review and Network Meta-Analysis. *Tissue Engineering and Regenerative Medicine*, *19*(3), 437–450. https://doi.org/10.1007/s13770-022-00445-5

Methodological study

Cardoso, M., Catré, D., Noites, R., Paulo, M., & Viegas, C. (2018). Animal models used in furcation perforation studies: A systematic review and comprehensive synthesis of model characteristics. *Australian Endodontic Journal*, *44*(3), 273–280. Scopus. https://doi.org/10.1111/aej.12221

de Carvalho, M. F. F., Leijôto-Lannes, A. C. N., Rodrigues, M. C. N. de, Nogueira, L. C., Ferraz, N. K. L., Moreira, A. N., Yamauti, M., Zina, L. G., & Magalhães, C. S. de. (2018). Viability of Bovine Teeth as a Substrate in Bond Strength Tests: A Systematic Review and Meta-analysis. *The Journal of Adhesive Dentistry*, *20*(6), 471–479. https://doi.org/10.3290/j.jad.a41636

Glösel, B., Kuchler, U., Watzek, G., & Gruber, R. (2010). Review of dental implant rat research models simulating osteoporosis or diabetes. *The International Journal of Oral & Maxillofacial Implants*, *25*(3), 516–524.

Gritsch, K., Laroche, N., Morgon, L., Al-Hity, R., Vico, L., Colon, P., & Grosgogeat, B. (2012). A systematic review of methods for tissue analysis in animal studies on orthodontic mini-implants. *Orthodontics & Craniofacial Research*, *15*(3), 135–147. https://doi.org/10.1111/j.1601-6343.2012.01548.x

Hariyani, N., Halimah, A., Al-Junaid, M., Fadhila, O., & Budhy, T. (2021). Mouse periodontitis models using whole Porphyromonas gingivalis bacteria induction. *SAUDI DENTAL JOURNAL*, *33*(8), 819–825. https://doi.org/10.1016/j.sdentj.2021.08.001

Mangione, F., Salmon, B., EzEldeen, M., Jacobs, R., Chaussain, C., & Vital, S. (2022). Characteristics of Large Animal Models for Current Cell-Based Oral Tissue Regeneration. *Tissue Engineering. Part B, Reviews*, *28*(3), 489–505. https://doi.org/10.1089/ten.TEB.2020.0384

Tabatabaei, F. S., Tatari, S., Samadi, R., & Moharamzadeh, K. (2016). Different methods of dentin processing for application in bone tissue engineering: A systematic review. *Journal of Biomedical Materials Research. Part A*, *104*(10), 2616–2627. https://doi.org/10.1002/jbm.a.35790

Vajgel, A., Mardas, N., Farias, B., Petrie, A., Cimoes, R., & Donos, N. (2014). A systematic review on the critical size defect model. *CLINICAL ORAL IMPLANTS RESEARCH*, *25*(8), 879–893. https://doi.org/10.1111/clr.12194

**Excluded after full-text assessment**

No (living) animals

Ahmed, R., & Mulder, R. (2021). A systematic review on the efficacy of vaporized hydrogen peroxide as a non-contact decontamination system for pathogens associated with the dental environment. *International Journal of Environmental Research and Public Health*, *18*(9). Scopus. https://doi.org/10.3390/ijerph18094748

Alenazy, M. S., & Mosadomi, H. A. (2014). Clinical implications of calcifying nanoparticles in dental diseases: A critical review. *International Journal of Nanomedicine*, *9*, 27–31. https://doi.org/10.2147/IJN.S51538

Annunziata, M., Nastri, L., Cecoro, G., & Guida, L. (2017). The Use of Poly-d,l-lactic Acid (PDLLA) Devices for Bone Augmentation Techniques: A Systematic Review. *Molecules (Basel, Switzerland)*, *22*(12). https://doi.org/10.3390/molecules22122214

Bordea, I. R., Candrea, S., Alexescu, G. T., Bran, S., Băciuț, M., Băciuț, G., Lucaciu, O., Dinu, C. M., & Todea, D. A. (2020). Nano-hydroxyapatite use in dentistry: A systematic review. *Drug Metabolism Reviews*, *52*(2), 319–332. https://doi.org/10.1080/03602532.2020.1758713

Cagetti, M., Bonta, G., Cocco, F., Lingstrom, P., Strohmenger, L., & Campus, G. (2018). Are standardized caries risk assessment models effective in assessing actual caries status and future caries increment? A systematic review. *BMC ORAL HEALTH*, *18*. https://doi.org/10.1186/s12903-018-0585-4

Canellas, J. V. D. S., Fraga, S. R. G., Santoro, M. F., Netto, J. de N. S., & Tinoco, E. M. B. (2020). Intrasocket interventions to prevent alveolar osteitis after mandibular third molar surgery: A systematic review and network meta-analysis. *Journal of Cranio-Maxillo-Facial Surgery : Official Publication of the European Association for Cranio-Maxillo-Facial Surgery*, *48*(9), 902–913. https://doi.org/10.1016/j.jcms.2020.06.012

Casale, M., Moffa, A., Vella, P., Sabatino, L., Capuano, F., Salvinelli, B., Lopez, M. A., Carinci, F., & Salvinelli, F. (2016). Hyaluronic acid: Perspectives in dentistry. A systematic review. *International Journal of Immunopathology and Pharmacology*, *29*(4), 572–582. https://doi.org/10.1177/0394632016652906

Chaudhry, K., Khatana, S., Dutt, N., Mittal, Y., Sharma, S., & Elhence, P. (2019). Systematic Review of Lesser Known Parasitoses: Maxillofacial Dirofilariasis. *JOURNAL OF MAXILLOFACIAL & ORAL SURGERY*, *18*(2), 180–189. https://doi.org/10.1007/s12663-018-1139-7

Choi, I., Cortes, A., Arita, E., & Georgetti, M. (2018). Comparison of conventional imaging techniques and CBCT for periodontal evaluation: A systematic review. *IMAGING SCIENCE IN DENTISTRY*, *48*(2), 79–86. https://doi.org/10.5624/isd.2018.48.2.79

Corcuera-Flores, J.-R., Casttellanos-Cosano, L., Torres-Lagares, D., Serrera- Figallo, M. Á., Rodríguez-Caballero, Á. N. G. E. L. A., & Machuca-Portillo, G. (2016). A systematic review of the oral and craniofacial manifestations of cri du chat syndrome. *Clinical Anatomy*, *29*(5), 555–560. Scopus. https://doi.org/10.1002/ca.22654

Cortela, D. C. B., de Souza Junior, A. L., Virmond, M. C. L., & Ignotti, E. (2015). Inflammatory Mediators of Leprosy Reactional Episodes and Dental Infections: A Systematic Review. *Mediators of Inflammation*, *2015*, 548540. https://doi.org/10.1155/2015/548540

Cuevas-González, M. V., Suaste-Olmos, F., García-Calderón, A. G., Tovar-Carrillo, K. L., Espinosa-Cristóbal, L. F., Nava-Martínez, S. D., Cuevas-González, J. C., Zambrano-Galván, G., Saucedo-Acuña, R. A., & Donohue-Cornejo, A. (2021). Expression of MicroRNAs in Periodontal Disease: A Systematic Review. *BioMed Research International*, *2021*, 2069410. https://doi.org/10.1155/2021/2069410

Dan, A. E. B., Thygesen, T. H., & Pinholt, E. M. (2010). Corticosteroid administration in oral and orthognathic surgery: A systematic review of the literature and meta-analysis. *Journal of Oral and Maxillofacial Surgery : Official Journal of the American Association of Oral and Maxillofacial Surgeons*, *68*(9), 2207–2220. https://doi.org/10.1016/j.joms.2010.04.019

de França, G. M., Pinheiro, J. C., de Melo Fernandes Almeida, D. R., da Silva, G. G., de Lima, K. C., de Andrade Santos, P. P., & Galvão, H. C. (2021). Analysis of Protein Immunoexpression and Its Interrelationship in the Pathogenesis of Odontomas and Ameloblastic Fibro-Odontomas: A Systematic Review. *Head and Neck Pathology*, *15*(3), 955–966. https://doi.org/10.1007/s12105-020-01260-x

De Roo, N. M. C., Thierens, L. A. M., Temmerman, L., & De Pauw, G. A. M. (2018). The evaluation of the transport medium for extracted premolars prior to cryopreservation: A systematic literature review. *Cell and Tissue Banking*, *19*(3), 259–267. https://doi.org/10.1007/s10561-018-9690-5

Derruau, S., Robinet, J., Untereiner, V., Piot, O., Sockalingum, G. D., & Lorimier, S. (2020). Vibrational Spectroscopy Saliva Profiling as Biometric Tool for Disease Diagnostics: A Systematic Literature. *Molecules (Basel, Switzerland)*, *25*(18). https://doi.org/10.3390/molecules25184142

Elad, S., Epstein, J. B., Yarom, N., Drucker, S., Tzach, R., & von Bültzingslöwen, I. (2010). Topical immunomodulators for management of oral mucosal conditions, a systematic review; part I: calcineurin inhibitors. *Expert Opinion on Emerging Drugs*, *15*(4), 713–726. https://doi.org/10.1517/14728214.2010.528389

Fagundes, N. C. F., Bittencourt, L. O., Magno, M. B., Marques, M. M., Maia, L. C., & Lima, R. R. (2018). Efficacy of Hank’s balanced salt solution compared to other solutions in the preservation of the periodontal ligament. A systematic review and meta-analysis. *PloS One*, *13*(7), e0200467. https://doi.org/10.1371/journal.pone.0200467

Fiorino, A., Marturano, A., Placella, G., Staderini, E., Domingo, L. I., Cerulli, G. G., Tiribuzi, R., & Blasi, P. (2021). Amelogenin-Derived Peptides in Bone Regeneration: A Systematic Review. *International Journal of Molecular Sciences*, *22*(17). https://doi.org/10.3390/ijms22179224

Fokas, G., Vaughn, V. M., Scarfe, W. C., & Bornstein, M. M. (2018). Accuracy of linear measurements on CBCT images related to presurgical implant treatment planning: A systematic review. *Clinical Oral Implants Research*, *29 Suppl 16*, 393–415. https://doi.org/10.1111/clr.13142

Gandhi, V., Mehta, S., Gauthier, M., Mu, J., Kuo, C., Nanda, R., & Yadav, S. (2021). Comparison of external apical root resorption with clear aligners and pre-adjusted edgewise appliances in non-extraction cases: A systematic review and meta-analysis. *EUROPEAN JOURNAL OF ORTHODONTICS*, *43*(1), 15–24. https://doi.org/10.1093/ejo/cjaa013

Granate-Marques, A., Polis-Yanes, C., Seminario-Amez, M., Jané-Salas, E., & López-López, J. (2019). Medication-related osteonecrosis of the jaw associated with implant and regenerative treatments: Systematic review. *Medicina Oral, Patologia Oral y Cirugia Bucal*, *24*(2), e195–e203. https://doi.org/10.4317/medoral.22691

Gu, M., Zhang, Y., Liu, H., Liu, J., Zhu, D., & Yang, X. (2018). MSH homeobox 1 polymorphisms and the risk of non-syndromic orofacial clefts: A meta-analysis. *European Journal of Oral Sciences*, *126*(3), 180–185. https://doi.org/10.1111/eos.12414

Halimi, A., Benyahia, H., Bahije, L., Adli, H., Azeroual, M.-F., & Zaoui, F. (2016). A systematic study of the release of bisphenol A by orthodontic materials and its biological effects. *International Orthodontics*, *14*(4), 399–417. https://doi.org/10.1016/j.ortho.2016.10.005

Hong, D.-W., Lin, X.-J., Wiegand, A., & Yu, H. (2020). Does delayed toothbrushing after the consumption of erosive foodstuffs or beverages decrease erosive tooth wear? A systematic review and meta-analysis. *Clinical Oral Investigations*, *24*(12), 4169–4183. https://doi.org/10.1007/s00784-020-03614-9

Hsu, Y.-T., Fu, J.-H., Al-Hezaimi, K., & Wang, H.-L. (2012). Biomechanical implant treatment complications: A systematic review of clinical studies of implants with at least 1 year of functional loading. *The International Journal of Oral & Maxillofacial Implants*, *27*(4), 894–904.

Ittichaicharoen, J., Chattipakorn, N., & Chattipakorn, S. (2016). Is salivary gland function altered in noninsulin-dependent diabetes mellitus and obesity-insulin resistance? *ARCHIVES OF ORAL BIOLOGY*, *64*, 61–71. https://doi.org/10.1016/j.archoralbio.2016.01.002

Lang, L. A., & Tulunoglu, I. (2014). A critically appraised topic review of computer-aided design/computer-aided machining of removable partial denture frameworks. *Dental Clinics of North America*, *58*(1), 247–255. Scopus. https://doi.org/10.1016/j.cden.2013.09.006

Lissek, M., Boeker, M., & Happe, A. (2020). How Thick Is the Oral Mucosa around Implants after Augmentation with Different Materials: A Systematic Review of the Effectiveness of Substitute Matrices in Comparison to Connective Tissue Grafts. *International Journal of Molecular Sciences*, *21*(14). https://doi.org/10.3390/ijms21145043

Louropoulou, A., Slot, D. E., & Van der Weijden, F. A. (2012). Titanium surface alterations following the use of different mechanical instruments: A systematic review. *Clinical Oral Implants Research*, *23*(6), 643–658. https://doi.org/10.1111/j.1600-0501.2011.02208.x

Maisonneuve, P., Amar, S., & Lowenfels, A. B. (2017). Periodontal disease, edentulism, and pancreatic cancer: A meta-analysis. *Annals of Oncology : Official Journal of the European Society for Medical Oncology*, *28*(5), 985–995. https://doi.org/10.1093/annonc/mdx019

Meursinge Reynders, R., Ronchi, L., Ladu, L., Van Etten-Jamaludin, F., & Bipat, S. (2013). Insertion torque and orthodontic mini-implants: A systematic review of the artificial bone literature. *Proceedings of the Institution of Mechanical Engineers. Part H, Journal of Engineering in Medicine*, *227*(11), 1181–1202. https://doi.org/10.1177/0954411913495986

Mickenautsch, S., Yengopal, V., & Banerjee, A. (2010). Pulp response to resin-modified glass ionomer and calcium hydroxide cements in deep cavities: A quantitative systematic review. *Dental Materials : Official Publication of the Academy of Dental Materials*, *26*(8), 761–770. https://doi.org/10.1016/j.dental.2010.03.021

Möhlhenrich, S. C., Modabber, A., Steiner, T., Mitchell, D. A., & Hölzle, F. (2015). Heat generation and drill wear during dental implant site preparation: Systematic review. *The British Journal of Oral & Maxillofacial Surgery*, *53*(8), 679–689. https://doi.org/10.1016/j.bjoms.2015.05.004

Molina-García, A., Castellanos-Cosano, L., Machuca-Portillo, G., & Posada-de la Paz, M. (2016). Impact of rare diseases in oral health. *Medicina Oral Patologia Oral y Cirugia Bucal*, *21*(5), e587–e594. Scopus. https://doi.org/10.4317/medoral.20972

Moussa, M., Goldsmith, M., & Komarova, S. (n.d.). Craniofacial Bones and Teeth in Spacefarers: Systematic Review and Meta-analysis. *JDR CLINICAL & TRANSLATIONAL RESEARCH*. https://doi.org/10.1177/23800844221084985

Nawrot-Hadzik, I., Matkowski, A., Hadzik, J., Dobrowolska-Czopor, B., Olchowy, C., Dominiak, M., & Kubasiewicz-Ross, P. (2021). Proanthocyanidins and Flavan-3-Ols in the Prevention and Treatment of Periodontitis-Antibacterial Effects. *Nutrients*, *13*(1). https://doi.org/10.3390/nu13010165

Nguyen, K.-C. T., Pachêco-Pereira, C., Kaipatur, N. R., Cheung, J., Major, P. W., & Le, L. H. (2018). Comparison of ultrasound imaging and cone-beam computed tomography for examination of the alveolar bone level: A systematic review. *PloS One*, *13*(10), e0200596. https://doi.org/10.1371/journal.pone.0200596

Nicolielo, L., Jacobs, R., Albdour, E., Hoste, X., Abeloos, J., Politis, C., & Swennen, G. (2017). Is oestrogen associated with mandibular condylar resorption? A systematic review. *INTERNATIONAL JOURNAL OF ORAL AND MAXILLOFACIAL SURGERY*, *46*(11), 1394–1402. https://doi.org/10.1016/j.ijom.2017.06.012

Revanth, M. P., Aparna, S., & Madankumar, P. D. (2020). Effects of mobile phone radiation on buccal mucosal cells: A systematic review. *Electromagnetic Biology and Medicine*, *39*(4), 273–281. https://doi.org/10.1080/15368378.2020.1793168

Rickert, D., Slater, J. J. R. H., Meijer, H. J. A., Vissink, A., & Raghoebar, G. M. (2012). Maxillary sinus lift with solely autogenous bone compared to a combination of autogenous bone and growth factors or (solely) bone substitutes. A systematic review. *International Journal of Oral and Maxillofacial Surgery*, *41*(2), 160–167. Scopus. https://doi.org/10.1016/j.ijom.2011.10.001

Rossini, G., Parrini, S., Castroflorio, T., Deregibus, A., & Debernardi, C. (2015a). Periodontal health during clear aligners treatment: A systematic review. *EUROPEAN JOURNAL OF ORTHODONTICS*, *37*(5), 539–543. https://doi.org/10.1093/ejo/cju083

Rossini, G., Parrini, S., Castroflorio, T., Deregibus, A., & Debernardi, C. L. (2015b). Efficacy of clear aligners in controlling orthodontic tooth movement: A systematic review. *Angle Orthodontist*, *85*(5), 881–889. Scopus. https://doi.org/10.2319/061614-436.1

Saatchi, M., Shokraneh, A., Navaei, H., Maracy, M. R., & Shojaei, H. (2014). Antibacterial effect of calcium hydroxide combined with chlorhexidine on Enterococcus faecalis: A systematic review and meta-analysis. *Journal of Applied Oral Science : Revista FOB*, *22*(5), 356–365. https://doi.org/10.1590/1678-775720140032

Sabour, A., El Helou, M., Roger-Leroi, V., & Bauer, C. (2021). Release and toxicity of bisphenol-A (BPA) contained in orthodontic adhesives: A systematic review. *International Orthodontics*, *19*(1), 1–14. https://doi.org/10.1016/j.ortho.2020.11.002

Sahrmann, P., Attin, T., & Schmidlin, P. (2011). Regenerative Treatment of Peri-Implantitis Using Bone Substitutes and Membrane: A Systematic Review. *CLINICAL IMPLANT DENTISTRY AND RELATED RESEARCH*, *13*(1), 46–57. https://doi.org/10.1111/j.1708-8208.2009.00183.x

Shaikh, M. S., Zafar, M. S., & Alnazzawi, A. (2021). Comparing Nanohydroxyapatite Graft and Other Bone Grafts in the Repair of Periodontal Infrabony Lesions: A Systematic Review and Meta-Analysis. *International Journal of Molecular Sciences*, *22*(21). https://doi.org/10.3390/ijms222112021

Sharma, V. K., Shukla, N. K., Chaturvedi, T. P., & Singh, S. (2021). Variables to predict spontaneous eruption of palatally displaced permanent canine after interceptive extraction of primary canine: A systematic review and meta-analysis. *International Orthodontics*, *19*(1), 25–36. https://doi.org/10.1016/j.ortho.2020.11.004

Siew, K., Lee, A., & Cheung, G. (2015). Treatment Outcome of Repaired Root Perforation: A Systematic Review and Meta-analysis. *JOURNAL OF ENDODONTICS*, *41*(11), 1795–1804. https://doi.org/10.1016/j.joen.2015.07.007

Šimundić Munitić, M., Poklepović Peričić, T., Utrobičić, A., Bago, I., & Puljak, L. (2019). Antimicrobial efficacy of commercially available endodontic bioceramic root canal sealers: A systematic review. *PloS One*, *14*(10), e0223575. https://doi.org/10.1371/journal.pone.0223575

Skupien, J. A., Sarkis-Onofre, R., Cenci, M. S., Moraes, R. R. de, & Pereira-Cenci, T. (2015). A systematic review of factors associated with the retention of glass fiber posts. *Brazilian Oral Research*, *29*, S1806-83242015000100400. https://doi.org/10.1590/1807-3107BOR-2015.vol29.0074

Soares, F. Z. M., Follak, A., da Rosa, L. S., Montagner, A. F., Lenzi, T. L., & Rocha, R. O. (2016). Bovine tooth is a substitute for human tooth on bond strength studies: A systematic review and meta-analysis of in vitro studies. *Dental Materials : Official Publication of the Academy of Dental Materials*, *32*(11), 1385–1393. https://doi.org/10.1016/j.dental.2016.09.019

Spin-Neto, R., Gotfredsen, E., & Wenzel, A. (2013). Impact of voxel size variation on CBCT-based diagnostic outcome in dentistry: A systematic review. *Journal of Digital Imaging*, *26*(4), 813–820. https://doi.org/10.1007/s10278-012-9562-7

Stavropoulos, A., & Wikesjo, U. (2012). Growth and differentiation factors for periodontal regeneration: A review on factors with clinical testing. *JOURNAL OF PERIODONTAL RESEARCH*, *47*(5), 545–553. https://doi.org/10.1111/j.1600-0765.2012.01478.x

Villa, A., Wolff, A., Aframian, D., Vissink, A., Ekstrom, J., Proctor, G., McGowan, R., Narayana, N., Aliko, A., Sia, Y., Joshi, R., Jensen, S., Kerr, A., Dawes, C., & Pedersen, A. (2015). World Workshop on Oral Medicine VI: a systematic review of medication-induced salivary gland dysfunction: Prevalence, diagnosis, and treatment. *CLINICAL ORAL INVESTIGATIONS*, *19*(7), 1563–1580. https://doi.org/10.1007/s00784-015-1488-2

Yassen, G. H., & Platt, J. A. (2013). The effect of nonsetting calcium hydroxide on root fracture and mechanical properties of radicular dentine: A systematic review. *International Endodontic Journal*, *46*(2), 112–118. https://doi.org/10.1111/j.1365-2591.2012.02121.x

Yeung, C. A., Chong, L. Y., & Glenny, A.-M. (2015). Fluoridated milk for preventing dental caries. *The Cochrane Database of Systematic Reviews*, *8*, CD003876. https://doi.org/10.1002/14651858.CD003876.pub3

Zamri, F., & de Vries, T. J. (2020). Use of TNF Inhibitors in Rheumatoid Arthritis and Implications for the Periodontal Status: For the Benefit of Both? *Frontiers in Immunology*, *11*, 591365. https://doi.org/10.3389/fimmu.2020.591365

Zemouri, C., Jakubovics, N. S., Crielaard, W., Zaura, E., Dodds, M., Schelkle, B., & Loos, B. G. (2019). Resistance and resilience to experimental gingivitis: A systematic scoping review. *BMC Oral Health*, *19*(1), 212. https://doi.org/10.1186/s12903-019-0889-z

Zhao, H., Hu, J., & Zhao, L. (2020). Histological analysis of socket preservation using DBBM. A systematic review and meta-analysis. *Journal of Stomatology, Oral and Maxillofacial Surgery*, *121*(6), 729–735. https://doi.org/10.1016/j.jormas.2020.04.011

Zhou, P., Xie, Y., Yan, Z., Liu, X., & Hua, H. (2019). Association between dectin-1 gene single nucleotide polymorphisms and fungal infection: A systemic review and meta-analysis. *Bioscience Reports*, *39*(11). https://doi.org/10.1042/BSR20191519

No systematic review

Abbott, P. (2022). Present status and future directions: Managing endodontic emergencies. *INTERNATIONAL ENDODONTIC JOURNAL*, *55*, 778–803. https://doi.org/10.1111/iej.13678

Agossa, K., Dendooven, A., Dubuquoy, L., Gower-Rousseau, C., Delcourt-Debruyne, E., & Capron, M. (2017). Periodontal manifestations of inflammatory bowel disease: Emerging epidemiologic and biologic evidence. *JOURNAL OF PERIODONTAL RESEARCH*, *52*(3), 313–324. https://doi.org/10.1111/jre.12422

Aksel, H., & Serper, A. (2014). Recent considerations in regenerative endodontic treatment approaches. *JOURNAL OF DENTAL SCIENCES*, *9*(3), 207–213. https://doi.org/10.1016/j.jds.2013.12.007

Al-Shammery, D., Michelogiannakis, D., Rossouw, E., Romanos, G. E., & Javed, F. (2019). Influence of psychological stress exposure on orthodontic therapy: A comprehensive review. *Journal of Investigative and Clinical Dentistry*, *10*(2), e12388. Scopus. https://doi.org/10.1111/jicd.12388

Ayuthaya, B., Everts, V., & Pavasant, P. (2018). The immunopathogenic and immunomodulatory effects of interleukin-12 in periodontal disease. *EUROPEAN JOURNAL OF ORAL SCIENCES*, *126*(2), 75–83. https://doi.org/10.1111/eos.12405

Badillo-Perona, V., Cano-Sanchez, J., Campo-Trapero, J., & Bascones-Martinez, A. (2011). Peri-implant bone mechanobiology. Review of the literature. *MEDICINA ORAL PATOLOGIA ORAL Y CIRUGIA BUCAL*, *16*(5), E677–E681. https://doi.org/10.4317/medoral.17053

Bimstein, E., & Rotstein, I. (2016). Cvek pulpotomy—Revisited. *DENTAL TRAUMATOLOGY*, *32*(6), 438–442. https://doi.org/10.1111/edt.12297

D’Apuzzo, F., Cappabianca, S., Ciavarella, D., Monsurrò, A., Silvestrini-Biavati, A., & Perillo, L. (2013). Biomarkers of periodontal tissue remodeling during orthodontic tooth movement in mice and men: Overview and clinical relevance. *The Scientific World Journal*, *2013*. Scopus. https://doi.org/10.1155/2013/105873

Davidson, A. P., Gregory, C., & Dedrick, P. (2014). Successful management permitting delayed operative revision of cleft palate in a labrador retriever. *Veterinary Clinics of North America - Small Animal Practice*, *44*(2), 325–329. Scopus. https://doi.org/10.1016/j.cvsm.2013.11.002

El-Awady, A., Elashiry, M., Morandini, A., Meghil, M., & Cutler, C. (2022). Dendritic cells a critical link to alveolar bone loss and systemic disease risk in periodontitis: Immunotherapeutic implications. *PERIODONTOLOGY 2000*, *89*(1), 41–50. https://doi.org/10.1111/prd.12428

Fard, S., Khanghahi, A., Moghaddam, B., Ahmadian, A., & Ebrahimi, A. (2018). APPLICATION OF NANO COMPOSITES IN CALVARIA HEALING AND BONE DEFECTS: A LITERATURE OF REVIEW. *ANNALS OF DENTAL SPECIALTY*, *6*(1), 77–82.

Ferguson, D., Vaid, N., & Wilcko, M. (2018). Assessing accelerated tooth movement techniques on their own catabolic merits: A review. *JOURNAL OF THE WORLD FEDERATION OF ORTHODONTISTS*, *7*(4), 122–127. https://doi.org/10.1016/j.ejwf.2018.11.003

Firkova, E. I., & Chaprazov, T. S. (2021). In vivo animal models in periodontal research-focus on rodents. *Bulgarian Journal of Veterinary Medicine*, *24*(2), 167–175. Scopus. https://doi.org/10.15547/bjvm.2019-0056

Fujita, Y. (2018). Impact of a high-fat diet on bone health during growth. *PEDIATRIC DENTAL JOURNAL*, *28*(1), 1–6. https://doi.org/10.1016/j.pdj.2017.11.003

Fulton, A. J., Fiani, N., & Verstraete, F. J. M. (2014). Canine pediatric dentistry. *Veterinary Clinics of North America - Small Animal Practice*, *44*(2), 303–324. Scopus. https://doi.org/10.1016/j.cvsm.2013.11.004

Gasik, M., Braem, A., Chaudhari, A., Duyck, J., & Vleugels, J. (2015). Titanium implants with modified surfaces: Meta-analysis of in vivo osteointegration. *Materials Science & Engineering. C, Materials for Biological Applications*, *49*, 152–158. https://doi.org/10.1016/j.msec.2014.12.074

Goldschmidt, S. (2022). Surgical Margins for Ameloblastoma in Dogs: A Review With an Emphasis on the Future. *Frontiers in Veterinary Science*, *9*. Scopus. https://doi.org/10.3389/fvets.2022.830258

Goldwaser, B., Papadaki, M., Kaban, L., & Troulis, M. (2012). Automated Continuous Mandibular Distraction Osteogenesis: Review of the Literature. *JOURNAL OF ORAL AND MAXILLOFACIAL SURGERY*, *70*(2), 407–416. https://doi.org/10.1016/j.joms.2011.01.042

Greenstein, G., & Cavallaro, J. (2013). Managing the buccal gap and plate of bone: Immediate dental implant placement. *Dentistry Today*, *32*(3), 70, 72–77; quiz 78–79. Scopus.

Habelitz, S., & Bai, Y. (2021). Mechanisms of Enamel Mineralization Guided by Amelogenin Nanoribbons. *JOURNAL OF DENTAL RESEARCH*, *100*(13), 1434–1443. https://doi.org/10.1177/00220345211012925

He, Y., Bao, W., Wu, X.-D., Huang, W., Chen, H., & Li, Z. (2019). Effects of Systemic or Local Administration of Zoledronate on Implant Osseointegration: A Preclinical Meta-Analysis. *BioMed Research International*, *2019*, 9541485. https://doi.org/10.1155/2019/9541485

Hernandez, M., Phulpin, B., Mansuy, L., & Droz, D. (2017). Use of new targeted cancer therapies in children: Effects on dental development and risk of jaw osteonecrosis: A review. *JOURNAL OF ORAL PATHOLOGY & MEDICINE*, *46*(5), 321–326. https://doi.org/10.1111/jop.12516

Hosoya, N., Takigawa, T., Horie, T., Maeda, H., Yamamoto, Y., Momoi, Y., Yamamoto, K., & Okiji, T. (2019). A review of the literature on the efficacy of mineral trioxide aggregate in conservative dentistry. *DENTAL MATERIALS JOURNAL*, *38*(5), 693–700. https://doi.org/10.4012/dmj.2018-193

Iwata, J. (2021). Gene-environment interplay and MicroRNAs in cleft lip and cleft palate. *ORAL SCIENCE INTERNATIONAL*, *18*(1), 3–13. https://doi.org/10.1002/osi2.1072

Juriga, S., & Bilyard, K. (2021). Working Dog Dentistry. *Veterinary Clinics of North America - Small Animal Practice*, *51*(4), 779–802. Scopus. https://doi.org/10.1016/j.cvsm.2021.04.002

Khojasteh, A., Behnia, H., Dashti, S., & Stevens, M. (2012). Current Trends in Mesenchymal Stem Cell Application in Bone Augmentation: A Review of the Literature. *JOURNAL OF ORAL AND MAXILLOFACIAL SURGERY*, *70*(4), 972–982. https://doi.org/10.1016/j.joms.2011.02.133

Ko, F. C., & Sumner, D. R. (2021). How faithfully does intramembranous bone regeneration recapitulate embryonic skeletal development? *Developmental Dynamics*, *250*(3), 377–392. Scopus. https://doi.org/10.1002/dvdy.240

Kressirer, C., Smith, D., King, W., Dobeck, J., Starr, J., & Tanner, A. (2017). Scardovia wiggsiae and its potential role as a caries pathogen. *JOURNAL OF ORAL BIOSCIENCES*, *59*(3), 135–141. https://doi.org/10.1016/j.job.2017.05.002

Liu, H., Jiang, H., & Wang, Y. (2013). The biological effects of occlusal trauma on the stomatognathic system—A focus on animal studies. *JOURNAL OF ORAL REHABILITATION*, *40*(2), 130–138. https://doi.org/10.1111/joor.12017

Martins, O., Ramos, J. C., Baptista, I. P., & Dard, M. M. (2014). The dog as a model for peri-implantitis. A review. *Journal of Investigative Surgery*, *27*(1), 50–56. Scopus. https://doi.org/10.3109/08941939.2013.828805

Miron, R. J., Guillemette, V., Zhang, Y., Chandad, F., & Sculean, A. (2014). Enamel matrix derivative in combination with bone grafts: A review of the literature. *Quintessence International (Berlin, Germany : 1985)*, *45*(6), 475–487. https://doi.org/10.3290/j.qi.a31541

Moreno-Hidalgo, M., Caleza-Jimenez, C., Mendoza-Mendoza, A., & Iglesias-Linares, A. (2014). Revascularization of immature permanent teeth with apical periodontitis. *INTERNATIONAL ENDODONTIC JOURNAL*, *47*(4), 321–331. https://doi.org/10.1111/iej.12154

Najeeb, S., Khurshid, Z., Zaffar, M. S., Zohaib, S., & Siddiqui, F. (2017). Efficacy of enamel matrix derivative in vital pulp therapy: A review of literature. *Iranian Endodontic Journal*, *12*(3), 269–275. Scopus. https://doi.org/10.22037/iej.v12i3.12036

Naka, S., & Matsumoto-Nakano, M. (2021). Non-alcoholic steatohepatitis caused by oral bacteria. *PEDIATRIC DENTAL JOURNAL*, *31*(1), 11–16. https://doi.org/10.1016/j.pdj.2020.12.004

Nastri, L., Moretti, A., Migliaccio, S., Paoletta, M., Annunziata, M., Liguori, S., Toro, G., Bianco, M., Cecoro, G., Guida, L., & Iolascon, G. (2020). Do Dietary Supplements and Nutraceuticals Have Effects on Dental Implant Osseointegration? A Scoping Review. *Nutrients*, *12*(1). https://doi.org/10.3390/nu12010268

Neto, A., Maia, S., Leao, J., Quidute, I., Guimaraes, C., Alves, S., Alvares, P., Ribeiro, M., & Silva, L. (2021). Mechanisms Involved in Apice Closure of Pulpless Teeth—Literature Review. *OPEN DENTISTRY JOURNAL*, *15*, 127–136. https://doi.org/10.2174/1874210602115010127

Nguyen, W., Grigori, L., Just, E., Santos, C., & Seleem, D. (2021). The in vivo anti-Candida albicans activity of flavonoids. *JOURNAL OF ORAL BIOSCIENCES*, *63*(2), 120–128. https://doi.org/10.1016/j.job.2021.03.004

Niibe, K., Zhang, M., Nakazawa, K., Morikawa, S., Nakagawa, T., Matsuzaki, Y., & Egusa, H. (2017). The potential of enriched mesenchymal stem cells with neural crest cell phenotypes as a cell source for regenerative dentistry. *Japanese Dental Science Review*, *53*(2), 25–33. Scopus. https://doi.org/10.1016/j.jdsr.2016.09.001

Ozkurt, Z., & Kazazoglu, E. (2011). Zirconia Dental Implants: A Literature Review. *JOURNAL OF ORAL IMPLANTOLOGY*, *37*(3), 367–376. https://doi.org/10.1563/AAID-JOI-D-09-00079

Parirokh, M., & Torabinejad, M. (2010). Mineral Trioxide Aggregate: A Comprehensive Literature Review-Part III: Clinical Applications, Drawbacks, and Mechanism of Action. *JOURNAL OF ENDODONTICS*, *36*(3), 400–413. https://doi.org/10.1016/j.joen.2009.09.009

Park, K. O., Lee, J. H., Park, J. H., Shin, Y. C., Huh, J. B., Bae, J.-H., Kang, S. H., Hong, S. W., Kim, B., Yang, D. J., Han, D.-W., & Yeum, J. H. (2016). Graphene oxide-coated guided bone regeneration membranes with enhanced osteogenesis: Spectroscopic analysis and animal study. *Applied Spectroscopy Reviews*, *51*(7–9), 540–551. Scopus. https://doi.org/10.1080/05704928.2016.1165687

Pera, M. F., & Rossant, J. (2021). The exploration of pluripotency space: Charting cell state transitions in peri-implantation development. *Cell Stem Cell*, *28*(11), 1896–1906. Scopus. https://doi.org/10.1016/j.stem.2021.10.001

Permuy, M., López-Peña, M., González-Cantalapiedra, A., & Muñoz, F. (2017). Melatonin: A Review of Its Potential Functions and Effects on Dental Diseases. *International Journal of Molecular Sciences*, *18*(4). https://doi.org/10.3390/ijms18040865

Rossi-Fedele, G., Guastalli, A., Dogramaci, E., Steier, L., & De Figueiredo, J. (2011). Influence of pH changes on chlorine-containing endodontic irrigating solutions. *INTERNATIONAL ENDODONTIC JOURNAL*, *44*(9), 792–799. https://doi.org/10.1111/j.1365-2591.2011.01911.x

Salvi, G., Cosgarea, R., & Sculean, A. (2017). Prevalence and Mechanisms of Peri-implant Diseases. *JOURNAL OF DENTAL RESEARCH*, *96*(1), 31–37. https://doi.org/10.1177/0022034516667484

Schwitalla, A., & Müller, W.-D. (2013). PEEK dental implants: A review of the literature. *Journal of Oral Implantology*, *39*(6), 743–749. Scopus. https://doi.org/10.1563/AAID-JOI-D-11-00002

Sciorsci, R. L., Lillo, E., Occhiogrosso, L., & Rizzo, A. (2020). Ozone therapy in veterinary medicine: A review. *Research in Veterinary Science*, *130*, 240–246. Scopus. https://doi.org/10.1016/j.rvsc.2020.03.026

Şenel, S. (2021). An overview of physical, microbiological and immune barriers of oral mucosa. *International Journal of Molecular Sciences*, *22*(15). Scopus. https://doi.org/10.3390/ijms22157821

Sharma, M., Fonseca, F., Hunter, K., & Radhakrishnan, R. (2020). Loss of oral mucosal stem cell markers in oral submucous fibrosis and their reactivation in malignant transformation. *INTERNATIONAL JOURNAL OF ORAL SCIENCE*, *12*(1). https://doi.org/10.1038/s41368-020-00090-5

Tanaka, J., & Mishima, K. (2021). Application of regenerative medicine to salivary gland hypofunction. *JAPANESE DENTAL SCIENCE REVIEW*, *57*, 54–59. https://doi.org/10.1016/j.jdsr.2021.03.002

Tanaka, J., Takamatsu, K., Yukimori, A., Kujiraoka, S., Ishida, S., Takakura, I., Yasuhara, R., & Mishima, K. (2021). Sox9 function in salivary gland development. *JOURNAL OF ORAL BIOSCIENCES*, *63*(1), 8–13. https://doi.org/10.1016/j.job.2021.01.005

Tang, E., & Arany, P. (2013). Photobiomodulation and implants: Implications for dentistry. *JOURNAL OF PERIODONTAL AND IMPLANT SCIENCE*, *43*(6), 262–268. https://doi.org/10.5051/jpis.2013.43.6.262

Thalji, G., & Cooper, L. (2013). Molecular Assessment of Osseointegration In Vivo: A Review of the Current Literature. *INTERNATIONAL JOURNAL OF ORAL & MAXILLOFACIAL IMPLANTS*, *28*(6), E521–E534. https://doi.org/10.11607/jomi.te33

Tziafas, D. (2019). Characterization of Odontoblast-like Cell Phenotype and Reparative Dentin Formation In Vivo: A Comprehensive Literature Review. *JOURNAL OF ENDODONTICS*, *45*(3), 241–249. https://doi.org/10.1016/j.joen.2018.12.002

Vina-Almunia, J., Candel-Marti, M., Cervera-Ballester, J., Garcia-Mira, B., Calvo-Guirado, J., Penarrocha-Oltra, D., & Penarrocha-Diago, M. (2013). Buccal Bone Crest Dynamics After Immediate Implant Placement and Ridge Preservation Techniques: Review of Morphometric Studies in Animals. *IMPLANT DENTISTRY*, *22*(2), 155–160. https://doi.org/10.1097/ID.0b013e318287a947

Wingo, K. (2018). A review of dental cements. *Journal of Veterinary Dentistry*, *35*(1), 18–27. Scopus. https://doi.org/10.1177/0898756418755339

Yamakoshi, Y., Chiba-Ohkuma, R., Hidaka, Y., Onuma, K., Yamamoto, R., Saito, M., & Karakida, T. (2022). Repurposing MDZ as a tool for tissue regeneration in dental cells. *JOURNAL OF ORAL BIOSCIENCES*, *64*(1), 37–42. https://doi.org/10.1016/j.job.2021.10.005

Yaseen, H. S., Asif, M., Saadullah, M., Mahrukh, Asghar, S., Shams, M. U., Bazmi, R. R., Saleem, M., Yousaf, H. M., & Yaseen, M. (2020). Methanolic extract of Ephedra ciliata promotes wound healing and arrests inflammatory cascade in vivo through downregulation of TNF-α. *Inflammopharmacology*, *28*(6), 1691–1704. https://doi.org/10.1007/s10787-020-00713-7

Zacher, A., & Marretta, S. M. (2022). Diagnosis and Management of Furcation Lesions in Dogs – A Review. *Journal of Veterinary Dentistry*, *39*(2), 151–172. Scopus. https://doi.org/10.1177/08987564221076908

No dentistry topic

Bhagat, S., Agarwal, M., & Roy, V. (2013). Serratiopeptidase: A systematic review of the existing evidence. *International Journal of Surgery (London, England)*, *11*(3), 209–217. https://doi.org/10.1016/j.ijsu.2013.01.010

De Ceulaer, J., De Clercq, C., & Swennen, G. R. J. (2012). Robotic surgery in oral and maxillofacial, craniofacial and head and neck surgery: A systematic review of the literature. *International Journal of Oral and Maxillofacial Surgery*, *41*(11), 1311–1324. https://doi.org/10.1016/j.ijom.2012.05.035

Delgado-Ruiz, R. A., Calvo-Guirado, J. L., & Romanos, G. E. (2015). Critical size defects for bone regeneration experiments in rabbit calvariae: Systematic review and quality evaluation using ARRIVE guidelines. *Clinical Oral Implants Research*, *26*(8), 915–930. https://doi.org/10.1111/clr.12406

Fliefel, R., Ehrenfeld, M., & Otto, S. (2018). Induced pluripotent stem cells (iPSCs) as a new source of bone in reconstructive surgery: A systematic review and meta-analysis of preclinical studies. *Journal of Tissue Engineering and Regenerative Medicine*, *12*(7), 1780–1797. https://doi.org/10.1002/term.2697

Muhammad, S. A., Nordin, N., & Fakurazi, S. (2018). Regenerative potential of secretome from dental stem cells: A systematic review of preclinical studies. *Reviews in the Neurosciences*, *29*(3), 321–332. Scopus. https://doi.org/10.1515/revneuro-2017-0069

Ortiz, A. de C., Fideles, S. O. M., Pomini, K. T., Reis, C. H. B., Bueno, C. R. de S., Pereira, E. de S. B. M., Rossi, J. de O., Novais, P. C., Pilon, J. P. G., Rosa Junior, G. M., Buchaim, D. V., & Buchaim, R. L. (2021). Effects of Therapy with Fibrin Glue combined with Mesenchymal Stem Cells (MSCs) on Bone Regeneration: A Systematic Review. *Cells*, *10*(9). https://doi.org/10.3390/cells10092323

Price, K., Haddad, Y., & Fakhouri, W. (2016). Analysis of the Relationship Between Micrognathia and Cleft Palate: A Systematic Review. *CLEFT PALATE-CRANIOFACIAL JOURNAL*, *53*(2), E34–E44. https://doi.org/10.1597/14-238

Ribeiro, J. C. V., Vieira, R. S., Melo, I. M., Araújo, V. M. A., & Lima, V. (2017). Versatility of Chitosan-Based Biomaterials and Their Use as Scaffolds for Tissue Regeneration. *TheScientificWorldJournal*, *2017*, 8639898. https://doi.org/10.1155/2017/8639898

Wan, Z., Zhang, P., Lv, L., & Zhou, Y. (2020). NIR light-assisted phototherapies for bone-related diseases and bone tissue regeneration: A systematic review. *Theranostics*, *10*(25), 11837–11861. https://doi.org/10.7150/thno.49784

No English full-text

Valery, M., Antonov, I. I., Nelyubin, V. N., Muraev, A. A., & Ivanov, S. Yu. (2021). CURRENT OPPORTUNITIES and PROSPECTIVES of IMMUNOTROPIC THERAPY in CHRONIC GENERALIZED PERIODONTITIS. *Medical Immunology (Russia)*, *23*(5), 1055–1068. Scopus. https://doi.org/10.15789/1563-0625-COA-2156

No full-text access

Goldschmidt, S., & Hoyer, N. (2022). Management of Dental and Oral Developmental Conditions in Dogs and Cats. *Veterinary Clinics of North America - Small Animal Practice*, *52*(1), 139–158. Scopus. https://doi.org/10.1016/j.cvsm.2021.09.002
