# Supplementary material for: Methodological quality of systematic reviews in dentistry including animal studies: a cross-sectional study
Source: Ir Vet J. 2023 Dec 14;76:33. doi: 10.1186/s13620-023-00261-w (PMC10720166; doi:10.1186/s13620-023-00261-w)
Supplement: Supplementary file 4 — Additional file 4. Included articles. [file 13620_2023_261_MOESM4_ESM.docx]

Supplementary file 4 – Included articles

Aarthi, J., Muthu, M. S., & Sujatha, S. (2013). Cariogenic potential of milk and infant formulas: A systematic review. *European Archives of Paediatric Dentistry : Official Journal of the European Academy of Paediatric Dentistry*, *14*(5), 289–300. https://doi.org/10.1007/s40368-013-0088-6

Abduljabbar, T., Kellesarian, S. V., Vohra, F., Akram, Z., Kotsakis, G. A., Yunker, M., Romanos, G. E., & Javed, F. (2017). Effect of Growth Hormone Supplementation on Osseointegration: A Systematic Review and Meta-analyses. *Implant Dentistry*, *26*(4), 613–620. https://doi.org/10.1097/ID.0000000000000616

Abduo, J., & Judge, R. B. (2014). Implications of implant framework misfit: A systematic review of biomechanical sequelae. *The International Journal of Oral & Maxillofacial Implants*, *29*(3), 608–621. https://doi.org/10.11607/jomi.3418

Agnihotri, R., & Gaur, S. (2021). Applications of teriparatide for alveolar bone regeneration: A systematic review. *JOURNAL OF INTERNATIONAL SOCIETY OF PREVENTIVE AND COMMUNITY DENTISTRY*, *11*(6), 639–643. https://doi.org/10.4103/jispcd.JISPCD_169_21

Al Amri, M. D. (2016). Crestal bone loss around submerged and nonsubmerged dental implants: A systematic review. *The Journal of Prosthetic Dentistry*, *115*(5), 564-570.e1. https://doi.org/10.1016/j.prosdent.2015.11.002

Alaluusua, S. (2010). Aetiology of Molar-Incisor Hypomineralisation: A systematic review. *European Archives of Paediatric Dentistry : Official Journal of the European Academy of Paediatric Dentistry*, *11*(2), 53–58. https://doi.org/10.1007/BF03262713

Alberton Nuernberg, M. A., Janjacomo Miessi, D. M., Ivanaga, C. A., Bocalon Olivo, M., Ervolino, E., Gouveia Garcia, V., Wainwright, M., & Theodoro, L. H. (2019). Influence of antimicrobial photodynamic therapy as an adjunctive to scaling and root planing on alveolar bone loss: A systematic review and meta-analysis of animal studies. *Photodiagnosis and Photodynamic Therapy*, *25*, 354–363. https://doi.org/10.1016/j.pdpdt.2019.01.020

Alenezi, A., Chrcanovic, B., & Wennerberg, A. (2018). Effects of Local Drug and Chemical Compound Delivery on Bone Regeneration Around Dental Implants in Animal Models: A Systematic Review and Meta-Analysis. *The International Journal of Oral & Maxillofacial Implants*, *33*(1), e1–e18. https://doi.org/10.11607/jomi.6333

Alghamdi, F. T., & Alqurashi, A. E. (2020). Regenerative Endodontic Therapy in the Management of Immature Necrotic Permanent Dentition: A Systematic Review. *TheScientificWorldJournal*, *2020*, 7954357. https://doi.org/10.1155/2020/7954357

Al-Hezaimi, K., Javed, F., Al-Fouzan, K., & Tay, F. (2013). Efficacy of the enamel matrix derivative in direct pulp capping procedures: A systematic review. *Australian Endodontic Journal : The Journal of the Australian Society of Endodontology Inc*, *39*(3), 171–175. https://doi.org/10.1111/j.1747-4477.2012.00357.x

Almidfa, N. S. S., Athanasiou, A. E., Makrygiannakis, M. A., & Kaklamanos, E. G. (2021). Does the rate of orthodontic tooth movement change during the estrus cycle? A systematic review based on animal studies. *BMC Oral Health*, *21*(1), 526. https://doi.org/10.1186/s12903-021-01875-8

Altaii, M., Richards, L., & Rossi-Fedele, G. (2017). Histological assessment of regenerative endodontic treatment in animal studies with different scaffolds: A systematic review. *DENTAL TRAUMATOLOGY*, *33*(4), 235–244. https://doi.org/10.1111/edt.12338

Al-Thomali, Y., Basha, S., & Mohamed, R. N. (2022). Effect of surface treatment on the mechanical stability of orthodontic miniscrews. *The Angle Orthodontist*, *92*(1), 127–136. https://doi.org/10.2319/020721-111.1

Alvarez-Camino, J., Valmaseda-Castellon, E., & Gay-Escoda, C. (2013). Immediate implants placed in fresh sockets associated to periapical infectious processes. A systematic review. *MEDICINA ORAL PATOLOGIA ORAL Y CIRUGIA BUCAL*, *18*(5), E780–E785. https://doi.org/10.4317/medoral.18942

Alves, M. J., Baratieri, C., Mattos, C. T., Araújo, M. T. de S., & Maia, L. C. (2013). Root repair after contact with mini-implants: Systematic review of the literature. *European Journal of Orthodontics*, *35*(4), 491–499. https://doi.org/10.1093/ejo/cjs025

Arora, H., & Ivanovski, S. (2017). Melatonin as a pro-osteogenic agent in oral implantology: A systematic review of histomorphometric outcomes in animals and quality evaluation using ARRIVE guidelines. *Journal of Periodontal Research*, *52*(2), 151–161. https://doi.org/10.1111/jre.12386

Asa’ad, F., Garaicoa-Pazmiño, C., Dahlin, C., & Larsson, L. (2020). Expression of MicroRNAs in Periodontal and Peri-Implant Diseases: A Systematic Review and Meta-Analysis. *International Journal of Molecular Sciences*, *21*(11). https://doi.org/10.3390/ijms21114147

Azuma, M. M., Cardoso, C. D. B. M., da Silva, C. C., de Oliveira, P. H. C., Jacinto, R. D. C., Andrada, A. C., & Cintra, L. T. A. (2022). The use of omega-3 fatty acids in the treatment of oral diseases. *Oral Diseases*, *28*(2), 264–274. Scopus. https://doi.org/10.1111/odi.13667

Azuma, M. M., Samuel, R. O., Gomes-Filho, J. E., Dezan-Junior, E., & Cintra, L. T. A. (2014). The role of IL-6 on apical periodontitis: A systematic review. *International Endodontic Journal*, *47*(7), 615–621. https://doi.org/10.1111/iej.12196

Bassetti, M. A., Bassetti, R. G., & Bosshardt, D. D. (2016). The alveolar ridge splitting/expansion technique: A systematic review. *Clinical Oral Implants Research*, *27*(3), 310–324. https://doi.org/10.1111/clr.12537

Bastami, F., Nazeman, P., Moslemi, H., Rezai Rad, M., Sharifi, K., & Khojasteh, A. (2017). Induced pluripotent stem cells as a new getaway for bone tissue engineering: A systematic review. *Cell Proliferation*, *50*(2). https://doi.org/10.1111/cpr.12321

Basudan, A. M., Shaheen, M. Y., de Vries, R. B., van den Beucken, J. J. J. P., Jansen, J. A., & Alghamdi, H. S. (2019). Antiosteoporotic Drugs to Promote Bone Regeneration Related to Titanium Implants: A Systematic Review and Meta-Analysis. *Tissue Engineering. Part B, Reviews*, *25*(2), 89–99. https://doi.org/10.1089/ten.TEB.2018.0120

Benetti, F., Lemos, C. A. A., de Oliveira Gallinari, M., Terayama, A. M., Briso, A. L. F., de Castilho Jacinto, R., Sivieri-Araújo, G., & Cintra, L. T. A. (2018). Influence of different types of light on the response of the pulp tissue in dental bleaching: A systematic review. *Clinical Oral Investigations*, *22*(4), 1825–1837. https://doi.org/10.1007/s00784-017-2278-9

Berardini, M., Trisi, P., Sinjari, B., Rutjes, A. W. S., & Caputi, S. (2016). The Effects of High Insertion Torque Versus Low Insertion Torque on Marginal Bone Resorption and Implant Failure Rates: A Systematic Review With Meta-Analyses. *Implant Dentistry*, *25*(4), 532–540. https://doi.org/10.1097/ID.0000000000000422

Bernabeu-Mira, J. C., Soto-Peñaloza, D., Peñarrocha-Diago, M., Camacho-Alonso, F., Rivas-Ballester, R., & Peñarrocha-Oltra, D. (2021). Low-speed drilling without irrigation versus conventional drilling for dental implant osteotomy preparation: A systematic review. *Clinical Oral Investigations*, *25*(7), 4251–4267. https://doi.org/10.1007/s00784-021-03939-z

Bertl, K., Steiner, I., Pandis, N., Buhlin, K., Klinge, B., & Stavropoulos, A. (2018). Statins in nonsurgical and surgical periodontal therapy. A systematic review and meta-analysis of preclinical in vivo trials. *Journal of Periodontal Research*, *53*(3), 267–287. https://doi.org/10.1111/jre.12514

Betti, B. F., Everts, V., Ket, J. C. F., Tabeian, H., Bakker, A. D., Langenbach, G. E., & Lobbezoo, F. (2018). Effect of mechanical loading on the metabolic activity of cells in the temporomandibular joint: A systematic review. *Clinical Oral Investigations*, *22*(1), 57–67. https://doi.org/10.1007/s00784-017-2189-9

Blázquez-Hinarejos, M., Ayuso-Montero, R., Jané-Salas, E., & López-López, J. (2017). Influence of surface modified dental implant abutments on connective tissue attachment: A systematic review. *Archives of Oral Biology*, *80*, 185–192. https://doi.org/10.1016/j.archoralbio.2017.04.020

Bral, A., & Mommaerts, M. Y. (2016). In vivo biofunctionalization of titanium patient-specific implants with nano hydroxyapatite and other nano calcium phosphate coatings: A systematic review. *Journal of Cranio-Maxillo-Facial Surgery : Official Publication of the European Association for Cranio-Maxillo-Facial Surgery*, *44*(4), 400–412. https://doi.org/10.1016/j.jcms.2015.12.004

Brandini, D. A., Debortoli, C. V. L., Felipe Akabane, S. T., Poi, W. R., & Amaral, M. F. (2018). Systematic review of the effects of excessive occlusal mechanical load on the periodontum of rats. *Indian Journal of Dental Research : Official Publication of Indian Society for Dental Research*, *29*(6), 812–819. https://doi.org/10.4103/ijdr.IJDR_31_17

Bright, R., Hynes, K., Gronthos, S., & Bartold, P. M. (2015). Periodontal ligament-derived cells for periodontal regeneration in animal models: A systematic review. *Journal of Periodontal Research*, *50*(2), 160–172. https://doi.org/10.1111/jre.12205

Bunte, K., Hensel, A., & Beikler, T. (2019). Polyphenols in the prevention and treatment of periodontal disease: A systematic review of in vivo, ex vivo and in vitro studies. *Fitoterapia*, *132*, 30–39. https://doi.org/10.1016/j.fitote.2018.11.012

Cadenas-Perula, M., Yañez-Vico, R. M., Solano-Reina, E., & Iglesias-Linares, A. (2016). Effectiveness of biologic methods of inhibiting orthodontic tooth movement in animal studies. *American Journal of Orthodontics and Dentofacial Orthopedics : Official Publication of the American Association of Orthodontists, Its Constituent Societies, and the American Board of Orthodontics*, *150*(1), 33–48. https://doi.org/10.1016/j.ajodo.2016.01.015

Cafferata, E. A., Jerez, A., Vernal, R., Monasterio, G., Pandis, N., & Faggion, C. M. J. (2019). The therapeutic potential of regulatory T lymphocytes in periodontitis: A systematic review. *Journal of Periodontal Research*, *54*(3), 207–217. https://doi.org/10.1111/jre.12629

Călin, C., Sajin, M., Moldovan, V. T., Coman, C., Stratul, S. I., & Didilescu, A. C. (2021). Immunohistochemical expression of non-collagenous extracellular matrix molecules involved in tertiary dentinogenesis following direct pulp capping: A systematic review. *Annals of Anatomy = Anatomischer Anzeiger : Official Organ of the Anatomische Gesellschaft*, *235*, 151674. https://doi.org/10.1016/j.aanat.2020.151674

Carvalho-Lobato, P., Garcia, V. J., Kasem, K., Ustrell-Torrent, J. M., Tallón-Walton, V., & Manzanares-Céspedes, M. C. (2014). Tooth movement in orthodontic treatment with low-level laser therapy: A systematic review of human and animal studies. *Photomedicine and Laser Surgery*, *32*(5), 302–309. https://doi.org/10.1089/pho.2012.3439

Chew, J. R. J., Tan, B. L., Lu, J. X., Tong, H. J., & Duggal, M. S. (2022). Cell-Based Therapy for Tooth Replantation Following Avulsion: A Systematic Review. *Tissue Engineering. Part B, Reviews*, *28*(2), 351–363. https://doi.org/10.1089/ten.TEB.2021.0016

Choowong, P., Wali, J. A., Nguyen, A. T. M., Jayasinghe, T. N., & Eberhard, J. (2022). Macronutrient-induced modulation of periodontitis in rodents-a systematic review. *Nutrition Reviews*, *80*(5), 1160–1178. https://doi.org/10.1093/nutrit/nuab048

Chrcanovic, B., Martins, M., & Wennerberg, A. (2015). Immediate Placement of Implants into Infected Sites: A Systematic Review. *CLINICAL IMPLANT DENTISTRY AND RELATED RESEARCH*, *17*, E1–E16. https://doi.org/10.1111/cid.12098

Corrêa, A. S., Almeida, V. L. D., Lopes, B. M. V., Franco, A., Matos, F. R. D., Quintans-Júnior, L. J., Rode, S. M., & Paranhos, L. R. (2017). The influence of non-steroidal anti-inflammatory drugs and paracetamol used for pain control of orthodontic tooth movement: A systematic review. *Anais Da Academia Brasileira de Ciencias*, *89*(4), 2851–2863. https://doi.org/10.1590/0001-3765201720160865

Costa, M. J. F., de Araújo, I. D. T., da Rocha Alves, L., da Silva, R. L., Dos Santos Calderon, P., Borges, B. C. D., de Aquino Martins, A. R. L., de Vasconcelos Gurgel, B. C., & Lins, R. D. A. U. (2021). Relationship of Porphyromonas gingivalis and Alzheimer’s disease: A systematic review of pre-clinical studies. *Clinical Oral Investigations*, *25*(3), 797–806. https://doi.org/10.1007/s00784-020-03764-w

Cotti, E., Ideo, F., Pedrazzini, A., Bardini, G., Musu, D., & Kantarci, A. (2021). Proresolving Mediators in Endodontics: A Systematic Review. *Journal of Endodontics*, *47*(5), 711–720. https://doi.org/10.1016/j.joen.2021.01.008

Crossman, J., Elyasi, M., El-Bialy, T., & Flores Mir, C. (2018). Cementum regeneration using stem cells in the dog model: A systematic review. *Archives of Oral Biology*, *91*, 78–90. https://doi.org/10.1016/j.archoralbio.2018.04.001

Cruz, R., Pesce, G., Calasans-Maia, J., Moraschini, V., Calasans-Maia, M. D., & Granjeiro, J. M. (2020). Calcium Phosphate Carrying Simvastatin Enhances Bone Regeneration: A Systematic Review. *Brazilian Dental Journal*, *31*(2), 93–102. https://doi.org/10.1590/0103-6440202002971

Cunha, W., Carvalho, O., Henriques, B., Silva, F. S., Özcan, M., & Souza, J. C. M. (2022). Surface modification of zirconia dental implants by laser texturing. *Lasers in Medical Science*, *37*(1), 77–93. Scopus. https://doi.org/10.1007/s10103-021-03475-y

da Costa, G. de F. A., Souza, R. D. C., de Araújo, G. M., Gurgel, B. C. de V., Barbosa, G. A. S., & Calderon, P. D. S. (2017). Does TGF-β play a role in degenerative temporomandibular joint diseases? A systematic review. *Cranio : The Journal of Craniomandibular Practice*, *35*(4), 228–232. https://doi.org/10.1080/08869634.2016.1193961

da Rosa, W. L. O., Cocco, A. R., Silva, T. M. da, Mesquita, L. C., Galarça, A. D., Silva, A. F. da, & Piva, E. (2018). Current trends and future perspectives of dental pulp capping materials: A systematic review. *Journal of Biomedical Materials Research. Part B, Applied Biomaterials*, *106*(3), 1358–1368. https://doi.org/10.1002/jbm.b.33934

Daltoé, F. P., Mendonça, P. P., Mantesso, A., & Deboni, M. C. Z. (2014). Can SHED or DPSCs be used to repair/regenerate non-dental tissues? A systematic review of in vivo studies. *Brazilian Oral Research*, *28*, S1806-83242014000100400. https://doi.org/10.1590/1807-3107bor-2014.vol28.0037

de Assis, E. L., Silveira, F. D., da Ponte, A. V. A., & Regis, R. R. (2022). A Systematic Review of the Potential Effects of Lippia sidoides on Dental Plaque and Periodontal Diseases. *Planta Medica*, *88*(5), 341–355. https://doi.org/10.1055/a-1554-6947

Del Fabbro, M., Lolato, A., Bucchi, C., Taschieri, S., & Weinstein, R. (2016). Autologous Platelet Concentrates for Pulp and Dentin Regeneration: A Literature Review of Animal Studies. *JOURNAL OF ENDODONTICS*, *42*(2), 250–257. https://doi.org/10.1016/j.joen.2015.10.012

Delgado-Ruiz, R., Guirado, J., & Romanos, G. (2018). Bone grafting materials in critical defects in rabbit calvariae. A systematic review and quality evaluation using ARRIVE guidelines. *CLINICAL ORAL IMPLANTS RESEARCH*, *29*(6), 620–634. https://doi.org/10.1111/clr.12614

Dereka, X., Calciolari, E., Donos, N., & Mardas, N. (2018). Osseointegration in osteoporotic-like condition: A systematic review of preclinical studies. *Journal of Periodontal Research*, *53*(6), 933–940. Scopus. https://doi.org/10.1111/jre.12566

Dhingra, K., & Vandana, K. L. (2010). Prophylactic vaccination against periodontal disease: A systematic review of preclinical studies. *Journal of Periodontology*, *81*(11), 1529–1546. https://doi.org/10.1902/jop.2010.100138

Dini, C., Nagay, B. E., Magno, M. B., Maia, L. C., & Barão, V. A. R. (2020). Photofunctionalization as a suitable approach to improve the osseointegration of implants in animal models-A systematic review and meta-analysis. *Clinical Oral Implants Research*, *31*(9), 785–802. https://doi.org/10.1111/clr.13627

Dos Santos, R. L., Pithon, M. M., Lacerda, M. C. M., Ruellas, A. C. D. O., & Maia, L. C. (2012). Does the use of tacrolimus influence alveolar bone metabolism? *International Journal of Endocrinology and Metabolism*, *10*(1), 429–434. Scopus. https://doi.org/10.5812/ijem.2555

Dragonas, P., Katsaros, T., Schiavo, J., Galindo-Moreno, P., & Avila-Ortiz, G. (2020). Osteogenic capacity of the sinus membrane following maxillary sinus augmentation procedures: A systematic review. *INTERNATIONAL JOURNAL OF ORAL IMPLANTOLOGY*, *13*(3), 213–232.

Dziedzic, D. S. M., Mogharbel, B. F., Ferreira, P. E., Irioda, A. C., & de Carvalho, K. A. T. (2019). Transplantation of Adipose-derived Cells for Periodontal Regeneration: A Systematic Review. *Current Stem Cell Research & Therapy*, *14*(6), 504–518. https://doi.org/10.2174/1574888X13666181105144430

Emara, R., Elhennawy, K., & Schwendicke, F. (2018). Effects of calcium silicate cements on dental pulp cells: A systematic review. *Journal of Dentistry*, *77*, 18–36. https://doi.org/10.1016/j.jdent.2018.08.003

Faggion, C. M. J., Chambrone, L., Gondim, V., Schmitter, M., & Tu, Y.-K. (2010). Comparison of the effects of treatment of peri-implant infection in animal and human studies: Systematic review and meta-analysis. *Clinical Oral Implants Research*, *21*(2), 137–147. https://doi.org/10.1111/j.1600-0501.2009.01753.x

Fang, J., Li, Y., Zhang, K., Zhao, Z., & Mei, L. (2016). Escaping the Adverse Impacts of NSAIDs on Tooth Movement During Orthodontics: Current Evidence Based on a Meta-Analysis. *Medicine*, *95*(16), e3256. https://doi.org/10.1097/MD.0000000000003256

Fawzy El-Sayed, K. M., Ahmed, G. M., Abouauf, E. A., & Schwendicke, F. (2019). Stem/progenitor cell-mediated pulpal tissue regeneration: A systematic review and meta-analysis. *International Endodontic Journal*, *52*(11), 1573–1585. https://doi.org/10.1111/iej.13177

Fawzy El-Sayed, K. M., Jakusz, K., Jochens, A., Dörfer, C., & Schwendicke, F. (2015). Stem Cell Transplantation for Pulpal Regeneration: A Systematic Review. *Tissue Engineering. Part B, Reviews*, *21*(5), 451–460. https://doi.org/10.1089/ten.TEB.2014.0675

Fernández-González, F. J., Cañigral, A., Balbontín-Ayala, F., Gonzalo-Orden, J. M., Carlos, F. de, Cobo, T., Fernández-Vázquez, J. P., Sánchez-Lasheras, F., & Vega, J. A. (2015). Experimental evidence of pharmacological management of anchorage in Orthodontics: A systematic review. *Dental Press Journal of Orthodontics*, *20*(5), 58–65. https://doi.org/10.1590/2177-6709.20.5.058-065.oar

Fernandez-Olarte, H., Gomez-Delgado, A., Lopez-Davila, D., Rangel-Perdomo, R., Lafaurie, G., & Chambrone, L. (2017). Is the Mandibular Growth Affected by Internal Rigid Fixation?: A Systematic Review. *JOURNAL OF MAXILLOFACIAL & ORAL SURGERY*, *16*(3), 277–283. https://doi.org/10.1007/s12663-016-0968-5

Fliefel, R., Kühnisch, J., Ehrenfeld, M., & Otto, S. (2017). Gene Therapy for Bone Defects in Oral and Maxillofacial Surgery: A Systematic Review and Meta-Analysis of Animal Studies. *Stem Cells and Development*, *26*(4), 215–230. https://doi.org/10.1089/scd.2016.0172

Freires, I. A., Santaella, G. M., de Cássia Orlandi Sardi, J., & Rosalen, P. L. (2018). The alveolar bone protective effects of natural products: A systematic review. *Archives of Oral Biology*, *87*, 196–203. https://doi.org/10.1016/j.archoralbio.2017.12.019

Fukushima, K. A., Marques, M. M., Tedesco, T. K., Carvalho, G. L., Gonçalves, F., Caballero-Flores, H., Morimoto, S., & Moreira, M. S. (2019). Screening of hydrogel-based scaffolds for dental pulp regeneration-A systematic review. *Archives of Oral Biology*, *98*, 182–194. https://doi.org/10.1016/j.archoralbio.2018.11.023

Gaikwad, A. M., Joshi, A. A., Padhye, A. M., & Nadgere, J. B. (2021). Autogenous bone ring for vertical bone augmentation procedure with simultaneous implant placement: A systematic review of histologic and histomorphometric outcomes in animal studies. *The Journal of Prosthetic Dentistry*, *126*(5), 626–635. https://doi.org/10.1016/j.prosdent.2020.09.001

Ghanem, A., Pasumarthy, S., Ranna, V., Kellesarian, S. V., Abduljabbar, T., Vohra, F., & Malmstrom, H. (2016). Is mechanical curettage with adjunct photodynamic therapy more effective in the treatment of peri-implantitis than mechanical curettage alone? *Photodiagnosis and Photodynamic Therapy*, *15*, 191–196. Scopus. https://doi.org/10.1016/j.pdpdt.2016.06.007

Gharpure, A. S., & Bhatavadekar, N. B. (2018). Clinical Efficacy of Tooth-Bone Graft: A Systematic Review and Risk of Bias Analysis of Randomized Control Trials and Observational Studies. *Implant Dentistry*, *27*(1), 119–134. https://doi.org/10.1097/ID.0000000000000687

Gintautaitė, G., Kenstavičius, G., & Gaidytė, A. (2018). Dental roots’ and surrounding structures’ response after contact with orthodontic mini implants: A systematic literature review. *Stomatologija*, *20*(3), 73–81.

Gkantidis, N., Christou, P., & Topouzelis, N. (2010). The orthodontic-periodontic interrelationship in integrated treatment challenges: A systematic review. *Journal of Oral Rehabilitation*, *37*(5), 377–390. https://doi.org/10.1111/j.1365-2842.2010.02068.x

Gómez-Moreno, G., Aguilar-Salvatierra, A., Boquete-Castro, A., Guardia, J., Piattelli, A., Perrotti, V., Delgado-Ruiz, R. A., & Calvo-Guirado, J. L. (2015). Outcomes of topical applications of melatonin in implant dentistry: A systematic review. *Implant Dentistry*, *24*(1), 25–30. https://doi.org/10.1097/ID.0000000000000186

Gupta, B., Singh, S., Tallents, R. H., & Rossouw, E. (2017). Effects of Bisphosphonates on Orthodontic Treatment and the TMJ: A Systematic Review. *Journal of Clinical Orthodontics : JCO*, *51*(8), 471–478.

Haugland, L., Kristensen, K. D., Lie, S. A., & Vandevska-Radunovic, V. (2018). The effect of biologic factors and adjunctive therapies on orthodontically induced inflammatory root resorption: A systematic review and meta-analysis. *European Journal of Orthodontics*, *40*(3), 326–336. https://doi.org/10.1093/ejo/cjy003

Helgeland, E., Shanbhag, S., Pedersen, T. O., Mustafa, K., & Rosén, A. (2018). Scaffold-Based Temporomandibular Joint Tissue Regeneration in Experimental Animal Models: A Systematic Review. *Tissue Engineering. Part B, Reviews*, *24*(4), 300–316. https://doi.org/10.1089/ten.TEB.2017.0429

Hosseinpour, S., Ghazizadeh Ahsaie, M., Rezai Rad, M., Baghani, M. T., Motamedian, S. R., & Khojasteh, A. (2017). Application of selected scaffolds for bone tissue engineering: A systematic review. *Oral and Maxillofacial Surgery*, *21*(2), 109–129. https://doi.org/10.1007/s10006-017-0608-3

Hosseinpour, S., Rad, M. R., Khojasteh, A., & Zadeh, H. H. (2018). Antibody Administration for Bone Tissue Engineering: A Systematic Review. *Current Stem Cell Research & Therapy*, *13*(4), 292–315. https://doi.org/10.2174/1574888X13666180207095314

Hu, L., Zhou, M., Young, A., Zhao, W., & Yan, Z. (2019). In vivo effectiveness and safety of probiotics on prophylaxis and treatment of oral candidiasis: A systematic review and meta-analysis. *BMC Oral Health*, *19*(1), 140. https://doi.org/10.1186/s12903-019-0841-2

Iglesias-Linares, A., Yáñez-Vico, R.-M., Solano-Reina, E., Torres-Lagares, D., & González Moles, M. A. (2010). Influence of bisphosphonates in orthodontic therapy: Systematic review. *Journal of Dentistry*, *38*(8), 603–611. https://doi.org/10.1016/j.jdent.2010.05.012

Jamali, S., Khosravi, S., Shadmanpour, M., Gharibpour, F., Payahoo, S., & Darvish, M. (2020). Hyalinization and molecular pathways involved in orthodontic tooth movement: A systematic review and meta-analysis. *Pesquisa Brasileira Em Odontopediatria e Clinica Integrada*, *20*, 1–12. Scopus. https://doi.org/10.1590/pboci.2020.148

Javed, F., Ahmed, H. B., Zafar, M. S., Shaikh, M. S., Rossouw, P. E., Michelogiannakis, D., & Alstergren, P. (2022). “Testosterone decreases temporomandibular joint nociception”- A systematic review of studies on animal models. *Archives of Oral Biology*, *139*, 105430. https://doi.org/10.1016/j.archoralbio.2022.105430

Javed, F., Al Amri, M. D., Kellesarian, S. V., Al-Askar, M., Al-Kheraif, A. A., & Romanos, G. E. (2016). Laminin coatings on implant surfaces promote osseointegration: Fact or fiction? *Archives of Oral Biology*, *68*, 153–161. https://doi.org/10.1016/j.archoralbio.2016.05.005

Javed, F., Al Amri, M. D., Kellesarian, S. V., Al-Kheraif, A. A., Vohra, F., Calvo-Guirado, J. L., Malmstrom, H., & Romanos, G. E. (2016). Efficacy of parathyroid hormone supplementation on the osseointegration of implants: A systematic review. *Clinical Oral Investigations*, *20*(4), 649–658. https://doi.org/10.1007/s00784-015-1691-1

Javed, F., Bello-Correa, F. O., Nikolaidou, A., Rossouw, P. E., & Michelogiannakis, D. (2021). Anti-nociceptive efficacy of essential oil-based extracts for the management of orofacial pain: A systematic review of available evidence. *European Review for Medical and Pharmacological Sciences*, *25*(23), 7323–7332. https://doi.org/10.26355/eurrev_202112_27426

Javed, F., Kellesarian, S. V., Abduljabbar, T., Abduljabbar, A. T., Akram, Z., Vohra, F., Rahman, I., & Romanos, G. E. (2018). Influence of involuntary cigarette smoke inhalation on osseointegration: A systematic review and meta-analysis of preclinical studies. *International Journal of Oral and Maxillofacial Surgery*, *47*(6), 764–772. Scopus. https://doi.org/10.1016/j.ijom.2017.11.009

Javed, F., Kellesarian, S. V., Abduljabbar, T., Gholamiazizi, E., Feng, C., Aldosary, K., Vohra, F., & Romanos, G. E. (2017). Role of laser irradiation in direct pulp capping procedures: A systematic review and meta-analysis. *Lasers in Medical Science*, *32*(2), 439–448. https://doi.org/10.1007/s10103-016-2077-6

Jegoux, F., Malard, O., Goyenvalle, E., Aguado, E., & Daculsi, G. (2010). Radiation effects on bone healing and reconstruction: Interpretation of the literature. *Oral Surgery, Oral Medicine, Oral Pathology, Oral Radiology, and Endodontics*, *109*(2), 173–184. https://doi.org/10.1016/j.tripleo.2009.10.001

Joss-Vassalli, I., Grebenstein, C., Topouzelis, N., Sculean, A., & Katsaros, C. (2010). Orthodontic therapy and gingival recession: A systematic review. *Orthodontics & Craniofacial Research*, *13*(3), 127–141. https://doi.org/10.1111/j.1601-6343.2010.01491.x

Jyothish, S., Athanasiou, A. E., Makrygiannakis, M. A., & Kaklamanos, E. G. (2021). Effect of nicotine exposure on the rate of orthodontic tooth movement: A meta-analysis based on animal studies. *PloS One*, *16*(2), e0247011. https://doi.org/10.1371/journal.pone.0247011

Kaklamanos, E. G., Makrygiannakis, M. A., & Athanasiou, A. E. (2020). Do analgesics used for the pain experienced after orthodontic procedures affect tooth movement rate? A systematic review based on animal studies. *Orthodontics & Craniofacial Research*, *23*(2), 143–150. https://doi.org/10.1111/ocr.12357

Kaklamanos, E. G., Makrygiannakis, M. A., & Athanasiou, A. E. (2021a). Could medications and biologic factors affect post-orthodontic tooth movement changes? A systematic review of animal studies. *Orthodontics & Craniofacial Research*, *24*(1), 39–51. https://doi.org/10.1111/ocr.12411

Kaklamanos, E. G., Makrygiannakis, M. A., & Athanasiou, A. E. (2021b). Does exogenous female sex hormone administration affect the rate of tooth movement and root resorption? A systematic review of animal studies. *PloS One*, *16*(9), e0257778. https://doi.org/10.1371/journal.pone.0257778

Kalatzis-Sousa, N. G., Spin-Neto, R., Wenzel, A., Tanomaru-Filho, M., & Faria, G. (2017). Use of micro-computed tomography for the assessment of periapical lesions in small rodents: A systematic review. *International Endodontic Journal*, *50*(4), 352–366. https://doi.org/10.1111/iej.12633

Karamani, I. I., Tsolakis, I. A., Makrygiannakis, M. A., Georgaki, M., & Tsolakis, A. I. (2022). Impact of Diet Consistency on the Mandibular Morphology: A Systematic Review of Studies on Rat Models. *International Journal of Environmental Research and Public Health*, *19*(5). https://doi.org/10.3390/ijerph19052706

Katsamakis, S., Slot, D. E., Van der Sluis, L. W. M., & Van der Weijden, F. (2013). Histological responses of the periodontium to MTA: a systematic review. *Journal of Clinical Periodontology*, *40*(4), 334–344. https://doi.org/10.1111/jcpe.12058

Kellesarian, S., Abdullabbar, T., Vohra, F., Malignaggi, V., Malmstrom, H., Romanos, G., & Javed, F. (2017). Role of local alendronate delivery on the osseointegration of implants: A systematic review and meta-analysis. *INTERNATIONAL JOURNAL OF ORAL AND MAXILLOFACIAL SURGERY*, *46*(7), 912–921. https://doi.org/10.1016/j.ijom.2017.03.009

Kellesarian, S. V., Al Amri, M. D., Al-Kheraif, A. A., Ghanem, A., Malmstrom, H., & Javed, F. (2017). Efficacy of Local and Systemic Statin Delivery on the Osseointegration of Implants: A Systematic Review. *The International Journal of Oral & Maxillofacial Implants*, *32*(3), 497–506. https://doi.org/10.11607/jomi.4955

Kellesarian, S. V., Malignaggi, V. R., Kellesarian, T. V., Bashir Ahmed, H., & Javed, F. (2018). Does incorporating collagen and chondroitin sulfate matrix in implant surfaces enhance osseointegration? A systematic review and meta-analysis. *International Journal of Oral and Maxillofacial Surgery*, *47*(2), 241–251. https://doi.org/10.1016/j.ijom.2017.10.010

Kellesarian, S. V., Yunker, M., Ramakrishnaiah, R., Malmstrom, H., Kellesarian, T. V., Ros Malignaggi, V., & Javed, F. (2017). Does incorporating zinc in titanium implant surfaces influence osseointegration? A systematic review. *Journal of Prosthetic Dentistry*, *117*(1), 41–47. Scopus. https://doi.org/10.1016/j.prosdent.2016.06.003

Khojasteh, A., Soheilifar, S., Mohajerani, H., & Nowzari, H. (2013). The effectiveness of barrier membranes on bone regeneration in localized bony defects: A systematic review. *The International Journal of Oral & Maxillofacial Implants*, *28*(4), 1076–1089. https://doi.org/10.11607/jomi.2925

Kim, S. G. (2021). A cell-based approach to dental pulp regeneration using mesenchymal stem cells: A scoping review. *International Journal of Molecular Sciences*, *22*(9). Scopus. https://doi.org/10.3390/ijms22094357

Koletsi, D., Iliadi, A., Papageorgiou, S., Konrad, D., & Eliades, T. (2020). Evidence on the effect of uncontrolled diabetes mellitus on orthodontic tooth movement. A systematic review with meta-analyses in pre-clinical in-vivo research. *ARCHIVES OF ORAL BIOLOGY*, *115*. https://doi.org/10.1016/j.archoralbio.2020.104739

Kommuri, K., Javed, F., Akram, Z., & Khan, J. (2020). Effect of statins on orthodontic tooth movement: A systematic review of animal and clinical studies. *ARCHIVES OF ORAL BIOLOGY*, *111*. https://doi.org/10.1016/j.archoralbio.2020.104665

Kulkarni, S., Meer, M., & George, R. (2019). Efficacy of photobiomodulation on accelerating bone healing after tooth extraction: A systematic review. *Lasers in Medical Science*, *34*(4), 685–692. https://doi.org/10.1007/s10103-018-2641-3

Kuroshima, S., Sasaki, M., Murata, H., & Sawase, T. (2019). Medication-related osteonecrosis of the jaw-like lesions in rodents: A comprehensive systematic review and meta-analysis. *Gerodontology*, *36*(4), 313–324. https://doi.org/10.1111/ger.12416

Labussiere, M., Badran, Z., Rethore, G., Verner, C., Soueidan, A., & Struillou, X. (2021). Combination of bone substitutes and vectors in periodontology and implantology: A systematic review. *Dental Materials Journal*, *40*(4), 839–852. https://doi.org/10.4012/dmj.2020-361

Lafuente Ibáñez de Mendoza, I., Maritxalar Mendia, X., García de la Fuente, A. M., Quindós Andrés, G., & Aguirre Urizar, J. M. (2020). Role of Porphyromonas gingivalis in oral squamous cell carcinoma development: A systematic review. *Journal of Periodontal Research*, *55*(1), 13–22. https://doi.org/10.1111/jre.12691

Laugisch, O., Cosgarea, R., Nikou, G., Nikolidakis, D., Donos, N., Salvi, G. E., Stavropoulos, A., Jepsen, S., & Sculean, A. (2019). Histologic evidence of periodontal regeneration in furcation defects: A systematic review. *Clinical Oral Investigations*, *23*(7), 2861–2906. https://doi.org/10.1007/s00784-019-02964-3

Lee, J.-H., Han, S.-S., Lee, C., Kim, Y. H., & Battulga, B. (2019). Microarchitectural changes in the mandibles of ovariectomized rats: A systematic review and meta-analysis. *BMC Oral Health*, *19*(1), 128. https://doi.org/10.1186/s12903-019-0799-0

Lemes, C. H. J., da Rosa, W. L. de O., Sonego, C. L., Lemes, B. J., Moraes, R. R., & da Silva, A. F. (2019). Does laser therapy improve the wound healing process after tooth extraction? A systematic review. *Wound Repair and Regeneration : Official Publication of the Wound Healing Society [and] the European Tissue Repair Society*, *27*(1), 102–113. https://doi.org/10.1111/wrr.12678

Li, Z., Zhou, J., & Chen, S. (2021). The effectiveness of locally injected platelet-rich plasma on orthodontic tooth movement acceleration. *The Angle Orthodontist*, *91*(3), 391–398. https://doi.org/10.2319/061320-544.1

Lollobrigida, M., Fortunato, L., Lamazza, L., Serafini, G., & De Biase, A. (2020). Reosseointegration after the surgical treatment of induced peri-implantitis: Systematic review on current evidence and translation from the animal to the human model. *Minerva Stomatologica*, *69*(1), 37–54. https://doi.org/10.23736/S0026-4970.19.04181-5

Lozano-Carrascal, N., Salomó-Coll, O., Hernández-Alfaro, F., Gehrke, S.-A., Gargallo-Albiol, J., & Calvo-Guirado, J.-L. (2017). Do topical applications of bisphosphonates improve bone formation in oral implantology? A systematic review. *Medicina Oral, Patologia Oral y Cirugia Bucal*, *22*(4), e512–e519. https://doi.org/10.4317/medoral.21887

Luiz de Oliveira da Rosa, W., Machado da Silva, T., Fernando Demarco, F., Piva, E., & Fernandes da Silva, A. (2017). Could the application of bioactive molecules improve vital pulp therapy success? A systematic review. *Journal of Biomedical Materials Research. Part A*, *105*(3), 941–956. https://doi.org/10.1002/jbm.a.35968

Madi, M., Htet, M., Zakaria, O., Alagl, A., & Kasugai, S. (2018). Re-osseointegration of Dental Implants After Periimplantitis Treatments: A Systematic Review. *IMPLANT DENTISTRY*, *27*(1), 101–110. https://doi.org/10.1097/ID.0000000000000712

Mahgoub, N., Alqadasi, B., Aldhorae, K., Assiry, A., Altawili, Z., & Hong, T. (2019). Comparison between iRoot BP Plus (EndoSequence Root Repair Material) and Mineral Trioxide Aggregate as Pulp-capping Agents: A Systematic Review. *JOURNAL OF INTERNATIONAL SOCIETY OF PREVENTIVE AND COMMUNITY DENTISTRY*, *9*(6), 542–552. https://doi.org/10.4103/jispcd.JISPCD_249_19

Makrygiannakis, M. A., Kaklamanos, E. G., & Athanasiou, A. E. (2018). Does common prescription medication affect the rate of orthodontic tooth movement? A systematic review. *European Journal of Orthodontics*, *40*(6), 649–659. https://doi.org/10.1093/ejo/cjy001

Makrygiannakis, M. A., Kaklamanos, E. G., & Athanasiou, A. E. (2019a). Does long-term use of pain relievers have an impact on the rate of orthodontic tooth movement? A systematic review of animal studies. *European Journal of Orthodontics*, *41*(5), 468–477. https://doi.org/10.1093/ejo/cjy079

Makrygiannakis, M. A., Kaklamanos, E. G., & Athanasiou, A. E. (2019b). Effects of systemic medication on root resorption associated with orthodontic tooth movement: A systematic review of animal studies. *European Journal of Orthodontics*, *41*(4), 346–359. https://doi.org/10.1093/ejo/cjy048

Manzano, G., Herrero, L. R., & Montero, J. (2014). Comparison of clinical performance of zirconia implants and titanium implants in animal models: A systematic review. *The International Journal of Oral & Maxillofacial Implants*, *29*(2), 311–320. https://doi.org/10.11607/jomi.2817

Marei, H. F., Mahmood, K., & Almas, K. (2018). Critical Size Defects for Bone Regeneration Experiments in the Dog Mandible: A Systematic Review. *Implant Dentistry*, *27*(1), 135–141. https://doi.org/10.1097/ID.0000000000000713

Meng, M., Chen, Y., Ren, H., Zhang, Q., Chen, S., Zhou, X., & Zou, J. (2021). Effect of tetracyclines on pulpal and periodontal healing after tooth replantation: A systematic review of human and animal studies. *BMC Oral Health*, *21*(1), 289. https://doi.org/10.1186/s12903-021-01615-y

Meng, M., Yang, M., Lv, C., Yang, Q., Yang, Z., & Chen, S. (2017). Effect of Low-Level Laser Therapy on Relapse of Rotated Teeth: A Systematic Review of Human and Animal Study. *Photomedicine and Laser Surgery*, *35*(1), 3–11. https://doi.org/10.1089/pho.2015.4069

Meursinge Reynders, R., Ladu, L., Ronchi, L., Di Girolamo, N., de Lange, J., Roberts, N., & Plüddemann, A. (2016). Insertion torque recordings for the diagnosis of contact between orthodontic mini-implants and dental roots: A systematic review. *Systematic Reviews*, *5*, 50. https://doi.org/10.1186/s13643-016-0227-3

Michelogiannakis, D., Al-Shammery, D., Akram, Z., Rossouw, P. E., Javed, F., & Romanos, G. E. (2019). Influence of low-level laser therapy on orthodontically-induced inflammatory root resorption. A systematic review. *Archives of Oral Biology*, *100*, 1–13. https://doi.org/10.1016/j.archoralbio.2019.01.017

Michelogiannakis, D., Rossouw, P. E., Al-Shammery, D., Akram, Z., Khan, J., Romanos, G. E., & Javed, F. (2018). Influence of nicotine on orthodontic tooth movement: A systematic review of experimental studies in rats. *Archives of Oral Biology*, *93*, 66–73. https://doi.org/10.1016/j.archoralbio.2018.05.016

Mohammed, A. O., & Kaklamanos, E. G. (2021). Effect of ovariectomy-induced osteoporosis on the amount of orthodontic tooth movement: A systematic review of animal studies. *European Journal of Orthodontics*, *43*(6), 672–681. https://doi.org/10.1093/ejo/cjab013

Monsarrat, P., Vergnes, J.-N., Nabet, C., Sixou, M., Snead, M. L., Planat-Bénard, V., Casteilla, L., & Kémoun, P. (2014). Concise review: Mesenchymal stromal cells used for periodontal regeneration: A systematic review. *Stem Cells Translational Medicine*, *3*(6), 768–774. https://doi.org/10.5966/sctm.2013-0183

Monteiro, J. L. G. C., Guastaldi, F. P. S., Troulis, M. J., McCain, J. P., & Vasconcelos, B. C. do E. (2021). Induction, Treatment, and Prevention of Temporomandibular Joint Ankylosis-A Systematic Review of Comparative Animal Studies. *Journal of Oral and Maxillofacial Surgery : Official Journal of the American Association of Oral and Maxillofacial Surgeons*, *79*(1), 109-132.e6. https://doi.org/10.1016/j.joms.2020.07.018

Morad, G., Kheiri, L., & Khojasteh, A. (2013). Dental pulp stem cells for in vivo bone regeneration: A systematic review of literature. *ARCHIVES OF ORAL BIOLOGY*, *58*(12), 1818–1827. https://doi.org/10.1016/j.archoralbio.2013.08.011

Moraschini, V., Almeida, D. C. F., Calasans-Maia, J. A., & Diuana Calasans-Maia, M. (2018). The ability of topical and systemic statins to increase osteogenesis around dental implants: A systematic review of histomorphometric outcomes in animal studies. *International Journal of Oral and Maxillofacial Surgery*, *47*(8), 1070–1078. Scopus. https://doi.org/10.1016/j.ijom.2017.12.009

Moreno-Rabie, C., Gaeta-Araujo, H., Oliveira-Santos, C., Politis, C., & Jacobs, R. (2020). Early imaging signs of the use of antiresorptive medication and MRONJ: a systematic review. *CLINICAL ORAL INVESTIGATIONS*, *24*(9), 2973–2989. https://doi.org/10.1007/s00784-020-03423-0

Mourya, A., Mishra, S. K., Gaddale, R., & Chowdhary, R. (2019). Socket-shield technique for implant placement to stabilize the facial gingival and osseous architecture: A systematic review. *Journal of Investigative and Clinical Dentistry*, *10*(4), e12449. https://doi.org/10.1111/jicd.12449

Muniz, F. W. M. G., Melo, I. M., Rösing, C. K., de Andrade, G. M., Martins, R. S., Moreira, M. M. S. M., & Carvalho, R. de S. (2018). Use of antidepressive agents as a possibility in the management of periodontal diseases: A systematic review of experimental studies. *Journal of Investigative and Clinical Dentistry*, *9*(1). https://doi.org/10.1111/jicd.12291

Naert, I., Duyck, J., & Vandamme, K. (2012). Occlusal overload and bone/implant loss. *Clinical Oral Implants Research*, *23 Suppl 6*, 95–107. https://doi.org/10.1111/j.1600-0501.2012.02550.x

Najeeb, S., Siddiqui, F., Khurshid, Z., Zohaib, S., Zafar, M. S., & Ansari, S. A. (2017). Effect of bisphosphonates on root resorption after tooth replantation—A systematic review. *Dental Traumatology : Official Publication of International Association for Dental Traumatology*, *33*(2), 77–83. https://doi.org/10.1111/edt.12316

Najeeb, S., Siddiqui, F., Qasim, S. B., Khurshid, Z., Zohaib, S., & Zafar, M. S. (2017). Influence of uncontrolled diabetes mellitus on periodontal tissues during orthodontic tooth movement: A systematic review of animal studies. *Progress in Orthodontics*, *18*(1). Scopus. https://doi.org/10.1186/s40510-017-0159-z

Nibali, L., Almofareh, S. A., Bayliss-Chapman, J., Zhou, Y., Vieira, A. R., & Divaris, K. (2020). Heritability of periodontitis: A systematic review of evidence from animal studies. *Archives of Oral Biology*, *109*, 104592. https://doi.org/10.1016/j.archoralbio.2019.104592

Omar, M., & Kaklamanos, E. G. (2020). Does the rate of orthodontic tooth movement change during pregnancy and lactation? A systematic review of the evidence from animal studies. *BMC Oral Health*, *20*(1), 237. https://doi.org/10.1186/s12903-020-01223-2

Osorio Parra, M. M., Elangovan, S., & Lee, C.-T. (2019). Specialized pro-resolving lipid mediators in experimental periodontitis: A systematic review. *Oral Diseases*, *25*(5), 1265–1276. https://doi.org/10.1111/odi.12979

Pattanaik, B., Pawar, S., & Pattanaik, S. (2012). Biocompatible implant surface treatments. *Indian Journal of Dental Research : Official Publication of Indian Society for Dental Research*, *23*(3), 398–406. https://doi.org/10.4103/0970-9290.102240

Peron, D., Bergamo, A., Prates, R., Vieira, S. S., de Tarso Camillo de Carvalho, P., & Serra, A. J. (2019). Photodynamic antimicrobial chemotherapy has an overt killing effect on periodontal pathogens? A systematic review of experimental studies. *Lasers in Medical Science*, *34*(8), 1527–1534. https://doi.org/10.1007/s10103-019-02806-4

Pesce, P., Menini, M., Santori, G., De Giovanni, E., Bagnasco, F., & Canullo, L. (2020). Photo and plasma activation of dental implant titanium surfaces. A systematic review with meta-analysis of pre-clinical studies. *Journal of Clinical Medicine*, *9*(9), 1–19. Scopus. https://doi.org/10.3390/jcm9092817

Pesce, P., Menini, M., Tealdo, T., Bevilacqua, M., Pera, F., & Pera, P. (2014). Peri-implantitis: A systematic review of recently published papers. *The International Journal of Prosthodontics*, *27*(1), 15–25. https://doi.org/10.11607/ijp.3785

Piancino, M. G., Isola, G., Cannavale, R., Cutroneo, G., Vermiglio, G., Bracco, P., & Anastasi, G. P. (2017). From periodontal mechanoreceptors to chewing motor control: A systematic review. *Archives of Oral Biology*, *78*, 109–121. https://doi.org/10.1016/j.archoralbio.2017.02.010

Piancino, M. G., Tortarolo, A., Polimeni, A., Cannavale, R., Tonni, I., & Deregibus, A. (2019). Adverse effects of the bite-raised condition in animal studies: A systematic review. *Archives of Oral Biology*, *107*, 104516. https://doi.org/10.1016/j.archoralbio.2019.104516

Pilalas, I., Tsalikis, L., & Tatakis, D. N. (2016). Pre-restorative crown lengthening surgery outcomes: A systematic review. *Journal of Clinical Periodontology*, *43*(12), 1094–1108. https://doi.org/10.1111/jcpe.12617

Pinheiro, L. S., Kopper, P. M. P., Quintana, R. M., Scarparo, R. K., & Grecca, F. S. (2021). Does MTA provide a more favourable histological response than other materials in the repair of furcal perforations? A systematic review. *International Endodontic Journal*, *54*(12), 2195–2218. https://doi.org/10.1111/iej.13617

Portron, S., Soueidan, A., Marsden, A.-C., Rakic, M., Verner, C., Weiss, P., Badran, Z., & Struillou, X. (2019). Periodontal regenerative medicine using mesenchymal stem cells and biomaterials: A systematic review of pre-clinical studies. *Dental Materials Journal*, *38*(6), 867–883. https://doi.org/10.4012/dmj.2018-315

Poubel, V. L. do N., Silva, C. A. B., Mezzomo, L. A. M., De Luca Canto, G., & Rivero, E. R. C. (2018). The risk of osteonecrosis on alveolar healing after tooth extraction and systemic administration of antiresorptive drugs in rodents: A systematic review. *Journal of Cranio-Maxillo-Facial Surgery : Official Publication of the European Association for Cranio-Maxillo-Facial Surgery*, *46*(2), 245–256. https://doi.org/10.1016/j.jcms.2017.11.008

Prados-Frutos, J. C., Rodríguez-Molinero, J., Prados-Privado, M., Torres, J. H., & Rojo, R. (2016). Lack of clinical evidence on low-level laser therapy (LLLT) on dental titanium implant: A systematic review. *Lasers in Medical Science*, *31*(2), 383–392. https://doi.org/10.1007/s10103-015-1860-0

Qamruddin, I., Alam, M. K., Khamis, M. F., & Husein, A. (2015). Minimally Invasive Techniques to Accelerate the Orthodontic Tooth Movement: A Systematic Review of Animal Studies. *BioMed Research International*, *2015*, 608530. https://doi.org/10.1155/2015/608530

Rotundo, R., Bassarelli, T., Pace, E., Iachetti, G., Mervelt, J., & Pini Prato, G. (2011). Orthodontic treatment of periodontal defects. Part II: A systematic review on human and animal studies. *Progress in Orthodontics*, *12*(1), 45–52. https://doi.org/10.1016/j.pio.2011.02.008

Rungruanganunt, P., Taylor, T., Eckert, S. E., & Karl, M. (2013). The effect of static load on dental implant survival: A systematic review. *The International Journal of Oral & Maxillofacial Implants*, *28*(5), 1218–1225. https://doi.org/10.11607/jomi.2888

Scardueli, C., Bizelli-Silveira, C., Marcantonio, R., Marcantonio, E., Stavropoulos, A., & Spin-Neto, R. (2018). Systemic administration of strontium ranelate to enhance the osseointegration of implants: Systematic review of animal studies. *INTERNATIONAL JOURNAL OF IMPLANT DENTISTRY*, *4*. https://doi.org/10.1186/s40729-018-0132-8

Scheidegger, R., Koletsi, D., & Eliades, T. (2018). The impact of dietary consistency on structural craniofacial components: Temporomandibular joint/condyle, condylar cartilage, alveolar bone and periodontal ligament. A systematic review and meta-analysis in experimental in vivo research. *Archives of Oral Biology*, *94*, 33–47. https://doi.org/10.1016/j.archoralbio.2018.06.016

Schwarz, F., Hegewald, A., & Becker, J. (2014). Impact of implant-abutment connection and positioning of the machined collar/microgap on crestal bone level changes: A systematic review. *Clinical Oral Implants Research*, *25*(4), 417–425. https://doi.org/10.1111/clr.12215

Seidel, A., Seidel, C. L., Weider, M., Junker, R., Gölz, L., & Schmetzer, H. (2020). Influence of Natural Killer Cells and Natural Killer T Cells on Periodontal Disease: A Systematic Review of the Current Literature. *International Journal of Molecular Sciences*, *21*(24). https://doi.org/10.3390/ijms21249766

Shaheen, M. Y., Basudan, A. M., de Vries, R. B., van den Beucken, J. J. J. P., Jansen, J. A., & Alghamdi, H. S. (2019). Bone Regeneration Using Antiosteoporotic Drugs in Adjunction with Bone Grafting: A Meta-Analysis. *Tissue Engineering. Part B, Reviews*, *25*(6), 500–509. https://doi.org/10.1089/ten.TEB.2019.0132

Shan, Z., Wong, K. W. F., McGrath, C., Gu, M., & Yang, Y. (2021). Comprehensive Effects of Photobiomodulation Therapy as an Adjunct to Post-orthodontic Treatment Care: A Systematic Review. *Oral Health & Preventive Dentistry*, *19*(1), 203–216. https://doi.org/10.3290/j.ohpd.b1075107

Shanbhag, S., Pandis, N., Mustafa, K., Nyengaard, J. R., & Stavropoulos, A. (2017). Alveolar bone tissue engineering in critical-size defects of experimental animal models: A systematic review and meta-analysis. *Journal of Tissue Engineering and Regenerative Medicine*, *11*(10), 2935–2949. https://doi.org/10.1002/term.2198

Shanbhag, S., Pandis, N., Mustafa, K., Nyengaard, J. R., & Stavropoulos, A. (2018). Bone tissue engineering in oral peri-implant defects in preclinical in vivo research: A systematic review and meta-analysis. *Journal of Tissue Engineering and Regenerative Medicine*, *12*(1), e336–e349. https://doi.org/10.1002/term.2412

Siddiqi, A., Khan, A. S., & Zafar, S. (2017). Thirty Years of Translational Research in Zirconia Dental Implants: A Systematic Review of the Literature. *The Journal of Oral Implantology*, *43*(4), 314–325. https://doi.org/10.1563/aaid-joi-D-17-00016

Smirani, R., Rémy, M., Devillard, R., & Naveau, A. (2020). Engineered Prevascularization for Oral Tissue Grafting: A Systematic Review. *Tissue Engineering. Part B, Reviews*, *26*(4), 383–398. https://doi.org/10.1089/ten.TEB.2020.0093

Só, B. B., Silveira, F. M., Llantada, G. S., Jardim, L. C., Calcagnotto, T., Martins, M. A. T., & Martins, M. D. (2021). Effects of osteoporosis on alveolar bone repair after tooth extraction: A systematic review of preclinical studies. *Archives of Oral Biology*, *125*, 105054. https://doi.org/10.1016/j.archoralbio.2021.105054

Song, D., Shujaat, S., Politis, C., Orhan, K., & Jacobs, R. (2022). Osseoperception following dental implant treatment: A systematic review. *Journal of Oral Rehabilitation*, *49*(5), 573–585. https://doi.org/10.1111/joor.13296

Souza-Silva, B.-N., Rodrigues, J.-L.-S. A., Moreira, J.-C., Matos, F., Cesar, C.-P.-H.-A.-R., Repeke, C.-E.-P., & Paranhos, L.-R. (2016). The influence of teriparatide in induced tooth movement: A systematic review. *Journal of Clinical and Experimental Dentistry*, *8*(5), e615–e621. Scopus. https://doi.org/10.4317/jced.52997

St Martin, J. G., Javed, F., Rossouw, P. E., & Michelogiannakis, D. (2021). Influence of mini-screw implant-assisted intrusion on orthodontically induced inflammatory root resorption: A systematic review. *European Archives of Paediatric Dentistry : Official Journal of the European Academy of Paediatric Dentistry*, *22*(3), 341–349. https://doi.org/10.1007/s40368-020-00588-w

Stavropoulos, A., Bertl, K., Winning, L., & Polyzois, I. (2021). What is the influence of implant surface characteristics and/or implant material on the incidence and progression of peri-implantitis? A systematic literature review. *Clinical Oral Implants Research*, *32*(S21), 203–229. Scopus. https://doi.org/10.1111/clr.13859

Stocchero, M., Toia, M., Cecchinato, D., Becktor, J. P., Coelho, P. G., & Jimbo, R. (2016). Biomechanical, Biologic, and Clinical Outcomes of Undersized Implant Surgical Preparation: A Systematic Review. *The International Journal of Oral & Maxillofacial Implants*, *31*(6), 1247–1263. https://doi.org/10.11607/jomi.5340

Tassi, S. A., Sergio, N. Z., Misawa, M. Y. O., & Villar, C. C. (2017). Efficacy of stem cells on periodontal regeneration: Systematic review of pre-clinical studies. *Journal of Periodontal Research*, *52*(5), 793–812. https://doi.org/10.1111/jre.12455

Teng, F., Yu, D., Wei, L., Su, N., & Liu, Y. (2019). Preclinical application of recombinant human bone morphogenetic protein 2 on bone substitutes for vertical bone augmentation: A systematic review and meta-analysis. *The Journal of Prosthetic Dentistry*, *122*(4), 355–363. https://doi.org/10.1016/j.prosdent.2018.09.008

Teughels, W., Loozen, G., & Quirynen, M. (2011). Do probiotics offer opportunities to manipulate the periodontal oral microbiota? *Journal of Clinical Periodontology*, *38 Suppl 11*, 159–177. https://doi.org/10.1111/j.1600-051X.2010.01665.x

Travassos da Rosa Moreira Bastos, R., Mecenas, P., & Normando, D. (2021). Effects of dietary consistency on the occlusal changes in nonhuman mammals: A systematic review. *Archives of Oral Biology*, *130*, 105217. https://doi.org/10.1016/j.archoralbio.2021.105217

Tretto, P. H. W., Fabris, V., Cericato, G. O., Sarkis-Onofre, R., & Bacchi, A. (2019). Does the instrument used for the implant site preparation influence the bone-implant interface? A systematic review of clinical and animal studies. *International Journal of Oral and Maxillofacial Surgery*, *48*(1), 97–107. https://doi.org/10.1016/j.ijom.2018.04.005

Trybek, G., Jedliński, M., Jaroń, A., Preuss, O., Mazur, M., & Grzywacz, A. (2020). Impact of lactoferrin on bone regenerative processes and its possible implementation in oral surgery—A systematic review of novel studies with metanalysis and metaregression. *BMC Oral Health*, *20*(1), 232. https://doi.org/10.1186/s12903-020-01211-6

Tucker, R. L., & Ha, W. N. (2021). A Systematic Review Comparing Mineral Trioxide Aggregate to Other Commercially Available Direct Pulp Capping Agents in Dogs. *Journal of Veterinary Dentistry*, *38*(1), 34–45. https://doi.org/10.1177/08987564211024905

Valle, I. B., Schuch, L. F., da Silva, J. M., Gala-García, A., Diniz, I. M. A., Birbrair, A., Abreu, L. G., & Silva, T. A. (2020). Pericyte in Oral Squamous Cell Carcinoma: A Systematic Review. *Head and Neck Pathology*, *14*(4), 1080–1091. https://doi.org/10.1007/s12105-020-01188-2

Valles, C., Rodríguez-Ciurana, X., Clementini, M., Baglivo, M., Paniagua, B., & Nart, J. (2018). Influence of subcrestal implant placement compared with equicrestal position on the peri-implant hard and soft tissues around platform-switched implants: A systematic review and meta-analysis. *Clinical Oral Investigations*, *22*(2), 555–570. https://doi.org/10.1007/s00784-017-2301-1

Vansant, L., Cadenas De Llano-Pérula, M., Verdonck, A., & Willems, G. (2018). Expression of biological mediators during orthodontic tooth movement: A systematic review. *Archives of Oral Biology*, *95*, 170–186. https://doi.org/10.1016/j.archoralbio.2018.08.003

Varela-López, A., Navarro-Hortal, M. D., Giampieri, F., Bullón, P., Battino, M., & Quiles, J. L. (2018). Nutraceuticals in Periodontal Health: A Systematic Review on the Role of Vitamins in Periodontal Health Maintenance. *Molecules (Basel, Switzerland)*, *23*(5). https://doi.org/10.3390/molecules23051226

Veginadu, P., Tavva, S. R., Muddada, V., & Gorantla, S. (2020). Effect of pharmacological agents on relapse following orthodontic tooth movement. *The Angle Orthodontist*, *90*(4), 598–606. https://doi.org/10.2319/092619-613.1

Venkatesan, K., Srinivasan, B., & Padmanabhan, S. (2021). Adverse effect of consumption of carbonated soft drinks on orthodontic treatment—A systematic review. *Indian Journal of Dental Research : Official Publication of Indian Society for Dental Research*, *32*(4), 505–513. https://doi.org/10.4103/ijdr.IJDR_647_20

Waasdorp, J. A., Evian, C. I., & Mandracchia, M. (2010). Immediate placement of implants into infected sites: A systematic review of the literature. *Journal of Periodontology*, *81*(6), 801–808. https://doi.org/10.1902/jop.2010.090706

Wang, B., Shao, J., Fu, J., Jansen, J. A., Walboomers, X. F., Hooijmans, C. R., Van Luijk, J., & Yang, F. (2019). Topical Host-Modulating Therapy for Periodontal Regeneration: A Systematic Review and Meta-Analysis. *Tissue Engineering. Part B, Reviews*, *25*(6), 526–543. https://doi.org/10.1089/ten.TEB.2019.0184

Werny, J. G., Sagheb, K., Diaz, L., Kämmerer, P. W., Al-Nawas, B., & Schiegnitz, E. (2022). Does vitamin D have an effect on osseointegration of dental implants? A systematic review. *International Journal of Implant Dentistry*, *8*(1), 16. https://doi.org/10.1186/s40729-022-00414-6

Yan, M.-D., Ou, Y.-J., Lin, Y.-J., Liu, R.-M., Fang, Y., Wu, W.-L., Zhou, L., Yao, X., & Chen, J. (2022). Does the incorporation of strontium into calcium phosphate improve bone repair? A meta-analysis. *BMC Oral Health*, *22*(1), 62. https://doi.org/10.1186/s12903-022-02092-7

Yan, X.-Z., Yang, F., Jansen, J. A., de Vries, R. B. M., & van den Beucken, J. J. J. P. (2015). Cell-Based Approaches in Periodontal Regeneration: A Systematic Review and Meta-Analysis of Periodontal Defect Models in Animal Experimental Work. *Tissue Engineering. Part B, Reviews*, *21*(5), 411–426. https://doi.org/10.1089/ten.TEB.2015.0049

Yen, C.-C., Tu, Y.-K., Chen, T.-H., & Lu, H.-K. (2014). Comparison of treatment effects of guided tissue regeneration on infrabony lesions between animal and human studies: A systematic review and meta-analysis. *Journal of Periodontal Research*, *49*(4), 415–424. https://doi.org/10.1111/jre.12130
