# Supplementary material for: Methodological quality of systematic reviews in dentistry including animal studies: a cross-sectional study
Source: Ir Vet J. 2023 Dec 14;76:33. doi: 10.1186/s13620-023-00261-w (PMC10720166; doi:10.1186/s13620-023-00261-w)
Supplement: Supplementary file 5 — Additional file 5. Systematic review characteristics. [file 13620_2023_261_MOESM5_ESM.docx]

Supplementary file 5 – Systematic review characteristics

| **h-index first author minimum** | **h-index first author maximum** | **h-index first author mean** |
| --- | --- | --- |
| 0 | 69 | 11,81 |

| **h-index last author minimum** | **h-index last author maximum** | **h-index last author mean** |
| --- | --- | --- |
| 1 | 117 | 28,98 |

| **number of authors minimum** | **number of authors maximum** | **number of authors mean** |
| --- | --- | --- |
| 1 | 9 | 4,9 |

| **IF 2021 minimum** | **IF 2021 Maximum** | **IF 2021 mean** |
| --- | --- | --- |
| 1,154 | 8,755 | 3,774 |

| **Citations minimum** | **Citations maximum** | **Citations mean** |
| --- | --- | --- |
| 0 | 493 | 38,48 |

| **Continent** | **n** | **%** |
| --- | --- | --- |
| Europe | 72 | 37,89% |
| Asia | 49 | 25,79% |
| South America | 32 | 16,84% |
| North America | 27 | 14,21% |
| Australia | 9 | 4,74% |
| Africa | 1 | 0,53% |

| **Country (or region)** | **n** | **%** |
| --- | --- | --- |
| Brazil | 30 | 15,79% |
| USA | 26 | 13,68% |
| Saudi-Arabia | 13 | 6,84% |
| Spain | 13 | 6,84% |
| China | 10 | 5,26% |
| Italy | 10 | 5,26% |
| Australia | 9 | 4,74% |
| UAE | 9 | 4,74% |
| Germany | 7 | 3,68% |
| India | 7 | 3,68% |
| Switzerland | 6 | 3,16% |
| Belgium | 6 | 3,16% |
| The Netherlands | 6 | 3,16% |
| France | 5 | 2,63% |
| Iran | 5 | 2,63% |
| Sweden | 4 | 2,11% |
| Greece | 4 | 2,11% |
| Norway | 3 | 1,58% |
| Denmark | 2 | 1,05% |
| South Korea | 1 | 0,53% |
| Chile | 1 | 0,53% |
| Colombia | 1 | 0,53% |
| Pakistan | 1 | 0,53% |
| Singapore | 1 | 0,53% |
| Taiwan | 1 | 0,53% |
| UK | 1 | 0,53% |
| Canada | 1 | 0,53% |
| Finland | 1 | 0,53% |
| Egypt | 1 | 0,53% |
| Romania | 1 | 0,53% |
| Poland | 1 | 0,53% |
| Portugal | 1 | 0,53% |
| Japan | 1 | 0,53% |
| Lithuania | 1 | 0,53% |

| **Center** | **n** | **%** |
| --- | --- | --- |
| Multi-center | 159 | 83,68% |
| Single-center | 31 | 16,32% |

| **Year of publication** | **n** | **%** |
| --- | --- | --- |
| 2018 | 32 | 16,85% |
| 2019 | 25 | 13,17% |
| 2021 | 25 | 13,18% |
| 2017 | 23 | 12,10% |
| 2020 | 22 | 11,59% |
| 2016 | 18 | 9,47% |
| 2013 | 10 | 5,25% |
| 2014 | 8 | 4,20% |
| 2010 | 8 | 4,19% |
| 2022 | 8 | 4,22% |
| 2015 | 6 | 3,15% |
| 2012 | 3 | 1,57% |
| 2011 | 2 | 1,05% |

| **Journal** | **n** | **%** |
| --- | --- | --- |
| ARCHIVES OF ORAL BIOLOGY | 18 | 9,47% |
| JOURNAL OF PERIODONTAL RESEARCH | 8 | 4,21% |
| Clinical Oral Investigations | 8 | 4,21% |
| BMC Oral Health | 8 | 4,21% |
| Tissue Engineering Part B-Reviews | 8 | 4,21% |
| INTERNATIONAL JOURNAL OF ORAL & MAXILLOFACIAL IMPLANTS | 7 | 3,68% |
| CLINICAL ORAL IMPLANTS RESEARCH | 7 | 3,68% |
| Implant Dentistry | 6 | 3,16% |
| EUROPEAN JOURNAL OF ORTHODONTICS | 6 | 3,16% |
| LASERS IN MEDICAL SCIENCE | 5 | 2,63% |
| INTERNATIONAL JOURNAL OF ORAL AND MAXILLOFACIAL SURGERY | 5 | 2,63% |
| JOURNAL OF PROSTHETIC DENTISTRY | 4 | 2,11% |
| INTERNATIONAL ENDODONTIC JOURNAL | 4 | 2,11% |
| JOURNAL OF CLINICAL PERIODONTOLOGY | 3 | 1,58% |
| Indian Journal of Dental Research | 3 | 1,58% |
| ANGLE ORTHODONTIST | 3 | 1,58% |
| Orthodontics & Craniofacial Research | 3 | 1,58% |
| INTERNATIONAL JOURNAL OF MOLECULAR SCIENCES | 3 | 1,58% |
| European Archives of Paediatric Dentistry | 3 | 1,58% |
| PHOTOMEDICINE AND LASER SURGERY | 2 | 1,05% |
| DENTAL TRAUMATOLOGY | 2 | 1,05% |
| Current Stem Cell Research & Therapy | 2 | 1,05% |
| International Journal of Implant Dentistry | 2 | 1,05% |
| ORAL DISEASES | 2 | 1,05% |
| JOURNAL OF CRANIO-MAXILLOFACIAL SURGERY | 2 | 1,05% |
| JOURNAL OF PERIODONTOLOGY | 2 | 1,05% |
| Photodiagnosis and Photodynamic Therapy | 2 | 1,05% |
| Journal of Tissue Engineering and Regenerative Medicine | 2 | 1,05% |
| JOURNAL OF ENDODONTICS | 2 | 1,05% |
| Medicina Oral Patologia Oral y Cirugia Bucal | 2 | 1,05% |
| Journal of International Society of Preventive and Community Dentistry | 2 | 1,05% |
| DENTAL MATERIALS JOURNAL | 2 | 1,05% |
| Journal of Investigative and Clinical Dentistry | 2 | 1,05% |
| JOURNAL OF ORAL REHABILITATION | 2 | 1,05% |
| JOURNAL OF DENTISTRY | 2 | 1,05% |
| PLoS One | 2 | 1,05% |
| Pesquisa Brasileira em Odontopediatria e Clinica Integrada | 1 | 0,53% |
| Minerva Stomatologica | 1 | 0,53% |
| JOURNAL OF VETERINARY DENTISTRY | 1 | 0,53% |
| PLANTA MEDICA | 1 | 0,53% |
| CELL PROLIFERATION | 1 | 0,53% |
| Progress in Orthodontics | 1 | 0,53% |
| International Journal of Endocrinology and Metabolism | 1 | 0,53% |
| STEM CELLS AND DEVELOPMENT | 1 | 0,53% |
| Dental Press Journal of Orthodontics | 1 | 0,53% |
| Stomatologija | 1 | 0,53% |
| NUTRITION REVIEWS | 1 | 0,53% |
| Biomed Research International | 1 | 0,53% |
| Oral Surgery Oral Medicine Oral Pathology Oral Radiology and Endodontology | 1 | 0,53% |
| European Review for Medical and Pharmacological Sciences | 1 | 0,53% |
| INTERNATIONAL JOURNAL OF PROSTHODONTICS | 1 | 0,53% |
| FITOTERAPIA | 1 | 0,53% |
| International Journal of Environmental Research and Public Health | 1 | 0,53% |
| GERONTOLOGY | 1 | 0,53% |
| Brazilian Oral Research | 1 | 0,53% |
| Head & Neck Pathology | 1 | 0,53% |
| MEDICINE | 1 | 0,53% |
| ANNALS OF ANATOMY-ANATOMISCHER ANZEIGER | 1 | 0,53% |
| MOLECULES | 1 | 0,53% |
| Journal of Maxillofacial & Oral Surgery | 1 | 0,53% |
| Oral and Maxillofacial Surgery-Heidelberg | 1 | 0,53% |
| JOURNAL OF ORAL AND MAXILLOFACIAL SURGERY | 1 | 0,53% |
| Oral Health & Preventive Dentistry | 1 | 0,53% |
| Journal of Oral Implantology | 1 | 0,53% |
| Clinical Implant Dentistry and Related Research | 1 | 0,53% |
| CRANIO-The Journal of Craniomandibular & Sleep Practice | 1 | 0,53% |
| International Journal of Oral Implantology | 1 | 0,53% |
| ANAIS DA ACADEMIA BRASILEIRA DE CIENCIAS | 1 | 0,53% |
| JOURNAL OF BIOMEDICAL MATERIALS RESEARCH PART A | 1 | 0,53% |
| Brazilian Dental Journal | 1 | 0,53% |
| JOURNAL OF BIOMEDICAL MATERIALS RESEARCH PART B-APPLIED BIOMATERIALS | 1 | 0,53% |
| Scientific World Journal | 1 | 0,53% |
| Systematic Reviews | 1 | 0,53% |
| Stem Cells Translational Medicine | 1 | 0,53% |
| WOUND REPAIR AND REGENERATION | 1 | 0,53% |
| Australian Endodontic Journal | 1 | 0,53% |
| AMERICAN JOURNAL OF ORTHODONTICS AND DENTOFACIAL ORTHOPEDICS | 1 | 0,53% |
| Journal of Clinical and Experimental Dentistry | 1 | 0,53% |
| Journal of Clinical Medicine | 1 | 0,53% |
| JOURNAL of CLINICAL ORTHODONTICS | 1 | 0,53% |

| **Journal Category** | **n** | **%** |
| --- | --- | --- |
| DENTISTRY, ORAL SURGERY & MEDICINE - SCIE | 122 | 64,21% |
| ENGINEERING, BIOMEDICAL - SCIE | 15 | 7,89% |
| DENTISTRY, ORAL SURGERY & MEDICINE - ESCI | 11 | 5,79% |
| Not listed in JCR | 8 | 4,21% |
| CELL BIOLOGY - SCIE | 6 | 3,16% |
| BIOCHEMISTRY & MOLECULAR BIOLOGY - SCIE | 4 | 2,11% |
| MULTIDISCIPLINARY SCIENCES - SCIE | 4 | 2,11% |
| MEDICINE, GENERAL & INTERNAL - SCIE | 3 | 1,58% |
| PHARMACOLOGY & PHARMACY - SCIE | 3 | 1,58% |
| SURGERY - SCIE | 2 | 1,05% |
| ONCOLOGY - SCIE | 2 | 1,05% |
| ENDOCRINOLOGY & METABOLISM - ESCI | 1 | 0,53% |
| NUTRITION & DIETETICS - SCIE | 1 | 0,53% |
| TRANSPLANTATION - SCIE | 1 | 0,53% |
| MEDICINE, RESEARCH & EXPERIMENTAL - SCIE | 1 | 0,53% |
| PUBLIC, ENVIRONMENTAL & OCCUPATIONAL HEALTH - SSCI | 1 | 0,53% |
| CELL & TISSUE ENGINEERING - SCIE | 1 | 0,53% |
| ANATOMY & MORPHOLOGY - SCIE | 1 | 0,53% |
| GERIATRICS & GERONTOLOGY - SCIE | 1 | 0,53% |
| PATHOLOGY - ESCI | 1 | 0,53% |
| VETERINARY SCIENCES - SCIE | 1 | 0,53% |

| **Topic** | **n** | **%** |
| --- | --- | --- |
| Oral Surgery & Implantology | 84 | 44,21% |
| Periodontology & Periodontal Surgery | 42 | 22,11% |
| Orthodontics | 38 | 20,00% |
| Conservative Dentistry & Endodontics | 24 | 12,63% |
| Prosthodontics | 2 | 1,05% |

| **COI** | **n** | **%** |
| --- | --- | --- |
| not present | 151 | 79,47% |
| No/unclear information | 37 | 19,47% |
| present | 2 | 1,05% |

| **Funding** | **n** | **%** |
| --- | --- | --- |
| no sponsor | 70 | 36,84% |
| No/unclear information | 67 | 35,26% |
| profit sector | 52 | 27,37% |
| non-profit sector | 1 | 0,53% |

| **Tool for Risk of Bias Assessment** | **n** | **%** |
| --- | --- | --- |
| SYRCLE | 71 | 29,10% |
| none | 49 | 20,08% |
| Cochrane | 33 | 13,52% |
| ARRIVE | 29 | 11,89% |
| JADAD | 8 | 3,28% |
| CASP | 8 | 3,28% |
| ROBINS-I | 6 | 2,46% |
| selfmade | 6 | 2,46% |
| CAMARADES | 5 | 2,05% |
| NOS | 5 | 2,05% |
| Antczak | 3 | 1,23% |
| MINORS | 2 | 0,82% |
| OCEBM | 2 | 0,82% |
| CONSORT | 2 | 0,82% |
| ACROBAT-NRSI | 2 | 0,82% |
| QUADAS | 1 | 0,41% |
| Based on: Bondemark et al. 2007 | 1 | 0,41% |
| University of Adelaide | 1 | 0,41% |
| Combination of MOOSE, Strobe, Verhagen, Jadad, CONSORT, ARRIVE, GSP | 1 | 0,41% |
| QATRS | 1 | 0,41% |
| Based on: The methodological quality assessment tools for preclinical and clinical studies, systematic review and meta-analysis, and clinical practice guideline: a systematic review. | 1 | 0,41% |
| JBI | 1 | 0,41% |
| Cericato | 1 | 0,41% |
| STAIR | 1 | 0,41% |
| OHAT | 1 | 0,41% |
| NIH Tool | 1 | 0,41% |
| PRISMA | 1 | 0,41% |
| Krauth | 1 | 0,41% |
