# Supplementary material for: Methodological quality of systematic reviews in dentistry including animal studies: a cross-sectional study
Source: Ir Vet J. 2023 Dec 14;76:33. doi: 10.1186/s13620-023-00261-w (PMC10720166; doi:10.1186/s13620-023-00261-w)
Supplement: Supplementary file 6 — Additional file 6. Comparison to Faggion et al. 2012. [file 13620_2023_261_MOESM6_ESM.docx]

Supplementary file 6 – Comparison to Faggion et al. 2012

| **AMSTAR-1** | | | | **AMSTAR-2** | | | |
| --- | --- | --- | --- | --- | --- | --- | --- |
| Items | Yes | No/cannot answer | Not applicable | Items | Yes/partial yes | No | Not applicable |
| **Item1 Was an 'a priori' design provided?** | 53 (98%) | 1 (2%) | 0 (0%) | **Item 2 Did the report of the review contain an explicit statement that the review methods were established prior to the conduct of the review and did the report justify any significant deviations from the protocol?** | 119 (63%) | 71 (37%) | 0 (0%) |
| **Item 3 Was a comprehensive literature search performed?** | 23 (43%) | 31 (57%) | 0 (0%) | **Item 4 Did the review authors use a comprehensive literature search strategy?** | 169 (89%) | 21 (11%) | 0 (0%) |
| **Item 2 Was there duplicate study selection and data extraction?** | 12 (22%) | 42 (78%) | 0 (0%) | **Item 5 Did the review authors perform study selection in duplicate?** | 149 (78%) | 41 (22%) | 0 (0%) |
|  |  |  |  | **Item 6 Did the review authors perform data extraction in duplicate?** | 67 (35%) | 123 (65%) | 0 (0%) |
| **Item 5 Was a list of studies (included and excluded) provided?** | 8 (15%) | 46 (85%) | 0 (0%) | **Item 7 Did the review authors provide a list of excluded studies and justify the exclusions?** | 68 (36%) | 122 (64%) | 0 (0%) |
| **Item 6 Were the characteristics of the included studies provided?** | 34 (63%) | 20 (37%) | 0 (0%) | **Item 8 Did the review authors describe the included studies in adequate detail?** | 164 (86%) | 25 (13%) | 1 (1%) |
| **Item 7 Was the scientific quality of the included studies assessed and documented?** | 5 (9%) | 49 (91%) | 0 (0%) | **Item 9 Did the review authors use a satisfactory technique for assessing the risk of bias (RoB) in individual studies that were included in the review?** | 112 (59%) | 78 (41%) | 0 (0%) |
| **Item 9 Were the methods used to combine the findings of studies appropriate?** | 2 (4%) | 0 (0%) | 52 (96%) | **Item 11 If meta-analysis was performed did the review authors use appropriate methods for statistical combination of results?** | 43 (23%) | 1 (1%) | 146 (77%) |
| **Item 8 Was the scientific quality of the included studies used appropriately in formulating conclusions?*** | 5 (9%) | 49 (91%) | 0 (0%) | **Item 13 Did the review authors account for RoB in individual studies when interpreting/ discussing the results of the review?*** | 36 (19%) | 154 (81%) | 0 (0%) |
| **Item 10 Was the likelihood of publication bias assessed?** | 1 (2%) | 53 (98%) | 0 (0%) | **Item 15 If they performed quantitative synthesis did the review authors carry out an adequate investigation of publication bias (small study bias) and discuss its likely impact on the results of the review?** | 24 (13%) | 20 (11%) | 146 (77%) |
| **Item 11 Was the conflict of interest stated?**** | 15 (28%) | 39 (77%) | 0 (0%) | **Item 16 Did the review authors report any potential sources of conflict of interest, including any funding they received for conducting the review?**** | 153 (80%) | 37 (20%) | 0 (0%) |

*: item 8 from AMSTAR-1 mainly assessed if scientific quality of the included studies were considered in the analysis and conclusions of the review, while item 13 from AMSTAR-2 mainly assessed if risk of bias of the included studies were considered when interpreting/discussing the results of the review.

**: item 11 from AMSTAR-1 assessed if conflict of interest was acknowledged in both the reviews and the included studies of the reviews, while item 16 from AMSTAR-2 only assessed if conflict of interest was acknowledged in the reviews.
